# Supplementary material for: Comparative genomics and transcriptomics of rough and smooth Mycobacterium marinum focusing on ESX-1, ESX-6 and LOS genes
Source: Sci Rep. 2026 Jul 10;16:21610. doi: 10.1038/s41598-026-61405-w (PMC13354803; doi:10.1038/s41598-026-61405-w)

Supplementary Information

Loss of *Mycobacterium marinum* ESX-1 genes increase transcription of ESX-6 genes

Phani Rama Krishna Behra[1], Malavika Ramesh[1,2], B. M. Fredrik Pettersson[1,3], and Leif A. Kirsebom[1,*]

[1]Department of Cell and Molecular Biology
Box 596, Biomedical Centre
SE-751 24 Uppsala, Sweden

[2]Current address:

[3]Current address:

*Corresponding author
Leif.kirsebom@icm.uu.se

Running title: *Mycobacterium marinum* strain variation

Key words: *M. marinum* ESX genes and genomics, transcription of ESX genes, Smooth and Rough colony morphology, LOS genes, transcription of LOS genes

Analysis of transcription of selected virulence genes under different growth conditions

The 1218R strain is more virulent than 1218S[18], and we identified the presence of virulence genes in these two and three other *M. marinum* strains (see main text). In addition to the analysis of ESX and associated gene transcripts we also examined the transcript levels of selected virulence genes to get deeper insight into the differences between 1218R and 1218S. As discussed in the main text, we analysed the transcript levels of these genes detected in exponentially growing and stationary cells and we also included $Mmar^{CCUG}$ and $Mmar^{M}$. The analysis was performed as outlined in the main text and the results are shown Figure S6 and Table S3.

As indicated the main text (Figs 2 and S4, Tables S1 and S3), relative to 1218R we detected some variation in the number of virulence genes in the four strains, $Mmar^{1218S}$ (in addition to the missing ESX-1 genes), $Mmar^{CCUG}$, $Mmar^{ATCC}$ and $Mmar^{M}$. For $Mmar^{1218R}$, $Mmar^{1218S}$, $Mmar^{CCUG}$ and $Mmar^{M}$ the overall transcript patterns of virulence genes were similar with some differences (Fig S6a-g), where the differences were more apparent comparing 1218R with in particular $Mmar^{M}$ (Fig S6g).

The mRNA levels for the well described virulence genes, *devR* (*dosR*) and *devS* (1218R gene id: 01428 and 01429) involved in regulating genes important to establish mycobacterial dormancy[102,103], were higher in 1218R than in 1218S, in particular in stationary cells. Higher levels were also detected for *devR* and *devS* in 1218R relative to $Mmar^{CCUG}$ (particularly in stationary cells; Fig 6e). Comparing 1218R and $Mmar^{M}$ revealed that the *devS* mRNA levels was higher in stationary cells while both *devR* and *devS* were higher in exponential $Mmar^{M}$ cells (Fig S6g). Moreover, the 04801-gene transcript (1218R gene id; Figs S6c, e and g; Table S3), which encodes a PE family protein[104,105], was higher in 1218R compared to 1218S (and $Mmar^{CCUG}$) while only small differences were detected compared to $Mmar^{M}$

(noteworthy, we did detect strain dependent variations in mRNA levels for several PE/PPE genes; not shown).

For *mce*6 genes (mammalian cell entry; 1218R gene ids 00168-00173)[106], the mRNA levels were modestly higher in stationary 1218S cells, albeit the differences were small (Fig S6c; Table S3), while higher levels were detected in 1218R than in *Mmar*^{CCUG} and *Mmar*^{M} (both in exponential and stationary cells; Fig S6e and g).

For *lppX* (1218R gene id 01775; putative lipoprotein, LppX, precursor)[21], no significant differences were detected comparing 1218R and 1218S or *Mmar*^{CCUG} (except that in stationary *Mmar*^{CCUG} cells the mRNA level was higher; Fig S6e). In contrast, we detected >12 log_2-fold higher mRNA levels in 1218R relative to *Mmar*^{M}. Higher levels were also seen for a putative ESAT-6-like protein (≈10 log_2-fold; 1218R gene id 03733), polyketide synthase type I Pks15/1 (>3 log_2-fold; 1218R gene id 03856) and for several other genes comparing 1218R and the M strain (Fig 6g; Table S3).

Furthermore, relative to 1218R the transcript levels for the virulence-related genes *icl* (isocitrate lyase; 1218R gene id 00746), *hspX_2* (alpha-crystallin; 1218R gene id 03552), and *eis*_2 (enhanced intracellular survival protein; 1218R gene id 03812; note that the mRNA level for this gene is higher in *Mmar*^{CCUG}) were lower in 1218S. By contrast, we noted higher mRNA levels for these genes in *Mmar*^{M}. Transcript levels of ESX-3 genes and for some phospholipase genes, *e.g.*, *plcA*_2 (1218R gene id 01384), were also higher in *Mmar*^{M}. Interestingly, for the M strain we noted higher levels for the transcription factors *sigD* (1218R gene id 01099) and *whiB*3 [in exponential growing cells (1218R gene id 01097)]. WhiB3 is suggested to be involved in the regulation of *whiB*6 expression[15] and transcript levels of *whiB*6 is discussed in the main text. For other differences see Figs S6 and Table S3).

Taken together, the transcript levels for several virulence-related genes differ depend on *Mmar* strain. Given that we did detect differential mRNA levels for several genes involved in

the formation of the outer boundaries might suggest variation in the structure of the outer membrane and thereby virulence for the different strains. For example, the four-fold difference in virulence reported for 1218R relative to 1218S[18]. Moreover, the differences observed for the M strain relative to 1218R is consistent with that these strains represent two different *M. marinum* subspecies/lineages[18,unpubl data]. As discussed in the main text, the levels of the major virulence ESX-1 *esxB*_3 (CFP-10) and *esxA*_3 (ESAT-6) transcripts are among the most abundant virulence-related gene transcripts irrespective of strain with the exception of 1218S. In 1218S the ESX-6 genes *esxB*_1 and *esxA*_1 (homologs to *esxB*_3 and *esxA*_3) are among the most abundant transcripts (see main text; Fig S7a, b, d and f).

Mmar *strains transformed with the empty control plasmid (pBS401) or with pBS401$^{espF\text{-}H}$*

The 1218R and 1218S *Mmar* strains were transformed with the empty control plasmid (pBS401) or with pBS401$^{espF\text{-}H}$ and cultivated as outlined in Materials and Methods. The *espF*_2, *espG*$_1$_2 and *espH* genes from 1218R were cloned into the pBS401 plasmid behind an anhydrotetracycline inducible promoter[100] and screened using plasmid specific primers (see Supplementary information). This construct is refered to as pBS401$^{espF\text{-}H}$. The *Mmar* 1218R and 1218S strains were transformed with an empty control plasmid (pBS401) or with pBS401$^{espF\text{-}H}$. The cells were cultivated in microtiter plates for 26 days in 7H9 medium without Tween 80 or hygromycin B. The cultures grown in the 96-well plates were used for the quantification of biofilm formation[101]. Each strain and plasmid combination were measured in two biological replicates, with two technical replicates per biological replicate; in total four measurements per combination.

Supplementary Information - References

102. Kundu, M. & Basu, J. Applications of transcriptomics and proteomics for understanding dormancy and resuscitation in Mycobacterium tuberculosis. *Front. Microbiol.* **12**, 642487 (2021).

103. Verma, A., Ghoshal, A., Dwivedi, V. P. & Bhaskar, A. Tuberculosis: The success tale of less explored dormant Mycobacterium tuberculosis. *Front. Cell Infect. Microbiol.* **12**, 1079569 (2022).

104. Qian, J., Chen, R., Wang, H. & Zhang, X. Role of the PE/PPE family in host-pathogen interactions and prospects for anti-tuberculosis vaccine and diagnostic tool design. *Front. Cell Infect. Microbiol.* **10**, 594288 (2020).

105. Xie, Y., Zhou, Y., Liu, S. & Zhang, X-L. PE_PGRS: Vital proteins in promoting mycobacterial and modulating host immunity and metabolism. *Cell Microbiol.* **23**, e13290 (2021).

106. Klepp, L. I., y Garcia, J. S. & Bigi, F. Mycobacterial MCE proteins as transporters that control lipid homeostasis of the cell wall. *Tuberculosis* **132**, 102162 (2022).

Figure legends Supplementary information

Figure S1 Genome aligment, analysis of CDS and functional classifications for 1218R, 1218S, *Mmar*^{CCUG}, *Mmar*^{ATCC927} and *Mmar*^{M}.

(a) Whole-genome alignment for the complete genomes for five *Mmar* strains. The horizontal blocks represent the genomes as marked while vertical "blocks" correspond to homologous regions. White gaps represent insertions/deletions. For details see the main text.

(b) Venn diagram showing the presence of common and unique annotated genes for the five *Mmar* strains.

(c) RAST functional classification of core genes in the five different *Mmar* strains as indicated (3138, 3071, 3075, 3105 and 3149 correspond to classification of the total number of annotated genes in the respective strain). Note that he functionally classified genes were not manually edited, see main text for details.

Figure S1

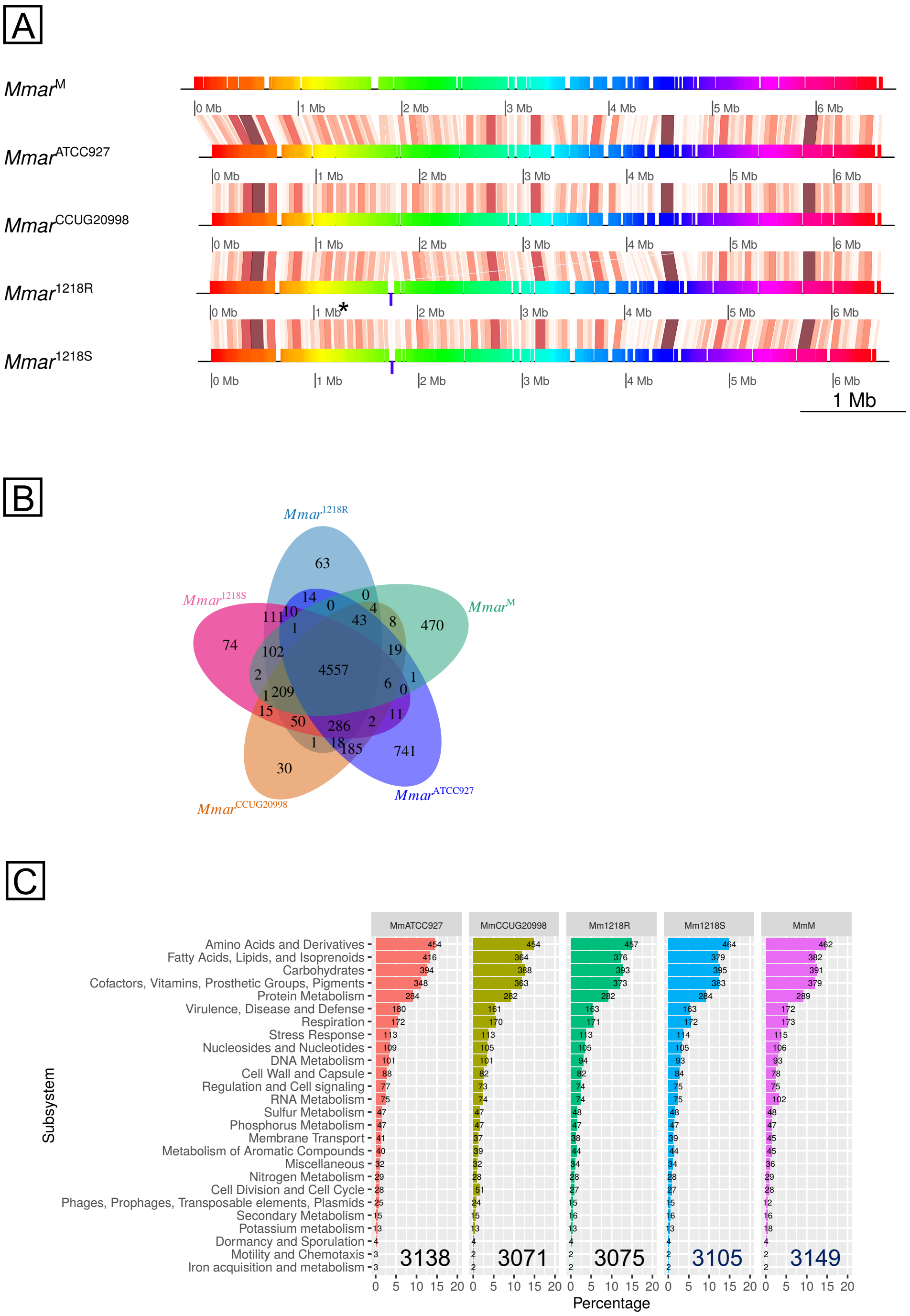

A
MmarM
MmarATCC927
MmarCCUG20998
Mmar1218R
Mmar1218S
0 Mb
1 Mb
2 Mb
3 Mb
4 Mb
5 Mb
6 Mb
1 Mb
B
Mmar1218R
Mmar1218S
MmarM
MmarCCUG20998
MmarATCC927
63
470
74
741
30
4557
C
MmATCC927
MmCCUG20998
Mm1218R
Mm1218S
MmM
Subsystem
Amino Acids and Derivatives
Fatty Acids, Lipids, and Isoprenoids
Carbohydrates
Cofactors, Vitamins, Prosthetic Groups, Pigments
Protein Metabolism
Virulence, Disease and Defense
Respiration
Stress Response
Nucleosides and Nucleotides
DNA Metabolism
Cell Wall and Capsule
Regulation and Cell signaling
RNA Metabolism
Sulfur Metabolism
Phosphorus Metabolism
Membrane Transport
Metabolism of Aromatic Compounds
Miscellaneous
Nitrogen Metabolism
Cell Division and Cell Cycle
Phages, Prophages, Transposable elements, Plasmids
Secondary Metabolism
Potassium metabolism
Dormancy and Sporulation
Motility and Chemotaxis
Iron acquisition and metabolism
3138
3071
3075
3105
3149
Percentage

Figure S2 Analysis of the ESX genes.

(a) Sequence alignment of ESX-1 genes in 1218R and 1218S.

(b) Sequence alignment of ESX-6 genes in 1218R and 1218S.

(c) Gene synteny of the ESX-3, ESX-4 and ESX-5 in 1218R, 1218S, *Mmar*^{ATCC927}, *Mmar*^{CCUG} and *Mmar*^{M} as indicated. Arrows represent genes where blue colours mark genes with known function, grey hypothetical genes.

(d) Sequence alignment of ESX-3 genes in 1218R and 1218S.

(e) Sequence alignment of ESX-4 genes in 1218R and 1218S.

(f) Sequence alignment of ESX-5 genes in 1218R and 1218S.

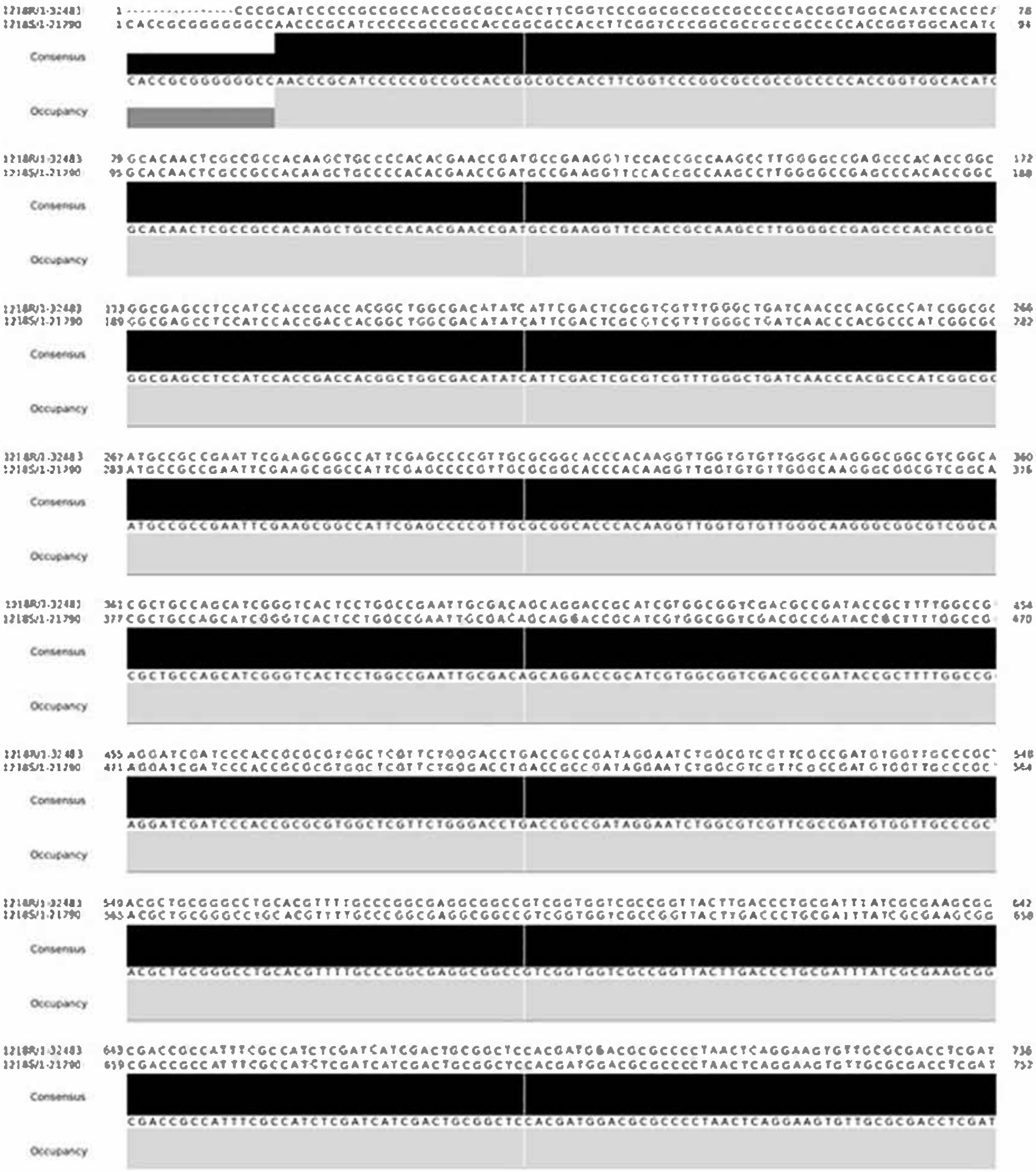

Fig S2A

```
1218R/1-32483   737 GTGGTGTCCTCCCCCTGGGCGGACGGTGCCTCGGCGGCCGCCAAGACAATGGAATGGCTCGCAGACCGCAAACTCAGCGGCTTG  830
1218S/1-21790   753 GTGGTGTCCTCCCCCTGGGCGGACGGTGCCTCGGCGGCCGCCAAGACAATGGAATGGCTCGCAGACCGCAAACTCAGCGGCTTG  846
Consensus           GTGGTGTCCTCCCCCTGGGCGGACGGTGCCTCGGCGGCCGCCAAGACAATGGAATGGCTCGCAGACCGCAAACTCAGCGGCTTG
Occupancy

1218R/1-32483   831 GCGTCGTGGTGCTCAACGATTCGGACGGGCATTCCGACAAGCGCACGCGTTCAGTGCTTGCGCGCGAGTTCGTCGACCATGGAC  924
1218S/1-21790   847 GCGTCGTGGTGCTCAACGATTCGGACGGGCATTCCGACAAGCGCACGCGTTCAGTGCTTGCGCGCGAGTTCGTCGACCATGGAC  940
Consensus           GCGTCGTGGTGCTCAACGATTCGGACGGGCATTCCGACAAGCGCACGCGTTCAGTGCTTGCGCGCGAGTTCGTCGACCATGGAC
Occupancy

1218R/1-32483   925 CGAGGTGCCTTTTGATCCGCATCTGCGCCCCGGCGGTGTTATCGATGTGAGCCATGAGTTGGAGCCGGGAACGCGACTGAAGTTT  1018
1218S/1-21790   941 CGAGGTGCCTTTTGATCCGCATCTGCGCCCCGGCGGTGTTATCGATGTGAGCCATGAGTTGGAGCCGGGAACGCGACTGAAGTTT  1034
Consensus           CGAGGTGCCTTTTGATCCGCATCTGCGCCCCGGCGGTGTTATCGATGTGAGCCATGAGTTGGAGCCGGGAACGCGACTGAAGTTT
Occupancy

1218R/1-32483  1019 GCCGCAACGATTACCGGGCACTTCGCCGCGCGGTCCGCTGCCGACGAAGACCCCCGTCCCACCGAAAACGTAGCGTCTGAGACC  1112
1218S/1-21790  1035 GCCGCAACGATTACCGGGCACTTCGCCGCGCGGTCCGCTGCCGACGAAGACCCCCGTCCCACCGAAAACGTAGCGTCTGAGACC  1128
Consensus           GCCGCAACGATTACCGGGCACTTCGCCGCGCGGTCCGCTGCCGACGAAGACCCCCGTCCCACCGAAAACGTAGCGTCTGAGACC
Occupancy

1218R/1-32483  1113 GGTCCGCTGGATGCTCGAATGCAAAACAGCGCGAGAAATCGTTGAATTTCTCGCGCTGTTTGCCTACGTGGCAATTCACCCCGGC  1206
1218S/1-21790  1129 GGTCCGCTGGATGCTCGAATGCAAAACAGCGCGAGAAATCGTTGAATTTCTCGCGCTGTTTGCCTACGTGGCAATTCACCCCGGC  1222
Consensus           GGTCCGCTGGATGCTCGAATGCAAAACAGCGCGAGAAATCGTTGAATTTCTCGCGCTGTTTGCCTACGTGGCAATTCACCCCGGC
Occupancy

1218R/1-32483  1207 GACCCCTCGCAGCAGCGGCGGACGCGGCCGGGCATCATGCCGATTGGGCGGTGATCCGCTCGCGCGCCGGGAAACCGTTGCGC  1300
1218S/1-21790  1223 GACCCCTCGCAGCAGCGGCGGACGCGGCCGGGCATCATGCCGATTGGGCGGTGATCCGCTCGCGCGCCGGGAAACCGTTGCGC  1316
Consensus           GACCCCTCGCAGCAGCGGCGGACGCGGCCGGGCATCATGCCGATTGGGCGGTGATCCGCTCGCGCGCCGGGAAACCGTTGCGC
Occupancy

1218R/1-32483  1301 CCGAAGTTGGCCCAACGCGAACGCCCGCGCCCGGCCTGCTTCCGGAATCACAACACCCGCCCAAAGTCCCTCCGCACCCGGCGA  1394
1218S/1-21790  1317 CCGAAGTTGGCCCAACGCGAACGCCCGCGCCCGGCCTGCTTCCGGAATCACAACACCCGCCCAAAGTCCCTCCGCACCCGGCGA  1410
Consensus           CCGAAGTTGGCCCAACGCGAACGCCCGCGCCCGGCCTGCTTCCGGAATCACAACACCCGCCCAAAGTCCCTCCGCACCCGGCGA
Occupancy

1218R/1-32483  1395 TCCCGCGCACACGCCCACCGACGCGGGCATGCGCGACACAGCGTCTTGGCCTCCTCGTCGGGCGTTGTCGTCCAACGATCGGGG  1488
1218S/1-21790  1411 TCCCGCGCACACGCCCACCGACGCGGGCATGCGCGACACAGCGTCTTGGCCTCCTCGTCGGGCGTTGTCGTCCAACGATCGGGG  1504
Consensus           TCCCGCGCACACGCCCACCGACGCGGGCATGCGCGACACAGCGTCTTGGCCTCCTCGTCGGGCGTTGTCGTCCAACGATCGGGG
Occupancy

1218R/1-32483  1489 AAACGCCCAGCGGAATCTCGTACAGAGCAGTTGCAGTCATATCTAAGCGTTCCTCCATAAAGCAAAAGCGTTGCAGGTTTGTAAC  1582
1218S/1-21790  1505 AAACGCCCAGCGGAATCTCGTACAGAGCAGTTGCAGTCATATCTAAGCGTTCCTCCATAAAGCAAAAGCGTTGCAGGTTTGTAAC  1598
Consensus           AAACGCCCAGCGGAATCTCGTACAGAGCAGTTGCAGTCATATCTAAGCGTTCCTCCATAAAGCAAAAGCGTTGCAGGTTTGTAAC
Occupancy
```

```
1218R/1-32483  1583 AGGATCGTATAGCACTAACCGTTGCAGTGCAACAGTTCCTGATGCGGGGCTACGCACCCACCTGCTACAACTTCGCTGCGAGCTG  1676
1218S/1-21790  1599 AGGATCGTATAGCACTAACCGTTGCAGTGCAACAGTTCCTGATGCGGGGCTACGCACCCACCTGCTACAACTTCGCTGCGAGCTG  1692
Consensus
                    AGGATCGTATAGCACTAACCGTTGCAGTGCAACAGTTCCTGATGCGGGGCTACGCACCCACCTGCTACAACTTCGCTGCGAGCTG
Occupancy

1218R/1-32483  1677 AAGCATCCAAGCTGGCATAACCAGCGGTAATTACCTGTCAATCGGCTGGGCGACACGGCGCTCCACCAGCCCAGAAGCGCACCCT  1770
1218S/1-21790  1693 AAGCATCCAAGCTGGCATAACCAGCGGTAATTACCTGTCAATCGGCTGGGCGACACGGCGCTCCACCAGCCCAGAAGCGCACCCT  1786
Consensus
                    AAGCATCCAAGCTGGCATAACCAGCGGTAATTACCTGTCAATCGGCTGGGCGACACGGCGCTCCACCAGCCCAGAAGCGCACCCT
Occupancy

1218R/1-32483  1771 AAACTGTTCCACTGCGCAATGCTTCGGGGTAGGATCAAACGTAGCGGTGGTCCGGTTTAACCAGACTACGGTCGCCTCAGAACCC  1864
1218S/1-21790  1787 AAACTGTTCCACTGCGCAATGCTTCGGGGTAGGATCAAACGTAGCGGTGGTCCGGTTTAACCAGACTACGGTCGCCTCAGAACCC  1880
Consensus
                    AAACTGTTCCACTGCGCAATGCTTCGGGGTAGGATCAAACGTAGCGGTGGTCCGGTTTAACCAGACTACGGTCGCCTCAGAACCC
Occupancy

1218R/1-32483  1865 TCATTCAAGAAACGTAGCGAAGGCATACCAATGGAAGGGTGCAACGGTTAGGATGTTGGTCAGCGGAACGCGCGCCTTGCCACCC  1958
1218S/1-21790  1881 TCATTCAAGAAACGTAGCGAAGGCATACCAATGGAAGGGTGCAACGGTTAGGATGTTGGTCAGCGGAACGCGCGCCTTGCCACCC  1974
Consensus
                    TCATTCAAGAAACGTAGCGAAGGCATACCAATGGAAGGGTGCAACGGTTAGGATGTTGGTCAGCGGAACGCGCGCCTTGCCACCC
Occupancy

1218R/1-32483  1959 GCCGCGAGCGAGGAATCGACGTACATGCCCGACGCCGAATCGACTGCTCGGCCGGCCCGTCAACGACCCGTCGGCCGCGGCGCC  2052
1218S/1-21790  1975 GCCGCGAGCGAGGAATCGACGTACATGCCCGACGCCGAATCGACTGCTCGGCCGGCCCGTCAACGACCCGTCGGCCGCGGCGCC  2068
Consensus
                    GCCGCGAGCGAGGAATCGACGTACATGCCCGACGCCGAATCGACTGCTCGGCCGGCCCGTCAACGACCCGTCGGCCGCGGCGCC
Occupancy

1218R/1-32483  2053 CACGACTGGATGATCCCGTGTTATCGCAGACACATGTGCGAGCGGTTTCCGATGGTGGGCAGCGGCGGATCGTCACCAACAGCCC  2146
1218S/1-21790  2069 CACGACTGGATGATCCCGTGTTATCGCAGACACATGTGCGAGCGGTTTCCGATGGTGGGCAGCGGCGGATCGTCACCAACAGCCC  2162
Consensus
                    CACGACTGGATGATCCCGTGTTATCGCAGACACATGTGCGAGCGGTTTCCGATGGTGGGCAGCGGCGGATCGTCACCAACAGCCC
Occupancy

1218R/1-32483  2147 GTTCGTCGACGGAACACGGAAAAGTTCTGTGGCTGTCAACGACAAGACCATTGTCCGGTTCGGCGATCCCACTGGAGGCAAGGCC  2240
1218S/1-21790  2163 GTTCGTCGACGGAACACGGAAAAGTTCTGTGGCTGTCAACGACAAGACCATTGTCCGGTTCGGCGATCCCACTGGAGGCAAGGCC  2256
Consensus
                    GTTCGTCGACGGAACACGGAAAAGTTCTGTGGCTGTCAACGACAAGACCATTGTCCGGTTCGGCGATCCCACTGGAGGCAAGGCC
Occupancy

1218R/1-32483  2241 GAAGTCGTCAGGCCCTCGAATTCGCCTGAAGAAGACAGCCGTGAACAGCGACCGGCCGAGCAATCGGACTCTCAGACCAACGAG  2334
1218S/1-21790  2257 GAAGTCGTCAGGCCCTCGAATTCGCCTGAAGAAGACAGCCGTGAACAGCGACCGGCCGAGCAATCGGACTCTCAGACCAACGAG  2350
Consensus
                    GAAGTCGTCAGGCCCTCGAATTCGCCTGAAGAAGACAGCCGTGAACAGCGACCGGCCGAGCAATCGGACTCTCAGACCAACGAG
Occupancy

1218R/1-32483  2335 GTGTGACTCGCGCCGGGGCCGCCGCCGCGGCACGACGCCGAGAGCTCGACATCAGTCAGCGCAGCCTGGCAGCCGACGGGATCA  2428
1218S/1-21790  2351 GTGTGACTCGCGCCGGGGCCGCCGCCGCGGCACGACGCCGAGAGCTCGACATCAGTCAGCGCAGCCTGGCAGCCGACGGGATCA  2444
Consensus
                    GTGTGACTCGCGCCGGGGCCGCCGCCGCGGCACGACGCCGAGAGCTCGACATCAGTCAGCGCAGCCTGGCAGCCGACGGGATCA
Occupancy
```

1218R/1-32483 2429 CGCACTGATCGCGTTCGAGAAGGGCCGCAGCTGGCCGCGCGAACGCACCCGCGCCAAGCTGGAAGAAGTCCTGCAGTGGCCCG 2522
1218S/1-21790 2445 CGCACTGATCGCGTTCGAGAAGGGCCGCAGCTGGCCGCGCGAACGCACCCGCGCCAAGCTGGAAGAAGTCCTGCAGTGGCCCG 2538

Consensus

CGCACTGATCGCGTTCGAGAAGGGCCGCAGCTGGCCGCGCGAACGCACCCGCGCCAAGCTGGAAGAAGTCCTGCAGTGGCCCG

Occupancy

1218R/1-32483 2523 GCGCGGATACGCCAGGGCGAGTCCGTTGCACCAGAGCCCGCTCCCGAAGCCAGCGTGGAAGCGCCGACCTCCGACGGCCCAGC 2616
1218S/1-21790 2539 GCGCGGATACGCCAGGGCGAGTCCGTTGCACCAGAGCCCGCTCCCGAAGCCAGCGTGGAAGCGCCGACCTCCGACGGCCCAGC 2632

Consensus

GCGCGGATACGCCAGGGCGAGTCCGTTGCACCAGAGCCCGCTCCCGAAGCCAGCGTGGAAGCGCCGACCTCCGACGGCCCAGC

Occupancy

1218R/1-32483 2617 CGCAGGCGGTTGCCGCCGCGGTCGACACCTGCAGCCTCGCAATCGCCGCATTGCCCGCGCCCGAGGAACCTGACTTCACCGAGC 2710
1218S/1-21790 2633 CGCAGGCGGTTGCCGCCGCGGTCGACACCTGCAGCCTCGCAATCGCCGCATTGCCCGCGCCCGAGGAACCTGACTTCACCGAGC 2726

Consensus

CGCAGGCGGTTGCCGCCGCGGTCGACACCTGCAGCCTCGCAATCGCCGCATTGCCCGCGCCCGAGGAACCTGACTTCACCGAGC

Occupancy

1218R/1-32483 2711 GATCCTTGCTGATTTGCGCCAGCTCGAGGGCATCGCCGTCCAGGCGACCCGCATCAGCCGCATCACACCGGAGCTGATCAAGGC 2804
1218S/1-21790 2727 GATCCTTGCTGATTTGCGCCAGCTCGAGGGCATCGCCGTCCAGGCGACCCGCATCAGCCGCATCACACCGGAGCTGATCAAGGC 2820

Consensus

GATCCTTGCTGATTTGCGCCAGCTCGAGGGCATCGCCGTCCAGGCGACCCGCATCAGCCGCATCACACCGGAGCTGATCAAGGC

Occupancy

1218R/1-32483 2805 GTTCGGCGATATCACGACAAATTGATGACGCTCAGCGCAACCGCACCAGGAGCCACACTGGCGCAGCGTTTGTATGCCGCCCGG 2898
1218S/1-21790 2821 GTTCGGCGATATCACGACAAATTGATGACGCTCAGCGCAACCGCACCAGGAGCCACACTGGCGCAGCGTTTGTATGCCGCCCGG 2914

Consensus

GTTCGGCGATATCACGACAAATTGATGACGCTCAGCGCAACCGCACCAGGAGCCACACTGGCGCAGCGTTTGTATGCCGCCCGG

Occupancy

1218R/1-32483 2899 ATCTGTCCACTTCGGAAACCGCGCAAGCCGCCGGAGTGACCGAAGAATTGATCGTCCGCGCGGAAGCCGAGGAAGCTTTGCATG 2992
1218S/1-21790 2915 ATCTGTCCACTTCGGAAACCGCGCAAGCCGCCGGAGTGACCGAAGAATTGATCGTCCGCGCGGAAGCCGAGGAAGCTTTGCATG 3008

Consensus

ATCTGTCCACTTCGGAAACCGCGCAAGCCGCCGGAGTGACCGAAGAATTGATCGTCCGCGCGGAAGCCGAGGAAGCTTTGCATG

Occupancy

1218R/1-32483 2993 CGAGGCTATTGAAGCGCTCATCCGTCAAATCAATTGAGGTTGGTGCCCAGTGGCAACTCACCTTGAATCGCTCGAGTCCGAACGA 3086
1218S/1-21790 3009 CGAGGCTATTGAAGCGCTCATCCGTCAAATCAATTGAGGTTGGTGCCCAGTGGCAACTCACCTTGAATCGCTCGAGTCCGAACGA 3102

Consensus

CGAGGCTATTGAAGCGCTCATCCGTCAAATCAATTGAGGTTGGTGCCCAGTGGCAACTCACCTTGAATCGCTCGAGTCCGAACGA

Occupancy

1218R/1-32483 3087 TTCGCGTTACCCCGGTATTCGAGCATGCGGATCTTGGTGCTGTGCGCGCGAAAATGCCCGCACTACCAAGAAATTCGTGCGTCGA 3180
1218S/1-21790 3103 TTCGCGTTACCCCGGTATTCGAGCATGCGGATCTTGGTGCTGTGCGCGCGAAAATGCCCGCACTACCAAGAAATTCGTGCGTCGA 3196

Consensus

TTCGCGTTACCCCGGTATTCGAGCATGCGGATCTTGGTGCTGTGCGCGCGAAAATGCCCGCACTACCAAGAAATTCGTGCGTCGA

Occupancy

1218R/1-32483 3181 GAGCACTGCTAAGCTCAATGTACAGACCAAAGGTGCACACAAAAAGACAGTGCACGAAACAGCGAGTGTAAGGAATTCCGCGAAT 3274
1218S/1-21790 3197 GAGCACTGCTAAGCTCAATGTACAGACCAAAGGTGCACACAAAAAGACAGTGCACGAAACAGCGAGTGTAAGGAATTCCGCGAAT 3290

Consensus

GAGCACTGCTAAGCTCAATGTACAGACCAAAGGTGCACACAAAAAGACAGTGCACGAAACAGCGAGTGTAAGGAATTCCGCGAAT

Occupancy

```
1218R/1-32483  3275 GGGAAGCGGTCTTTGCAAGACAACAAGCAATTTTATTTGGGGTCAGTTGCTTTTACTTGGAGAAGGCATCCCCGACCCGGGCGAT  3368
1218S/1-21790  3291 GGGAAGCGGTCTTTGCAAGACAACAAGCAATTTTATTTGGGGTCAGTTGCTTTTACTTGGAGAAGGCATCCCCGACCCGGGCGAT  3384
Consensus
                    GGGAAGCGGTCTTTGCAAGACAACAAGCAATTTTATTTGGGGTCAGTTGCTTTTACTTGGAGAAGGCATCCCCGACCCGGGCGAT
Occupancy

1218R/1-32483  3369 ACCGGTTCGACGCTCTTCAAAGGAATCGCCGACAAGATGGGTCTGGCAATTCCGGGCACCAACTGGCTCGGCCAGGCGGCGGAC  3462
1218S/1-21790  3385 ACCGGTTCGACGCTCTTCAAAGGAATCGCCGACAAGATGGGTCTGGCAATTCCGGGCACCAACTGGCTCGGCCAGGCGGCGGAC  3478
Consensus
                    ACCGGTTCGACGCTCTTCAAAGGAATCGCCGACAAGATGGGTCTGGCAATTCCGGGCACCAACTGGCTCGGCCAGGCGGCGGAC
Occupancy

1218R/1-32483  3463 ACCAGAACATCGCTCAAGAACTTCGCGCGAAGGTGATGGGTGACGTCGACTATCTGACCGGCAACCTGATTTCGAATCAGGCCGA  3556
1218S/1-21790  3479 ACCAGAACATCGCTCAAGAACTTCGCGCGAAGGTGATGGGTGACGTCGACTATCTGACCGGCAACCTGATTTCGAATCAGGCCGA  3572
Consensus
                    ACCAGAACATCGCTCAAGAACTTCGCGCGAAGGTGATGGGTGACGTCGACTATCTGACCGGCAACCTGATTTCGAATCAGGCCGA
Occupancy

1218R/1-32483  3557 GAACACCCGCGACGTGCTGCGCGCGATGAAGAAGATGATCGACGGCGTCTACAAGGTCTGCAAAGGCCTCGAAAAGGTGCCGAT  3650
1218S/1-21790  3573 GAACACCCGCGACGTGCTGCGCGCGATGAAGAAGATGATCGACGGCGTCTACAAGGTCTGCAAAGGCCTCGAAAAGGTGCCGAT  3666
Consensus
                    GAACACCCGCGACGTGCTGCGCGCGATGAAGAAGATGATCGACGGCGTCTACAAGGTCTGCAAAGGCCTCGAAAAGGTGCCGAT
Occupancy

1218R/1-32483  3651 TTGTGGTCGTGGGAGCTCGCCCTGCCGATGTCGGGCATTGCGATGGCCACCGTCGGCGGCGCGCTGCTTTACCTGACCATCATGA  3744
1218S/1-21790  3667 TTGTGGTCGTGGGAGCTCGCCCTGCCGATGTCGGGCATTGCGATGGCCACCGTCGGCGGCGCGCTGCTTTACCTGACCATCATGA  3760
Consensus
                    TTGTGGTCGTGGGAGCTCGCCCTGCCGATGTCGGGCATTGCGATGGCCACCGTCGGCGGCGCGCTGCTTTACCTGACCATCATGA
Occupancy

1218R/1-32483  3745 ACCTGACCAACCTGAAGGGCCTGCTCGGCAGGTTGGTCGAAATGTTGGCCAGCCTGCCATCGCTGATAGGCGGCCTGCTCCCGAA  3838
1218S/1-21790  3761 ACCTGACCAACCTGAAGGGCCTGCTCGGCAGGTTGGTCGAAATGTTGGCCAGCCTGCCATCGCTGATAGGCGGCCTGCTCCCGAA  3854
Consensus
                    ACCTGACCAACCTGAAGGGCCTGCTCGGCAGGTTGGTCGAAATGTTGGCCAGCCTGCCATCGCTGATAGGCGGCCTGCTCCCGAA
Occupancy

1218R/1-32483  3839 CATCATCGACGGCCTGTGGCCGCCCAAGTTGCCCGACCTTCCCATCCCGGGTCTGCCGAACATCCCGGGCCTGCCCGAGTTCACC  3932
1218S/1-21790  3855 CATCATCGACGGCCTGTGGCCGCCCAAGTTGCCCGACCTTCCCATCCCGGGTCTGCCGAACATCCCGGGCCTGCCCGAGTTCACC  3948
Consensus
                    CATCATCGACGGCCTGTGGCCGCCCAAGTTGCCCGACCTTCCCATCCCGGGTCTGCCGAACATCCCGGGCCTGCCCGAGTTCACC
Occupancy

1218R/1-32483  3933 AAGATCGACATTCCGAACTGGAACTTGCCGATCCCGGGGCTCCCCGGTTTCGAGTTCCCGCCGACCTCGGGTATCCCCGGTATCG  4026
1218S/1-21790  3949 AAGATCGACATTCCGAACTGGAACTTGCCGATCCCGGGGCTCCCCGGTTTCGAGTTCCCGCCGACCTCGGGTATCCCCGGTATCG  4042
Consensus
                    AAGATCGACATTCCGAACTGGAACTTGCCGATCCCGGGGCTCCCCGGTTTCGAGTTCCCGCCGACCTCGGGTATCCCCGGTATCG
Occupancy

1218R/1-32483  4027 TCCCCAACATCCCGGGCTTGCCCAGCTTCCCCAGCCTTCCTGGGTTGCCGAGCATCCCGGACCTGTTCCCCGGCTTGCCCGGCCT  4120
1218S/1-21790  4043 TCCCCAACATCCCGGGCTTGCCCAGCTTCCCCAGCCTTCCTGGGTTGCCGAGCATCCCGGACCTGTTCCCCGGCTTGCCCGGCCT  4136
Consensus
                    TCCCCAACATCCCGGGCTTGCCCAGCTTCCCCAGCCTTCCTGGGTTGCCGAGCATCCCGGACCTGTTCCCCGGCTTGCCCGGCCT
Occupancy
```

```
1218R/1-32483  4121 GTTCTCCGGGATCGGCAATTGGGGCAAGTTGCCCACCTGGACAGATTTGGCGGCCCTACCCGACTTCTTGGGTGGCTTTGCCGGC 4214
1218S/1-21790  4137 GTTCTCCGGGATCGGCAATTGGGGCAAGTTGCCCACCTGGACAGATTTGGCGGCCCTACCCGACTTCTTGGGTGGCTTTGCCGGC 4230
Consensus
                    GTTCTCCGGGATCGGCAATTGGGGCAAGTTGCCCACCTGGACAGATTTGGCGGCCCTACCCGACTTCTTGGGTGGCTTTGCCGGC
Occupancy

1218R/1-32483  4215 CTGAGCTTCTCCAACCTGCTCGGCTTCGCCCAATTGCCCAACGTCAGTTCGCTGACCGCGACGATGGGCCAGCTGCAACACCTGG 4308
1218S/1-21790  4231 CTGAGCTTCTCCAACCTGCTCGGCTTCGCCCAATTGCCCAACGTCAGTTCGCTGACCGCGACGATGGGCCAGCTGCAACACCTGG 4324
Consensus
                    CTGAGCTTCTCCAACCTGCTCGGCTTCGCCCAATTGCCCAACGTCAGTTCGCTGACCGCGACGATGGGCCAGCTGCAACACCTGG
Occupancy

1218R/1-32483  4309 CTGGCGGACCTGGCCAACTGGGCAGCATGGCGGGCCAGCAGGCCAGCATGATCTCGTCGCAGGCTTCCCAAGGCGGTCAACAGG 4402
1218S/1-21790  4325 CTGGCGGACCTGGCCAACTGGGCAGCATGGCGGGCCAGCAGGCCAGCATGATCTCGTCGCAGGCTTCCCAAGGCGGTCAACAGG 4418
Consensus
                    CTGGCGGACCTGGCCAACTGGGCAGCATGGCGGGCCAGCAGGCCAGCATGATCTCGTCGCAGGCTTCCCAAGGCGGTCAACAGG
Occupancy

1218R/1-32483  4403 GAGCGACAAGAAGGAAGACGACGAAGACGGTGCGGCCGCGGGCAGCGCCGGTGCGGAACGTGCTCCCATCGACGGGGGAAACA 4496
1218S/1-21790  4419 GAGCGACAAGAAGGAAGACGACGAAGACGGTGCGGCCGCGGGCAGCGCCGGTGCGGAACGTGCTCCCATCGACGGGGGAAACA 4512
Consensus
                    GAGCGACAAGAAGGAAGACGACGAAGACGGTGCGGCCGCGGGCAGCGCCGGTGCGGAACGTGCTCCCATCGACGGGGGAAACA
Occupancy

1218R/1-32483  4497 GGCAACGAGGGGACCCTCCTCTAGTCCGGGTAACCGGTGGCGGCTCACTGGCCTACCAAAGCCATAGCGAGCCAAAGCCATAGC 4590
1218S/1-21790  4513 GGCAACGAGGGGACCCTCCTCTAGTCCGGGTAACCGGTGGCGGCTCACTGGCCTACCAAAGCCATAGCGAGCCAAAGCCATAGC 4606
Consensus
                    GGCAACGAGGGGACCCTCCTCTAGTCCGGGTAACCGGTGGCGGCTCACTGGCCTACCAAAGCCATAGCGAGCCAAAGCCATAGC
Occupancy

1218R/1-32483  4591 TTAGACGTAGAGGAAAGGTCTACCCCCATGACAGGACTACTGAACGTCGTACCTTCATTCTTGAAGGTGCTGGCGGGCATGCACA 4684
1218S/1-21790  4607 TTAGACGTAGAGGAAAGGTCTACCCCCATGACAGGACTACTGAACGTCGTACCTTCATTCTTGAAGGTGCTGGCGGGCATGCACA 4695
Consensus
                    TTAGACGTAGAGGAAAGGTCTACCCCCATGACAGGACTACTGAACGTCGTACCTTCATTCTTGAAGGTGCTGGCGGGCATGCACA
Occupancy

1218R/1-32483  4685 TCGGCGAACTCAAATCGGCGACCAACGTCGTGAGTGGAATCGGCTCGCGGGTGCAGGTGACCCACGGCTCATTCACCTCGAATTT 4778
1218S/1-21790       ------------------------------------------------------------------------
Consensus
                    TCGGCGAACTCAAATCGGCGACCAACGTCGTGAGTGGAATCGGCTCGCGGGTGCAGGTGACCCACGGCTCATTCACCTCGAATTT
Occupancy

1218R/1-32483  4779 GCTGGTCGAGTTCGAAACCACCCGCAACAGCGCCGGGACAGGCCTGCAGGGCGTCACGGGCAAGTTGGCCAACAATCTGATCTC 4872
1218S/1-21790       ------------------------------------------------------------------------
Consensus
                    GCTGGTCGAGTTCGAAACCACCCGCAACAGCGCCGGGACAGGCCTGCAGGGCGTCACGGGCAAGTTGGCCAACAATCTGATCTC
Occupancy

1218R/1-32483  4873 GCCTATCTGAACTCCGACGAAGGGCTCGCTGGCATCATCGACAAGATTTTTGGCTGATATGGCCGGTCCGCTCGCTACCGGTCGC 4966
1218S/1-21790       ------------------------------------------------------------------------
Consensus
                    GCCTATCTGAACTCCGACGAAGGGCTCGCTGGCATCATCGACAAGATTTTTGGCTGATATGGCCGGTCCGCTCGCTACCGGTCGC
Occupancy
```

1218R/1-32483 4967 GGTGACGATGTCGTCGGAGTCGAGGTAACCATCGACGGCATGCTGGTGATCGCGGACCGGTTACACCTGGTCGATTTCCCTGTCA 5060
1218S/1-21790 --

Consensus
GGTGACGATGTCGTCGGAGTCGAGGTAACCATCGACGGCATGCTGGTGATCGCGGACCGGTTACACCTGGTCGATTTCCCTGTCA

Occupancy

1218R/1-32483 5061 TCCGGCCGAACATCCCGCAAGAGGATCTGCGAGAGATCGTCTGGGACCAGGTGGCGCGCGACCTTACTGCGCAGGGCGTGTTGG 5154
1218S/1-21790 --

Consensus
TCCGGCCGAACATCCCGCAAGAGGATCTGCGAGAGATCGTCTGGGACCAGGTGGCGCGCGACCTTACTGCGCAGGGCGTGTTGG

Occupancy

1218R/1-32483 5155 CCAACCGCATCCGGCGGTTGCGGCGATGGTCGACACGCTCAGCAGGGCGGACCGCACCTTGGAAGGCCGCTGGTGGCGCCGCGA 5248
1218S/1-21790 --

Consensus
CCAACCGCATCCGGCGGTTGCGGCGATGGTCGACACGCTCAGCAGGGCGGACCGCACCTTGGAAGGCCGCTGGTGGCGCCGCGA

Occupancy

1218R/1-32483 5249 GTGATGGTGCGGTTCGTGGTATGCCGCAAGGGCGAAAGACATGTCATCGCAGCTCGCGATGGCGACATGTTGGTACTGCAGCTG 5342
1218S/1-21790 --

Consensus
GTGATGGTGCGGTTCGTGGTATGCCGCAAGGGCGAAAGACATGTCATCGCAGCTCGCGATGGCGACATGTTGGTACTGCAGCTG

Occupancy

1218R/1-32483 5343 GGGTCGGCCTGGCAGGCATGGTGACTGCCGTGCTGGGCACCGCGGAACCCGCCAATGTCGAACCGCTGACCGGCATTGCCAGCC 5436
1218S/1-21790 --

Consensus
GGGTCGGCCTGGCAGGCATGGTGACTGCCGTGCTGGGCACCGCGGAACCCGCCAATGTCGAACCGCTGACCGGCATTGCCAGCC

Occupancy

1218R/1-32483 5437 GTGCACCAACGCCGCACAGCTGACCCGATATGGGCTCACGCCGACCACCGCCCGCCTCTACACCGAGATCGTCACCAACCCGAAC 5530
1218S/1-21790 --

Consensus
GTGCACCAACGCCGCACAGCTGACCCGATATGGGCTCACGCCGACCACCGCCCGCCTCTACACCGAGATCGTCACCAACCCGAAC

Occupancy

1218R/1-32483 5531 GAAATCGTCGCCAGCGAACGCCATCCGGGCGGCACTACCACCCACACCAAGGCCGCCGCGGGGGTCCTGGATTCAGCACATGGC 5624
1218S/1-21790 --

Consensus
GAAATCGTCGCCAGCGAACGCCATCCGGGCGGCACTACCACCCACACCAAGGCCGCCGCGGGGGTCCTGGATTCAGCACATGGC

Occupancy

1218R/1-32483 5625 CGCTTCCCCGTCAAGTCGGCGGGGAACTGTACGGCAGCTTCCTCCCCGGTACCGCGCAGAATCTCCAGCGGGCGCTTGACAGCC 5718
1218S/1-21790 --

Consensus
CGCTTCCCCGTCAAGTCGGCGGGGAACTGTACGGCAGCTTCCTCCCCGGTACCGCGCAGAATCTCCAGCGGGCGCTTGACAGCC

Occupancy

1218R/1-32483 5719 GCTTCCGTCAGGCTCGTGGTTGGATCGCGCCGACGCCACCACCCGAGGTTGACTTAATCCTCTCCGTTACGAGTTCAGAAAGGGA 5812
1218S/1-21790 --

Consensus
GCTTCCGTCAGGCTCGTGGTTGGATCGCGCCGACGCCACCACCCGAGGTTGACTTAATCCTCTCCGTTACGAGTTCAGAAAGGGA

Occupancy

1218R/1-32483 5813 GGACCTGCCCGGGAACGACGACTACAACCAAGATCTCGGCGCCCTAGATTTCTCCGGCGGCGGTACGTCTGAGGACTCTGGACT 5906
1218S/1-21790 --

Consensus

GGACCTGCCCGGGAACGACGACTACAACCAAGATCTCGGCGCCCTAGATTTCTCCGGCGGCGGTACGTCTGAGGACTCTGGACT

Occupancy

1218R/1-32483 5907 GACGAGTACGCGCCCCCCGAGCCCCAGGAAACCGAGCAAGCCGGAGCCGACCTGGATGCACTTCACGGGCTGACCGAGAAGGA 6000
1218S/1-21790 --

Consensus

GACGAGTACGCGCCCCCCGAGCCCCAGGAAACCGAGCAAGCCGGAGCCGACCTGGATGCACTTCACGGGCTGACCGAGAAGGA

Occupancy

1218R/1-32483 6001 ACATCGCGATGTTCACCGTGACCAACCCGCAAGGGAGCGTGTCGGTCACGACCATGATGGGTGGCATCGTCCAGCGAGTTACGG 6094
1218S/1-21790 --

Consensus

ACATCGCGATGTTCACCGTGACCAACCCGCAAGGGAGCGTGTCGGTCACGACCATGATGGGTGGCATCGTCCAGCGAGTTACGG

Occupancy

1218R/1-32483 6095 GGCTGCGAACATGTCGGAATCCGCTCTGGCCGAAGAGATCTTTGTCATCGCTGATCTGGCCCGCCAAAAGGCGCGCGCCGCACA 6188
1218S/1-21790 --

Consensus

GGCTGCGAACATGTCGGAATCCGCTCTGGCCGAAGAGATCTTTGTCATCGCTGATCTGGCCCGCCAAAAGGCGCGCGCCGCACA

Occupancy

6189 ATGGTGGAAGCCATGGCCAGCGAACTGAGCGACGAGACCGAAGAAGAAGGTGCGCTGTTGCGCGAGTTCGTCGGTATGACACTC 6282

ATGGTGGAAGCCATGGCCAGCGAACTGAGCGACGAGACCGAAGAAGAAGGTGCGCTGTTGCGCGAGTTCGTCGGTATGACACTC

6283 CGCCGGAAGAAGCAGAAGCGGCCGAAGCCGAGGTATTCGCCACCCGCTACGAGGTCGACTACACCTCTCGTTACAACGAACGGT 6376

CGCCGGAAGAAGCAGAAGCGGCCGAAGCCGAGGTATTCGCCACCCGCTACGAGGTCGACTACACCTCTCGTTACAACGAACGGT

6377 TGATCGCCTGGCCGGTTTGTTCGAAAGTGCCGTCAGCATGCTTCCGCTGTCGGAGTCAAGGTCCATGGACCTATTCACCGAGATC 6470

TGATCGCCTGGCCGGTTTGTTCGAAAGTGCCGTCAGCATGCTTCCGCTGTCGGAGTCAAGGTCCATGGACCTATTCACCGAGATC

6471 GACGAATCAGCGTGCGATGCTTGGGTCGGCCGGATCCGGTGTGGCGATGTCGACCGGGTGACTTTGTTCCGTGCCTGGTACTCG 6564

GACGAATCAGCGTGCGATGCTTGGGTCGGCCGGATCCGGTGTGGCGATGTCGACCGGGTGACTTTGTTCCGTGCCTGGTACTCG

6565 TCGGTCAGCTGGCTGGCACGGCGCAGATCTCGATGAGCACCCTCAACGCAAGGGTCCCCATAGGTGGTTTGTACGGAGACATCA 6658

TCGGTCAGCTGGCTGGCACGGCGCAGATCTCGATGAGCACCCTCAACGCAAGGGTCCCCATAGGTGGTTTGTACGGAGACATCA

6659 TACCTCGCCCCTGGCCATCACCATGGGCTTCGCCGCATCCGAAGCGGCGCAGGGCAACTACGCGGACGCGATGGAGGCCATCGA 6752

TACCTCGCCCCTGGCCATCACCATGGGCTTCGCCGCATCCGAAGCGGCGCAGGGCAACTACGCGGACGCGATGGAGGCCATCGA

6753 GTCACCGGTTCGGAGCATCTGGTGTCGTGGCTCAAGGCGGTCATCTTCGGCGCGGCCGAGCGCTGGACCGACGTTATCGATGAA 6846

GTCACCGGTTCGGAGCATCTGGTGTCGTGGCTCAAGGCGGTCATCTTCGGCGCGGCCGAGCGCTGGACCGACGTTATCGATGAA

6847 CCGGGAAGTGGCCGGACAAGTTCTTGGCGGGGGCCGCCAGCGTCGCCCATGGGGTTGCGGCGGCCAGCCTTGGCCTGTTCACCC 6940

CCGGGAAGTGGCCGGACAAGTTCTTGGCGGGGGCCGCCAGCGTCGCCCATGGGGTTGCGGCGGCCAGCCTTGGCCTGTTCACCC

6941 CAGACTGACCGAAGCCAATGACTCACCGGCCGGCGAAGCCTGCGCGCAGGCCATCGCGTGGTATCTGGCCATGGCGCGGCGCG 7034

CAGACTGACCGAAGCCAATGACTCACCGGCCGGCGAAGCCTGCGCGCAGGCCATCGCGTGGTATCTGGCCATGGCGCGGCGCG

7035 GAGGAAGCCGCGGTGGCACTGCTGGAGTGGTTGCAGACCACGCATCCGGCTCCGAAAGTCTCTGCTGCGTTGAAGGATCCGTCC 7128

GAGGAAGCCGCGGTGGCACTGCTGGAGTGGTTGCAGACCACGCATCCGGCTCCGAAAGTCTCTGCTGCGTTGAAGGATCCGTCC

7129 AGACAACCAACGCCGAGCAGATTGCGTCCCGCTCGGATCCATGGGATCCGACCAGCGTCGTGACCGACAATTCCGGTCGCGAAA 7222

AGACAACCAACGCCGAGCAGATTGCGTCCCGCTCGGATCCATGGGATCCGACCAGCGTCGTGACCGACAATTCCGGTCGCGAAA

7223 CGAAGCCCAAGAAGAACTCGACCGCCAAATCGGATTGTCCCGGGTAAAAAGCCAGCTCGAGCGGTACCGCGCGGCAACCATGAT 7316

CGAAGCCCAAGAAGAACTCGACCGCCAAATCGGATTGTCCCGGGTAAAAAGCCAGCTCGAGCGGTACCGCGCGGCAACCATGAT

7317 CGCGAAGCTAAAGGCATGAAAGTTGCACAGCCCAGCAAGCACATGATCTTTACCGGGCCCCCTGGCACCGGGAAGACCACGATC 7410

CGCGAAGCTAAAGGCATGAAAGTTGCACAGCCCAGCAAGCACATGATCTTTACCGGGCCCCCTGGCACCGGGAAGACCACGATC

7411 TCGCCAACATGCTGGCCGGGCTCGGCGTCATCACTGAACCCAAGCTGGTCGAGACGTCGCGTAAAGACTTCGTTGCCGAGTACG 7504

TCGCCAACATGCTGGCCGGGCTCGGCGTCATCACTGAACCCAAGCTGGTCGAGACGTCGCGTAAAGACTTCGTTGCCGAGTACG

7505 AGCAGTCAAGACCGCCAAGACGATCGATCAGGCTCTAGGTGGCGTGCTGTTCATCGACGAGGCCTATGCGCTCGTGCAGGAGCG 7598

AGCAGTCAAGACCGCCAAGACGATCGATCAGGCTCTAGGTGGCGTGCTGTTCATCGACGAGGCCTATGCGCTCGTGCAGGAGCG

7599 ACCGACCCGTTCGGCCAGGAAGCGATGGATACTCTGCTAGCCCGGATGGAGAACGACCGCGATCGCTTGGTGGTCATCATCGCC 7692

ACCGACCCGTTCGGCCAGGAAGCGATGGATACTCTGCTAGCCCGGATGGAGAACGACCGCGATCGCTTGGTGGTCATCATCGCC

7693 CCGACATCGATCGGCTGCTGGAAACCAACGAGGGGCTACGCTCGCGATTTGCCACCCGTATCGAATTCGACACCTACAGCCCGG 7786

CCGACATCGATCGGCTGCTGGAAACCAACGAGGGGCTACGCTCGCGATTTGCCACCCGTATCGAATTCGACACCTACAGCCCGG

7787 CGAGATCGCGAAAGTCATTGCGGCTGGCAATGACTCGACGCTGAGCACGGCTGCCGCGGATGAACTCCTGCAGGCGGCCAAAAC 7880

CGAGATCGCGAAAGTCATTGCGGCTGGCAATGACTCGACGCTGAGCACGGCTGCCGCGGATGAACTCCTGCAGGCGGCCAAAAC

7881 CGCACGTTGCGCGGCCGTCCCGCTCTCGACATCGCCGGCAACGGCCGATATGCGCGACAATTGGTTGAGGCCTCTGAGCAGTAC 7974

CGCACGTTGCGCGGCCGTCCCGCTCTCGACATCGCCGGCAACGGCCGATATGCGCGACAATTGGTTGAGGCCTCTGAGCAGTAC

7975 GGCTGGCACAGGGCCTCGACATCGAGGCCCTCGATGTGGACAGACTGCAAGAGATCGACGGCGCGGACATGGCCGAGGCAATCC 8068

GGCTGGCACAGGGCCTCGACATCGAGGCCCTCGATGTGGACAGACTGCAAGAGATCGACGGCGCGGACATGGCCGAGGCAATCC

8069 TGCACACCTCAATATGAGAGAGTGAAACATGGGGCTTCGCCTGACCACCAAGGTTCAGGTAAGCGGCTGGCGCTTCCTGCTTCGC 8162

TGCACACCTCAATATGAGAGAGTGAAACATGGGGCTTCGCCTGACCACCAAGGTTCAGGTAAGCGGCTGGCGCTTCCTGCTTCGC

8163 CATGCCATCGTGCGGCGCGACACCCGCATGTTCGATGATCCGCTGCAGTTCTACAGCCGCTCGATAGCGCTGGGCATCGTTGTCG 8256

CATGCCATCGTGCGGCGCGACACCCGCATGTTCGATGATCCGCTGCAGTTCTACAGCCGCTCGATAGCGCTGGGCATCGTTGTCG

8257 TCCTGGCCGGTGCCGGCCTGCTGGCCTACTTCAAACCAGCTGGAAAACTCGGTGGCAGCAACCTGCTGACCGACCGCGCGACCA 8350

TCCTGGCCGGTGCCGGCCTGCTGGCCTACTTCAAACCAGCTGGAAAACTCGGTGGCAGCAACCTGCTGACCGACCGCGCGACCA

8351 TGTACTGCTGTCCGGGCAGTTGCACCCTGTCTACAACCTGACCTCGGCGCGCCTCGTGTTGGGCAGCCCTGCCACCCCCGTCACC 8444

TGTACTGCTGTCCGGGCAGTTGCACCCTGTCTACAACCTGACCTCGGCGCGCCTCGTGTTGGGCAGCCCTGCCACCCCCGTCACC

8445 TCCGAATTGAGCCAGTTGCCCTTGGGCCAAACCATCGGAATCCCCGGCGCCCCCTACGCCACCCCGGTTTCCGGGGACACTACTT 8538

TCCGAATTGAGCCAGTTGCCCTTGGGCCAAACCATCGGAATCCCCGGCGCCCCCTACGCCACCCCGGTTTCCGGGGACACTACTT

8539 CCCTTTGCGACACCGTCAGCCGGGCGGGTACCGCTTCCGCCTCGGTCGAGACATCGCTGCTCGTGATGCCGCTGCGGATCGATG 8632

CCCTTTGCGACACCGTCAGCCGGGCGGGTACCGCTTCCGCCTCGGTCGAGACATCGCTGCTCGTGATGCCGCTGCGGATCGATG

8633 TCCGATCGAGCCCAACGAGGCGATGCTGGCGGACTATCACGGCCAGACCTGGATCGTCACTTCAAAGGGACGCCACTCGATCGA 8726

TCCGATCGAGCCCAACGAGGCGATGCTGGCGGACTATCACGGCCAGACCTGGATCGTCACTTCAAAGGGACGCCACTCGATCGA

8727 CGTGCGCTCACATCGGCGGTGGGCATCCCCATCACCGCCCAGACGGTCCCCATTTCCGAGGGAATGTTTAATGCGCTTCCGGCCA 8820

CGTGCGCTCACATCGGCGGTGGGCATCCCCATCACCGCCCAGACGGTCCCCATTTCCGAGGGAATGTTTAATGCGCTTCCGGCCA

8821 GGCAATTGCCACCCATCCCCGCCGCCGGAGAGCCAAACACCCTCGGGCTTCCGGAAGATTTGGTAATCGGATCGGTGTTTCAAAT 8914

GGCAATTGCCACCCATCCCCGCCGCCGGAGAGCCAAACACCCTCGGGCTTCCGGAAGATTTGGTAATCGGATCGGTGTTTCAAAT

8915 CAAGGGACCGCAATATTACGTAGTGCTGACCGACGGCATCGCCGCGGTAAATGGCACCACTGCCGCGGCACTGCGCGCCACTCA 9008

CAAGGGACCGCAATATTACGTAGTGCTGACCGACGGCATCGCCGCGGTAAATGGCACCACTGCCGCGGCACTGCGCGCCACTCA

9009 CTGGTGGCGCCGCCCGCGGTGGTGCCCAGCCTGGTCGTCAGGATCCCCGAACGGGTTTACGCATCACCGCTGCCCGACGAGACC 9102

CTGGTGGCGCCGCCCGCGGTGGTGCCCAGCCTGGTCGTCAGGATCCCCGAACGGGTTTACGCATCACCGCTGCCCGACGAGACC

9103 TGTCCCGGCCCGACGACCCAGTCTTGTGCTGGGAATGGGAACGTAGCGCTGGGGACCAGGCCCCCAACACAACGGTTCTCACCG 9196

TGTCCCGGCCCGACGACCCAGTCTTGTGCTGGGAATGGGAACGTAGCGCTGGGGACCAGGCCCCCAACACAACGGTTCTCACCG

9197 GCCCATCCCGCCCTCGGCCATGAAGACCGGCCTCAAACAGATCCAGGGCAGGTCAACCGTCTATATCGACGGCGGGAAATTTATT 9290

GCCCATCCCGCCCTCGGCCATGAAGACCGGCCTCAAACAGATCCAGGGCAGGTCAACCGTCTATATCGACGGCGGGAAATTTATT

9291 TCACCCGATCCCCGATACGGCGAATCGATGTACTACATCGACCCGGAAGGGGTGCGCTACGGGGTGCCCGACGCCGACGCGGCC 9384

TCACCCGATCCCCGATACGGCGAATCGATGTACTACATCGACCCGGAAGGGGTGCGCTACGGGGTGCCCGACGCCGACGCGGCC

9385 GCCTGGGCATGCCGAAGACCGCACCGTGGGAGATCGTTCGCCTCCTGGTGGACGGTCCGGTGTTATCAAAAGACGCCGCTCTGC[illegible] 9478

GCCTGGGCATGCCGAAGACCGCACCGTGGGAGATCGTTCGCCTCCTGGTGGACGGTCCGGTGTTATCAAAAGACGCCGCTCTGC[illegible]

9479 AACGTTGCCCTCCGACCCCAATCCCCGAAAAGTTCCAGCTGGGACACCCGGAGCACCTCAATGACGACAAAGAAATTCACCCCAA 9572

AACGTTGCCCTCCGACCCCAATCCCCGAAAAGTTCCAGCTGGGACACCCGGAGCACCTCAATGACGACAAAGAAATTCACCCCAA

9573 GTGGCCCCCGACTCACCCCGGGCGAGATCAGCCTCACCCCACCCGAGGATCTCGGCATCGACATCCCGCCGTCGGGCGTGCAGA 9666

GTGGCCCCCGACTCACCCCGGGCGAGATCAGCCTCACCCCACCCGAGGATCTCGGCATCGACATCCCGCCGTCGGGCGTGCAGA

9667 CTACGTCATGGGCGGCGCGATGCTGGGCATGATCGTGATCATGGTGGCCGGCGGCACCAGACAGCTATCGCCATACATGCTGAT[illegible] 9760

CTACGTCATGGGCGGCGCGATGCTGGGCATGATCGTGATCATGGTGGCCGGCGGCACCAGACAGCTATCGCCATACATGCTGAT[illegible]

9761 ATGATGATCGTGATGATGGTGGGCACACTCGCCGGGGGCAGCGGCGGCGGCAGCAAGAAGGTGCCCGAGATCAATGCCGACCG[illegible] 9854

ATGATGATCGTGATGATGGTGGGCACACTCGCCGGGGGCAGCGGCGGCGGCAGCAAGAAGGTGCCCGAGATCAATGCCGACCG[illegible]

9855 TGCGGTATCTCGCCGGCCTGCGCGGCCGCGTAACGACGTCGGCCACCTCCCAGGTTTCGTTCTTCGGCTACCACGCGCCCCATCC 9948

TGCGGTATCTCGCCGGCCTGCGCGGCCGCGTAACGACGTCGGCCACCTCCCAGGTTTCGTTCTTCGGCTACCACGCGCCCCATCC

9949 TCTCTCCATCGTCGGCACCCAGCGGCAGTGGTCAAGGCCGGCCAACAGCGACTTCTATGCGGCAGCCCGCATCGGGATCGGCGA 10042

TCTCTCCATCGTCGGCACCCAGCGGCAGTGGTCAAGGCCGGCCAACAGCGACTTCTATGCGGCAGCCCGCATCGGGATCGGCGA

10043 GTGGACCGGCTATTGAAGCCGGCGGTCGGCGGCGAGCTGGCGGCCAGCACCGCGGCCCCCCAGCCCTATCTCGAGCCGGTAAG[illegible] 10136

GTGGACCGGCTATTGAAGCCGGCGGTCGGCGGCGAGCTGGCGGCCAGCACCGCGGCCCCCCAGCCCTATCTCGAGCCGGTAAG[illegible]

10137 TGGTCAAGTTCCTGCGTACCCACGGGCTGATCCACGACTGTCCGAAACTCGTGCAGCTGCGCAGTTTCCCAACGATCGCGATCGG 10230

TGGTCAAGTTCCTGCGTACCCACGGGCTGATCCACGACTGTCCGAAACTCGTGCAGCTGCGCAGTTTCCCAACGATCGCGATCGG

10231 ACCGGGAGCCGATCGACTGCTGACCGCGATGATCTGCCACCTGGCGGTCTTCCATCCGCCCGACCTGTTGCAGATCCGCGTCCTC 10324

ACCGGGAGCCGATCGACTGCTGACCGCGATGATCTGCCACCTGGCGGTCTTCCATCCGCCCGACCTGTTGCAGATCCGCGTCCTC

10325 CCCGAGGATCCCGACTGGTCCTGGTTGAAATGGCTGCCACACGTCCAGCACCAGACCGAAACCGACGGGGCCGGGCCGGTCCGG 10418

CCCGAGGATCCCGACTGGTCCTGGTTGAAATGGCTGCCACACGTCCAGCACCAGACCGAAACCGACGGGGCCGGGCCGGTCCGG

10419 CGCGCCCGGACGGCCTCGCCGACCTGGCCGCCCGGGGACCCCACGCGCCCGACACCCTCCCCACCGGTCCCTACGTCGTGGTCC 10512

CGCGCCCGGACGGCCTCGCCGACCTGGCCGCCCGGGGACCCCACGCGCCCGACACCCTCCCCACCGGTCCCTACGTCGTGGTCC

10513 CGGCGGCAAGGCGGGCTTCCCGCCAGACGGCAGGGCCGGGGTAACGGTAATCACGCTGGGCAACCATCGCGGGTCTGCCTACC[illegible] 10606

CGGCGGCAAGGCGGGCTTCCCGCCAGACGGCAGGGCCGGGGTAACGGTAATCACGCTGGGCAACCATCGCGGGTCTGCCTACC[illegible]

10607 GCCGAGAACGGCACCGCTGACGACCGGCTGCCTGGGCAGCAGTTCCGGCTGGTGACCTCGGCCGCCGACGGCATGACGCCGCA[illegible] 10700

GCCGAGAACGGCACCGCTGACGACCGGCTGCCTGGGCAGCAGTTCCGGCTGGTGACCTCGGCCGCCGACGGCATGACGCCGCA[illegible]

10701 GCCTCGCCCGCAAGCTGGCCGGATGGTCGATCACCGGAACCATCCTCGACAAGACCCAACGCATCCAGAAGAAGGTAGCGACCG 10794

GCCTCGCCCGCAAGCTGGCCGGATGGTCGATCACCGGAACCATCCTCGACAAGACCCAACGCATCCAGAAGAAGGTAGCGACCG

10795 GTTGGTCAACGCCAAGAGCGTTGAGGACATCACCCCGGGCCGTTGGCGGATGTACACCGACACTGATCGAGACCGGCTCAAGAT[illegible] 10888

GTTGGTCAACGCCAAGAGCGTTGAGGACATCACCCCGGGCCGTTGGCGGATGTACACCGACACTGATCGAGACCGGCTCAAGAT[illegible]

10889 CACGAACTCAAGACCGGCAATGTCATGTACCTGGACATCAAGGAGGGTGCGGAGTTCGGCGGCGGGCCGCACGGCATGCTCATC 10982

CACGAACTCAAGACCGGCAATGTCATGTACCTGGACATCAAGGAGGGTGCGGAGTTCGGCGGCGGGCCGCACGGCATGCTCATC

10983 GTTCAGGCAAGTCCGAGTTCCTGCGCACCATGATCTTGTCGTTGGTGGCGATGACCCACCCCGATCAGGTGAACCTGCTGCTCAC 11076

GTTCAGGCAAGTCCGAGTTCCTGCGCACCATGATCTTGTCGTTGGTGGCGATGACCCACCCCGATCAGGTGAACCTGCTGCTCAC

11077 GGGTGGCTCGACATTTCTTGGGATGGAGAAGCTCCCGCACACCGCGGCTGTCATCACCAACATGGCCGAGGAAGCCGAACTCGT 11170

GGGTGGCTCGACATTTCTTGGGATGGAGAAGCTCCCGCACACCGCGGCTGTCATCACCAACATGGCCGAGGAAGCCGAACTCGT

11171 GGTGAGGTGCTGACCGGCGAACTGGACCGCCGCCAGTCGATCCTGCGTCAGGCGGGGATGAAGGTCGGCGCGGCCGGCGCGCT 11264

GGTGAGGTGCTGACCGGCGAACTGGACCGCCGCCAGTCGATCCTGCGTCAGGCGGGGATGAAGGTCGGCGCGGCCGGCGCGCT

11265 CCGAGTACGAGAAGTACCGCGAACGCGGCGCCGACCTTCCGCCGCTGCCGACACTTTTCGTTGTGGTGGACGAGTTTGCCGAAC 11358

CCGAGTACGAGAAGTACCGCGAACGCGGCGCCGACCTTCCGCCGCTGCCGACACTTTTCGTTGTGGTGGACGAGTTTGCCGAAC

11359 TCACCCGGATTTCATCGGCCTGTTCGACCGAATTTGCCGAGTCGGGCGATCATTGCGGGTGCACCTGCTACTGGCCACCCAGTCA 11452

TCACCCGGATTTCATCGGCCTGTTCGACCGAATTTGCCGAGTCGGGCGATCATTGCGGGTGCACCTGCTACTGGCCACCCAGTCA

11453 GGCGGTGTCCGCATCGACAAGCTCGAACCCAACCTGACGTATCGCATCGCGTTGCGTACCACGAGCTCTCATGAATCCAAAGCGG 11546

GGCGGTGTCCGCATCGACAAGCTCGAACCCAACCTGACGTATCGCATCGCGTTGCGTACCACGAGCTCTCATGAATCCAAAGCGG

11547 CGCCGGAAGCCCAGTACATCACCAACAAGGAAAGCGGTGTCGGCTTTCTCCGGGTCGGTATGGAGGACCCCATCAAGTTCAGCA 11640

CGCCGGAAGCCCAGTACATCACCAACAAGGAAAGCGGTGTCGGCTTTCTCCGGGTCGGTATGGAGGACCCCATCAAGTTCAGCA

11641 CAGTGGGCCATACGTTCCGCCGGCGACCGCTGAAACCAACGGCGATGGCAGCGGACCCAGCACCCAATTCGCCAAGCGAGCCTT 11734

CAGTGGGCCATACGTTCCGCCGGCGACCGCTGAAACCAACGGCGATGGCAGCGGACCCAGCACCCAATTCGCCAAGCGAGCCTT

11735 GAGTTCACCGCTGCTCCGGTCCTCGAGGAAGCGCTGACACCATGATCCACGCCGGCGGTGCTGCAGCACGAAGCAACGAAAGGG 11828

GAGTTCACCGCTGCTCCGGTCCTCGAGGAAGCGCTGACACCATGATCCACGCCGGCGGTGCTGCAGCACGAAGCAACGAAAGGG

11829 ATGACTGCCGAACCTGAAGTACGGACGTTGCGTGAGGTCATCCTTGACCAGCTCAGTACCGTCGAGTCACGCGCCTACAAGATGT 11922

ATGACTGCCGAACCTGAAGTACGGACGTTGCGTGAGGTCATCCTTGACCAGCTCAGTACCGTCGAGTCACGCGCCTACAAGATGT

11923 CACTGGTTGATCCGACCCCGCTCGACGAGCTGATCGCCCGCGATCGGCGACAACCCCTTCGCTTCGCGCTGGGAATCATGGACG[illegible] 12016

CACTGGTTGATCCGACCCCGCTCGACGAGCTGATCGCCCGCGATCGGCGACAACCCCTTCGCTTCGCGCTGGGAATCATGGACG[illegible]

12017 TCACCTGCAGGACGTCTGGGGCGTCGATGTATCGGGCGCCGGCGGAAACATCGGTATTGGCGGTGCACCCCAGACCGGCAAGTC 12110

TCACCTGCAGGACGTCTGGGGCGTCGATGTATCGGGCGCCGGCGGAAACATCGGTATTGGCGGTGCACCCCAGACCGGCAAGTC

12111 CAGACGATGGTCATGTCGGCCGCCGCCACGCACTCGCCGCGCAAGGTCCAGTTCTATTGCATCGACCTCGGTGGTGGCGGCCTG[illegible] 12204

CAGACGATGGTCATGTCGGCCGCCGCCACGCACTCGCCGCGCAAGGTCCAGTTCTATTGCATCGACCTCGGTGGTGGCGGCCTG[illegible]

12205 AGAACCTGCCCCACGTGGGCGGAGTCGCGGGCCGGTCCGAACCCGACAAAGTCCACCGGGTGGTCGCGGAGATGCAGGCCGTG[illegible] 12298

AGAACCTGCCCCACGTGGGCGGAGTCGCGGGCCGGTCCGAACCCGACAAAGTCCACCGGGTGGTCGCGGAGATGCAGGCCGTG[illegible]

12299 GGAAGCCACCTTCAAGGAACACCGGGTCGGCTCGATCGCGATGTATCGCCAGCTGCGTGACGACCCCAACCAGCCTGTCGCCTC[illegible] 12392

GGAAGCCACCTTCAAGGAACACCGGGTCGGCTCGATCGCGATGTATCGCCAGCTGCGTGACGACCCCAACCAGCCTGTCGCCTC[illegible]

12393 GGCGATGTATTCCTGATCATCGATGGATGGCCCGCCTTCGTCAGTGAGTTTCCCGACCTCGAAGGGCAGGTCCAAGATCTGGCCG 12486

GGCGATGTATTCCTGATCATCGATGGATGGCCCGCCTTCGTCAGTGAGTTTCCCGACCTCGAAGGGCAGGTCCAAGATCTGGCCG

12487 TGTCCTTTGGTGTGCACACGATCCTGTCCACACCGCGTTGGACGGAGTTGAAATCACGTGTCCGCGACTACCTGGGCACCAAGAT 12580

TGTCCTTTGGTGTGCACACGATCCTGTCCACACCGCGTTGGACGGAGTTGAAATCACGTGTCCGCGACTACCTGGGCACCAAGAT

12581 GCTCGGCGACGTCAACGAAACCCAGATCGACCGCATCACCCGGGAGATCCCGGCGAACCGCCCGGGTCGGGCGGTGTCGATGG[illegible] 12674

GCTCGGCGACGTCAACGAAACCCAGATCGACCGCATCACCCGGGAGATCCCGGCGAACCGCCCGGGTCGGGCGGTGTCGATGG[illegible]

12675 CTGATGATCGGGGTGCCCCGGCTCGACGGCGTACACAGCGCCGACAATCTGGTGGAGGCGATTACGGCGGGCGTAGCGCAGATC 12768

CTGATGATCGGGGTGCCCCGGCTCGACGGCGTACACAGCGCCGACAATCTGGTGGAGGCGATTACGGCGGGCGTAGCGCAGATC

12769 ACACGGACCAGGCACCTCCGGTGCGAACCCTGCCGGAACGCATCCACCTCCACGAACTGGACCCCAACCCTCCCGGGCCCGAAT 12862

ACACGGACCAGGCACCTCCGGTGCGAACCCTGCCGGAACGCATCCACCTCCACGAACTGGACCCCAACCCTCCCGGGCCCGAAT

12863 CACCCGCTGGGAGATTCCGATCGGATTGCGCGAATCCGACATGGAAGTGGCCTACAGCCATATGCACTCCAACCCGCACCTGCTC 12956

CACCCGCTGGGAGATTCCGATCGGATTGCGCGAATCCGACATGGAAGTGGCCTACAGCCATATGCACTCCAACCCGCACCTGCTC

12957 GCCGCCAAGTCGGGCAAGACGACCATTGCCCACGCGATCGCACGCGCCATCTGCGCTCGAAACAGTCCCGACCAGGTGCGGTTC 13050

GCCGCCAAGTCGGGCAAGACGACCATTGCCCACGCGATCGCACGCGCCATCTGCGCTCGAAACAGTCCCGACCAGGTGCGGTTC

13051 ACTATCGCTCTGGACTCCTCGATGCGGTGCCCGACACACACCTGCTCTCGGCCGGCGCCATCAACCGCAACAGCGCGACGCTGG 13144

ACTATCGCTCTGGACTCCTCGATGCGGTGCCCGACACACACCTGCTCTCGGCCGGCGCCATCAACCGCAACAGCGCGACGCTGG

13145 CAAAGCCCTGGCCGCCAACTTGAAGAATCGGCTGCCTCCAGCCGATCTCACGACGTCTCAACTGCGCTCGCGTTCGTGGTGGAGC 13238

CAAAGCCCTGGCCGCCAACTTGAAGAATCGGCTGCCTCCAGCCGATCTCACGACGTCTCAACTGCGCTCGCGTTCGTGGTGGAGC

13239 GTTGTGCTGCTCGTCGACGACTGGCACATGATTGTCGGTGCTGCCGGGGGCATGCCCCCGATGGCGCCACTTGCGCCCTTATTGC 13332

GTTGTGCTGCTCGTCGACGACTGGCACATGATTGTCGGTGCTGCCGGGGGCATGCCCCCGATGGCGCCACTTGCGCCCTTATTGC

13333 CCGATATCGGCTTGCACATCATTGTGACCTGCCAGATGAGCCAGGCCTACAAGGCGACCATGGACAAGTTCGTCGGTGCCGCATT 13426

CCGATATCGGCTTGCACATCATTGTGACCTGCCAGATGAGCCAGGCCTACAAGGCGACCATGGACAAGTTCGTCGGTGCCGCATT

13427 CGCTCCAACAATGTTCCTTTCCGGCGACAAGCAGGAATTCCCCTCCAGCGAGTTCAAGGTCAAGCGGCGCCCCCCCGGCCAGGCC 13520

CGCTCCAACAATGTTCCTTTCCGGCGACAAGCAGGAATTCCCCTCCAGCGAGTTCAAGGTCAAGCGGCGCCCCCCCGGCCAGGCC

13521 TCGCCCGATGGCAAAGAGGTCATCCAGGCCCCTTACATCGAGCCGCCAGAAGAAGTGTTCGCAGCACCCCCAAGCCCCGGTTAG 13614

TCGCCCGATGGCAAAGAGGTCATCCAGGCCCCTTACATCGAGCCGCCAGAAGAAGTGTTCGCAGCACCCCCAAGCCCCGGTTAG

13615 TTGCCAGCGACGCATCACCCGAGCTGATGGAAATCAAGCCCAGGAGGCGATCACCGGCAGGCCGTTTCGGCGACAACCAAATGA 13708

TTGCCAGCGACGCATCACCCGAGCTGATGGAAATCAAGCCCAGGAGGCGATCACCGGCAGGCCGTTTCGGCGACAACCAAATGA

13709 GAAGCGCAAATAAGGGAGAAGAAGCAGGCAAATGGAACAAAAGTCACACGGCGCGGCGATCGCCGACATCGGCACACTATTGAG 13802

GAAGCGCAAATAAGGGAGAAGAAGCAGGCAAATGGAACAAAAGTCACACGGCGCGGCGATCGCCGACATCGGCACACTATTGAG

13803 CGCATTGGTGTGACCTCCGATGCGGCAGCGTTGGCGTCGGTGACCGGGGTGGTTCCAGCTGGAGCAGACGAGGTGTCGACGCAA 13896

CGCATTGGTGTGACCTCCGATGCGGCAGCGTTGGCGTCGGTGACCGGGGTGGTTCCAGCTGGAGCAGACGAGGTGTCGACGCAA

13897 CCTTCGCCGCCGAGGGCGCCCAGTTGCTGGCTTCGAGCTCGGCGGCTCAGTGGGAGATCCACCGAGCCGGCGAATCGCCCCACC 13990

CCTTCGCCGCCGAGGGCGCCCAGTTGCTGGCTTCGAGCTCGGCGGCTCAGTGGGAGATCCACCGAGCCGGCGAATCGCCCCACC

13991 TAACCCTGCAGAAGTCAGCGACGGCGCAGCCAGCGTCATCGTGTAGAAAATCAGTCACCAGACACGGCGCAACAAGAAGGAGTG 14084

TAACCCTGCAGAAGTCAGCGACGGCGCAGCCAGCGTCATCGTGTAGAAAATCAGTCACCAGACACGGCGCAACAAGAAGGAGTG

14085 TGTGGCACGCAATGCCACCGGAGCTGAATACCGCTCGCCTGATGGCCGGAGCGGGCCCGGCCCCGATGCTGGCCGCGGCCGCCC 14178

TGTGGCACGCAATGCCACCGGAGCTGAATACCGCTCGCCTGATGGCCGGAGCGGGCCCGGCCCCGATGCTGGCCGCGGCCGCCC

14179 TCTGGCAGCCGCCTTGGACGCTCAGGCCGTCGAATTGACCGCGCGCTTGAACTCGCTCGGCGAAGCGTGGACCGGAGGCGGCAG 14272

TCTGGCAGCCGCCTTGGACGCTCAGGCCGTCGAATTGACCGCGCGCTTGAACTCGCTCGGCGAAGCGTGGACCGGAGGCGGCAG

14273 CTGGCGGCCGCCCTGCCGATGGTGACCTGGTTGCAGACCGCCTCGACCCAGGCCAAGACGCGCGGCCTACAAGCCGGTGCGCAC 14366

CTGGCGGCCGCCCTGCCGATGGTGACCTGGTTGCAGACCGCCTCGACCCAGGCCAAGACGCGCGGCCTACAAGCCGGTGCGCAC

14367 ACATGCAGGCCATGGCCACAACGCCTTCGCTACCCGAGATCTTTGCCAACCACATCACCAACGTGATCCTCAACGCGACCAACTT 14460

ACATGCAGGCCATGGCCACAACGCCTTCGCTACCCGAGATCTTTGCCAACCACATCACCAACGTGATCCTCAACGCGACCAACTT

14461 CAACACGGTTCCCATCGCCTTCAACGAGATGGATTACTTCGTCCGCATGTGGAACCAGGCGGCGCTGGCGATGGATGTCTACCAG 14554

CAACACGGTTCCCATCGCCTTCAACGAGATGGATTACTTCGTCCGCATGTGGAACCAGGCGGCGCTGGCGATGGATGTCTACCAG

14555 ACGGCCAACACGCTGTTCGAACAGCTCGAGCCGATGACGTCGATCCTCGATCCCGCCACTGCACAGAGCATGCCGACTTCGTCGA 14648

ACGGCCAACACGCTGTTCGAACAGCTCGAGCCGATGACGTCGATCCTCGATCCCGCCACTGCACAGAGCATGCCGACTTCGTCGA

14649 TGGACATGGCGTCACAGGTCACCGGCATACCGAGCAGTGAGCTTCAGCAGACCGCCACGCAGGTCGCCGAGGCGAGTGGCCCCA 14742

TGGACATGGCGTCACAGGTCACCGGCATACCGAGCAGTGAGCTTCAGCAGACCGCCACGCAGGTCGCCGAGGCGAGTGGCCCCA

14743 GGCACAACCGGCGCAGCAGATGACGTCGGCGTTCAGCAACACCGGCAGCTCGGGCAACGGCGCGGACGAAGAAGGCTTCCGGA 14836

GGCACAACCGGCGCAGCAGATGACGTCGGCGTTCAGCAACACCGGCAGCTCGGGCAACGGCGCGGACGAAGAAGGCTTCCGGA

14837 GGGGCCGGCGCGCTGTCCAATCACCCGCTGGCGGGTGGGTCAGGCCCGAGCACCGGCGCGGGCCTGCTGCGCGGTGAATCGCT 14930

GGGGCCGGCGCGCTGTCCAATCACCCGCTGGCGGGTGGGTCAGGCCCGAGCACCGGCGCGGGCCTGCTGCGCGGTGAATCGCT

14931 GCGGGACCCTAACCCGCACGCCACTGATCAGCGAACTCGTCGAAAAGCCGATGGGTCCGTCAGTGCAGCCGGCTGCGGCTGCCG 15024

GCGGGACCCTAACCCGCACGCCACTGATCAGCGAACTCGTCGAAAAGCCGATGGGTCCGTCAGTGCAGCCGGCTGCGGCTGCCG

15025 AAGCAGTGGCGCCGCCCCGGTGGGCGCCGGCGGAATGGGGGCAGGAGCTGGCGCCGGTGCTGGCGGTTCGTCGCGGCCAGGCA 15118

AAGCAGTGGCGCCGCCCCGGTGGGCGCCGGCGGAATGGGGGCAGGAGCTGGCGCCGGTGCTGGCGGTTCGTCGCGGCCAGGCA

15119 GCGACGCTGACCCAGGAGCGGGACGAGGCCGACGAAGACGACTGGGATGACGAGGACGACTGGTGAGCGTCGTCAACACGAAC 15212

GCGACGCTGACCCAGGAGCGGGACGAGGCCGACGAAGACGACTGGGATGACGAGGACGACTGGTGAGCGTCGTCAACACGAAC

15213 CCACCCGGGCCGGAAGACTTGCCAACAATTTGGCGAGGAAAGAAAGAGAGAAAGTAGTCCAGCATGGCAGAGATGAAGACCGAT 15306

```
      CCACCCGGGCCGGAAGACTTGCCAACAATTTGGCGAGGAAAGAAAGAGAGAAAGTAGTCCAGCATGGCAGAGATGAAGACCGAT

15307 TCGCGCAGGAGGCAGGTAATTTCGAGCGGATCTCCGGTGACCTGAAGACCCAGATCGACCAGGTTGAGTCGACCGCCGGTTCGC 15400
 4696 ---------------------------------------------------------GGTTCGCTGCAGGCCCA                    4712
      TCGCGCAGGAGGCAGGTAATTTCGAGCGGATCTCCGGTGACCTGAAGACCCAGATCGACCAGGTTGAGTCGACCGCCGGTTCGC

15401 GTGGCGCGGTGCGGCTGGTACCGCCGCTCAGGCTGCGGTGGTCCGTTTCCAGGAAGCCGCCAACAAGCAGAAGGCCGAACTCGA 15494
 4713 GTGGCGCGGTGCGGCTGGTACCGCCGCTCAGGCTGCGGTGGTCCGTTTCCAGGAAGCCGCCAACAAGCAGAAGGCCGAACTCGA 4806
      GTGGCGCGGTGCGGCTGGTACCGCCGCTCAGGCTGCGGTGGTCCGTTTCCAGGAAGCCGCCAACAAGCAGAAGGCCGAACTCGA

15495 ACGAACATCCGTCAGGCCGGTGTCCAGTACTCCCGGGCCGACGACGAGCAGCAGCAGGCGCTGTCCTCGCAAATGGGCTTCTGA 15588
 4807 ACGAACATCCGTCAGGCCGGTGTCCAGTACTCCCGGGCCGACGACGAGCAGCAGCAGGCGCTGTCCTCGCAAATGGGCTTCTGA 4900
      ACGAACATCCGTCAGGCCGGTGTCCAGTACTCCCGGGCCGACGACGAGCAGCAGCAGGCGCTGTCCTCGCAAATGGGCTTCTGA

15589 ACGACAAAGAAACGGAGCAATACGACATGACAGAACAGCAGTGGAATTTCGCCGGCATTGAGGCCGCATCCAGCGCAATTCAGG 15682
 4901 ACGACAAAGAAACGGAGCAATACGACATGACAGAACAGCAGTGGAATTTCGCCGGCATTGAGGCCGCATCCAGCGCAATTCAGG 4994
      ACGACAAAGAAACGGAGCAATACGACATGACAGAACAGCAGTGGAATTTCGCCGGCATTGAGGCCGCATCCAGCGCAATTCAGG

15683 CAGCATTCATTCCCTTCTGGATGAGGGCAAGCAGTCGCTGACCAAGCTGTCCGCAGCCTGGGGCGGTAGCGGTTCGGAGGCCTA 15776
 4995 CAGCATTCATTCCCTTCTGGATGAGGGCAAGCAGTCGCTGACCAAGCTGTCCGCAGCCTGGGGCGGTAGCGGTTCGGAGGCCTA 5088
      CAGCATTCATTCCCTTCTGGATGAGGGCAAGCAGTCGCTGACCAAGCTGTCCGCAGCCTGGGGCGGTAGCGGTTCGGAGGCCTA

15777 CAGCAGAAGTGGGACTCCACCGCGCAGGAGCTCAACAACTCGCTGCAGAACCTGGCTCGCACCATCAGCGAGGCCGGTCAGGCA 15870
 5089 CAGCAGAAGTGGGACTCCACCGCGCAGGAGCTCAACAACTCGCTGCAGAACCTGGCTCGCACCATCAGCGAGGCCGGTCAGGCA 5182
      CAGCAGAAGTGGGACTCCACCGCGCAGGAGCTCAACAACTCGCTGCAGAACCTGGCTCGCACCATCAGCGAGGCCGGTCAGGCA

15871 CCGAGGGCAACGTCACGGGGATGTTTGCTTAATCCCCCCTCTCGTTCGCGTAGAATACCGAAGCACGAGATCGGGGCGAGTTTCA 15964
 5183 CCGAGGGCAACGTCACGGGGATGTTTGCTTAATCCCCCCTCTCGTTCGCGTAGAATACCGAAGCACGAGATCGGGGCGAGTTTCA 5276
      CCGAGGGCAACGTCACGGGGATGTTTGCTTAATCCCCCCTCTCGTTCGCGTAGAATACCGAAGCACGAGATCGGGGCGAGTTTCA
```

```
15965 AATCTCGCCCCTTCTCGTGCTTCGTTTTATTGGCGAACTTCTGAGAGGTTCCTATGCCGGCGGACTACGACGAGCTATTTCGGCCC 16058
5277  AATCTCGCCCCTTCTCGTGCTTCGTTTTATTGGCGAACTTCTGAGAGGTTCCTATGCCGGCGGACTACGACGAGCTATTTCGGCCC 5370
      AATCTCGCCCCTTCTCGTGCTTCGTTTTATTGGCGAACTTCTGAGAGGTTCCTATGCCGGCGGACTACGACGAGCTATTTCGGCCC

16059 TTCCGGACCTCCAGATGATGAAACTGGGCAAACATTCTTTGATCCTGGTACCGCGTATCCGCCGCCCGTAAAACCCAACGGCGAC 16152
5371  TTCCGGACCTCCAGATGATGAAACTGGGCAAACATTCTTTGATCCTGGTACCGCGTATCCGCCGCCCGTAAAACCCAACGGCGAC 5464
      TTCCGGACCTCCAGATGATGAAACTGGGCAAACATTCTTTGATCCTGGTACCGCGTATCCGCCGCCCGTAAAACCCAACGGCGAC

16153 GCGCCTAAGGACTGGCCGCGCGCATTTCCGCCGGCCGAAGAAGCGTCGCCGTCAGACTCGGCAGAGCCGACAGCTGGCCCCGCC 16246
5465  GCGCCTAAGGACTGGCCGCGCGCATTTCCGCCGGCCGAAGAAGCGTCGCCGTCAGACTCGGCAGAGCCGACAGCTGGCCCCGCC 5558
      GCGCCTAAGGACTGGCCGCGCGCATTTCCGCCGGCCGAAGAAGCGTCGCCGTCAGACTCGGCAGAGCCGACAGCTGGCCCCGCC

16247 TGCCCCCCATGCCCATCGGCGGGCCTGCGCCGACACCCCCAGAACCACCACCGG-CCCCCCGGAGCTACCACCGGCACCGGCTC 16339
5559  TGCCCCCCATGCCCATCGGCGGGCCTGCGCCGACACCCCCAGAACCACCACCGGCCCCCCCGGAGCTACCACCGGCACCGGCTC 5652
      TGCCCCCCATGCCCATCGGCGGGCCTGCGCCGACACCCCCAGAACCACCACCGGCCCCCCCGGAGCTACCACCGGCACCGGCTC

16340 ACCTCCGCAAGCGCCGACAGCCGAGGCTGAACCTCCGGACGAGGCAATACCCGTTAGCGGGCCCCCGCCTGGCAGCAAGTCGCC 16433
5653  ACCTCCGCAAGCGCCGACAGCCGAGGCTGAACCTCCGGACGAGGCAATACCCGTTAGCGGGCCCCCGCCTGGCAGCAAGTCGCC 5746
      ACCTCCGCAAGCGCCGACAGCCGAGGCTGAACCTCCGGACGAGGCAATACCCGTTAGCGGGCCCCCGCCTGGCAGCAAGTCGCC

16434 ATGCCTATCGGTGGGCCGCCACCGGCATCTCCGGAGCTACCTGCGGCTCCACCGGAACCGACGGCGCCAGCAGTGCCACCGCAG 16527
5747  ATGCCTATCGGTGGGCCGCCACCGGCATCTCCGGAGCTACCTGCGGCTCCACCGGAACCGACGGCGCCAGCAGTGCCACCGCAG 5840
      ATGCCTATCGGTGGGCCGCCACCGGCATCTCCGGAGCTACCTGCGGCTCCACCGGAACCGACGGCGCCAGCAGTGCCACCGCAG

16528 CTGCTGCACAACCGCCGCAGGCCGTCGAGGAACAGGCCCACGCAACCGCCGAACCCCCAGCGGCACCAAAACCGCCGCGTCCCC 16621
5841  CTGCTGCACAACCGCCGCAGGCCGTCGAGGAACAGGCCCACGCAACCGCCGAACCCCCAGCGGCACCAAAACCGCCGCGTCCCC 5934
      CTGCTGCACAACCGCCGCAGGCCGTCGAGGAACAGGCCCACGCAACCGCCGAACCCCCAGCGGCACCAAAACCGCCGCGTCCCC

16622 CGGCGGGCCCCCACCCACACCCCCGGCCGCACCGGAACCACAACAGGGCGCGCCGCAACCACACCAGGACATAGAAGAAGAACC 16715
5935  CGGCGGGCCCCCACCCACACCCCCGGCCGCACCGGAACCACAACAGGGCGCGCCGCAACCACACCAGGACATAGAAGAAGAACC 6028
      CGGCGGGCCCCCACCCACACCCCCGGCCGCACCGGAACCACAACAGGGCGCGCCGCAACCACACCAGGACATAGAAGAAGAACC

16716 GCCGCCGAAGCCCCAGCGGCACCACCGAAATCACCGCTACCGCCCATGCCGGTCAGCGGACCGCCACCGGAGCCGCCCGAACTG 16809
6029  GCCGCCGAAGCCCCAGCGGCACCACCGAAATCACCGCTACCGCCCATGCCGGTCAGCGGACCGCCACCGGAGCCGCCCGAACTG 6122
      GCCGCCGAAGCCCCAGCGGCACCACCGAAATCACCGCTACCGCCCATGCCGGTCAGCGGACCGCCACCGGAGCCGCCCGAACTG
```

16810 CACCGGAACCGACGGCCGCAGCAGTGCCACCGCAGCCGCCGGACGCTGCTGCACAACCGCCGCAGGCCGGCGAGGAACGGGCC 16903
6123 CACCGGAACCGACGGCCGCAGCAGTGCCACCGCAGCCGCCGGACGCTGCTGCACAACCGCCGCAGGCCGGCGAGGAACGGGCC 6216
CACCGGAACCGACGGCCGCAGCAGTGCCACCGCAGCCGCCGGACGCTGCTGCACAACCGCCGCAGGCCGGCGAGGAACGGGCC

16904 CGAACCCCCAGCGGCACCAAAACCGCCGCGTCCCCCATGCCCATCGGCGGGCCCCCACCCACACCGGAACCACAACAGGGCGC 16997
6217 CGAACCCCCAGCGGCACCAAAACCGCCGCGTCCCCCATGCCCATCGGCGGGCCCCCACCCACACCGGAACCACAACAGGGCGC 6310
CGAACCCCCAGCGGCACCAAAACCGCCGCGTCCCCCATGCCCATCGGCGGGCCCCCACCCACACCGGAACCACAACAGGGCGC

16998 CACCAGGACATAGAAGAAGAACCGAAACCGGCGGCCGCCGAAGCCCCAGCGGCACCACCGAAATCACCGCTACCGCCCATGCCG 17091
6311 CACCAGGACATAGAAGAAGAACCGAAACCGGCGGCCGCCGAAGCCCCAGCGGCACCACCGAAATCACCGCTACCGCCCATGCCG 6404
CACCAGGACATAGAAGAAGAACCGAAACCGGCGGCCGCCGAAGCCCCAGCGGCACCACCGAAATCACCGCTACCGCCCATGCCG

17092 CGGCACCCACCCGGCCCGAGCCACCGCTACCACCGGGCCCCCCGCGTCGCCGCGCTCAACCGCCAGCGGCGCCACCGAACCAGC 17185
6405 CGGCACCCACCCGGCCCGAGCCACCGCTACCACCGGGCCCCCCGCGTCGCCGCGCTCAACCGCCAGCGGCGCCACCGAACCAGC 6498
CGGCACCCACCCGGCCCGAGCCACCGCTACCACCGGGCCCCCCGCGTCGCCGCGCTCAACCGCCAGCGGCGCCACCGAACCAGC

17186 GAAGCCCATCAGCGGACACCCGCCGCCCCCACCGAGGCCAGCGGCGTTCGCACCCCCAGCACGCGGCGCCACGCCAAACCGGCA 17279
6499 GAAGCCCATCAGCGGACACCCGCCGCCCCCACCGAGGCCAGCGGCGTTCGCACCCCCAGCACGCGGCGCCACGCCAAACCGGCA 6592
GAAGCCCATCAGCGGACACCCGCCGCCCCCACCGAGGCCAGCGGCGTTCGCACCCCCAGCACGCGGCGCCACGCCAAACCGGCA

17280 GAACCTCCGCCACCGCGCCGGGTCCGGATCGGTGGCCCGCCTCAGCCTCCAGGGCCACCAGAGGCTGAATCCGAGGCTCCTCGG 17373
6593 GAACCTCCGCCACCGCGCCGGGTCCGGATCGGTGGCCCGCCTCAGCCTCCAGGGCCACCAGAGGCTGAATCCGAGGCTCCTCGG 6686
GAACCTCCGCCACCGCGCCGGGTCCGGATCGGTGGCCCGCCTCAGCCTCCAGGGCCACCAGAGGCTGAATCCGAGGCTCCTCGG

17374 ATGCCCGACGGACGCATCGCTATCGGCCCGAGCCCGAAACCGATGACCTCGACACGGCGGTACGACCGCTTCCGACACGCGAGC 17467
6687 ATGCCCGACGGACGCATCGCTATCGGCCCGAGCCCGAAACCGATGACCTCGACACGGCGGTACGACCGCTTCCGACACGCGAGC 6780
ATGCCCGACGGACGCATCGCTATCGGCCCGAGCCCGAAACCGATGACCTCGACACGGCGGTACGACCGCTTCCGACACGCGAGC

17468 AAACGGACCCGCGGCGGACGAAAGCTCAACCGCGTCCTTCGCGTGGCTGCAGCAGAGCCAACCGACGCTCGATCGGCCGTCCGG 17561
6781 AAACGGACCCGCGGCGGACGAAAGCTCAACCGCGTCCTTCGCGTGGCTGCAGCAGAGCCAACCGACGCTCGATCGGCCGTCCGG 6874
AAACGGACCCGCGGCGGACGAAAGCTCAACCGCGTCCTTCGCGTGGCTGCAGCAGAGCCAACCGACGCTCGATCGGCCGTCCGG

17562 GCCGCCCCTGGGGCCCCCGTCGAATCCGCGCCGGGTCGCGCTGATGGTCGTAGGGCCAGGCGGCGCGCCGAATCCCGGACTTCC 17655
6875 GCCGCCCCTGGGGCCCCCGTCGAATCCGCGCCGGGTCGCGCTGATGGTCGTAGGGCCAGGCGGCGCGCCGAATCCCGGACTTCC 6968
GCCGCCCCTGGGGCCCCCGTCGAATCCGCGCCGGGTCGCGCTGATGGTCGTAGGGCCAGGCGGCGCGCCGAATCCCGGACTTCC

17656 CACCATCTCCGCTGGTACCAACCAGGGCACAACCGCCGGGCCCCACCAGGGCTCAGCCGCCGCGCACCGCCGCCCCGGCTCAAC 17749
6969 CACCATCTCCGCTGGTACCAACCAGGGCACAACCGCCGGGCCCCACCAGGGCTCAGCCGCCGCGCACCGCCGCCCCGGCTCAAC 7062
CACCATCTCCGCTGGTACCAACCAGGGCACAACCGCCGGGCCCCACCAGGGCTCAGCCGCCGCGCACCGCCGCCCCGGCTCAAC

17750 GCCCCTGCCGGACGCCGGCGCCCCGGCCAAGCAGCCAGGGAAGCCGAATAAGCCGGTGCCACAACGGGGTTGGCGGCGGTGGG 17843
7063 GCCCCTGCCGGACGCCGGCGCCCCGGCCAAGCAGCCAGGGAAGCCGAATAAGCCGGTGCCACAACGGGGTTGGCGGCGGTGGG 7156
GCCCCTGCCGGACGCCGGCGCCCCGGCCAAGCAGCCAGGGAAGCCGAATAAGCCGGTGCCACAACGGGGTTGGCGGCGGTGGG

17844 ACGCGGATAAACTTCGGCCTTTCTCCCGATGAGAAGTATGAATTGGACCTGCGCACGCGGATCGGTCGAAAGCCCCGCGGCTCGT 17937
7157 ACGCGGATAAACTTCGGCCTTTCTCCCGATGAGAAGTATGAATTGGACCTGCGCACGCGGATCGGTCGAAAGCCCCGCGGCTCGT 7250
ACGCGGATAAACTTCGGCCTTTCTCCCGATGAGAAGTATGAATTGGACCTGCGCACGCGGATCGGTCGAAAGCCCCGCGGCTCGT

17938 CGATCTTGGGCCTCAAGGGCGGCGCCGGGAAGACGACCACGACGGTCACTCTTGGCACCACACTGACGCACGTGCGCGGGGATC 18031
7251 CGATCTTGGGCCTCAAGGGCGGCGCCGGGAAGACGACCACGACGGTCACTCTTGGCACCACACTGACGCACGTGCGCGGGGATC 7344
CGATCTTGGGCCTCAAGGGCGGCGCCGGGAAGACGACCACGACGGTCACTCTTGGCACCACACTGACGCACGTGCGCGGGGATC

18032 GCTCGACGCCGATCCGGGCGCCGGAAATCTCGCCGAACGTTCGGGACGTTCGTCGCCATCATCGATTGCCGATCTGCTGGCGGA 18125
7345 GCTCGACGCCGATCCGGGCGCCGGAAATCTCGCCGAACGTTCGGGACGTTCGTCGCCATCATCGATTGCCGATCTGCTGGCGGA 7438
GCTCGACGCCGATCCGGGCGCCGGAAATCTCGCCGAACGTTCGGGACGTTCGTCGCCATCATCGATTGCCGATCTGCTGGCGGA

18126 TCGCACTACAACGATGTCCGCGCACACACCAGCGTCAATGCCGCCAATCTCGAAATTCTCCCCACCGCGGAATACACCTCCGCGC 18219
7439 TCGCACTACAACGATGTCCGCGCACACACCAGCGTCAATGCCGCCAATCTCGAAATTCTCCCCACCGCGGAATACACCTCCGCGC 7532
TCGCACTACAACGATGTCCGCGCACACACCAGCGTCAATGCCGCCAATCTCGAAATTCTCCCCACCGCGGAATACACCTCCGCGC

18220 TCAGCGGCGAAGACTTGCGATCGGCCGTCGATACCGTGTCGAAGTTCTACAACCTGGTCCTGGCCGATTGCGGGGCAGGGCTATT 18313
7533 TCAGCGGCGAAGACTTGCGATCGGCCGTCGATACCGTGTCGAAGTTCTACAACCTGGTCCTGGCCGATTGCGGGGCAGGGCTATT 7626
TCAGCGGCGAAGACTTGCGATCGGCCGTCGATACCGTGTCGAAGTTCTACAACCTGGTCCTGGCCGATTGCGGGGCAGGGCTATT

18314 GACACTGGGTGTGCTCGATACCGCCTCAGCGATCGTGATCTTGACTAACGTTTCCATCGACAGTGCGCGCCAAGCCGCAATCGCA 18407
7627 GACACTGGGTGTGCTCGATACCGCCTCAGCGATCGTGATCTTGACTAACGTTTCCATCGACAGTGCGCGCCAAGCCGCAATCGCA 7720
GACACTGGGTGTGCTCGATACCGCCTCAGCGATCGTGATCTTGACTAACGTTTCCATCGACAGTGCGCGCCAAGCCGCAATCGCA

18408 TTGCGCAAACACGGTTACCAGGATTTGGCGAGCCGCGCATGCGTAGCGATAAACCATGTCGCCGTTGGCGAAACCAACGTGTCGC 18501
7721 TTGCGCAAACACGGTTACCAGGATTTGGCGAGCCGCGCATGCGTAGCGATAAACCATGTCGCCGTTGGCGAAACCAACGTGTCGC 7814
TTGCGCAAACACGGTTACCAGGATTTGGCGAGCCGCGCATGCGTAGCGATAAACCATGTCGCCGTTGGCGAAACCAACGTGTCGC

18502 TGGTCCGGGACTTTGAACAGCAGCTCCAACCCGGGCGCGTAGTGGTCTTGCCATGGGACCGGCACATCGCGGCCGGCACCGAAA 18595
7815 TGGTCCGGGACTTTGAACAGCAGCTCCAACCCGGGCGCGTAGTGGTCTTGCCATGGGACCGGCACATCGCGGCCGGCACCGAAA 7908

TGGTCCGGGACTTTGAACAGCAGCTCCAACCCGGGCGCGTAGTGGTCTTGCCATGGGACCGGCACATCGCGGCCGGCACCGAAA

18596 CCAGCTTGGCCCCGTCTACCGACGACGGGTTCTCGAGCTGGCCGCGGCTCTGTCCGACGATTTTGAAAGGGCTGGACGTCGTTG 18689
7909 CCAGCTTGGCCCCGTCTACCGACGACGGGTTCTCGAGCTGGCCGCGGCTCTGTCCGACGATTTTGAAAGGGCTGGACGTCGTTG 8002

CCAGCTTGGCCCCGTCTACCGACGACGGGTTCTCGAGCTGGCCGCGGCTCTGTCCGACGATTTTGAAAGGGCTGGACGTCGTTG

18690 CAGTTGCCGCCCCCTCCGCCGCCACGGGAACCACCCCACCCAAGCCGGCAACCACCCGGGTCACCGTCCTTACCGGCAGACGGA 18783
8003 CAGTTGCCGCCCCCTCCGCCGCCACGGGAACCACCCCACCCAAGCCGGCAACCACCCGGGTCACCGTCCTTACCGGCAGACGGA 8096

CAGTTGCCGCCCCCTCCGCCGCCACGGGAACCACCCCACCCAAGCCGGCAACCACCCGGGTCACCGTCCTTACCGGCAGACGGA

18784 GGTGCTGCCCGCGGCGGCACCCATGGAGAGCTATGTCGACGAAACCGTCGCGATCTTGGCCGATCTGCTCGAAGACACTCCCGC 18877
8097 GGTGCTGCCCGCGGCGGCACCCATGGAGAGCTATGTCGACGAAACCGTCGCGATCTTGGCCGATCTGCTCGAAGACACTCCCGC 8190

GGTGCTGCCCGCGGCGGCACCCATGGAGAGCTATGTCGACGAAACCGTCGCGATCTTGGCCGATCTGCTCGAAGACACTCCCGC

18878 GCCGGCTTCGACTTCGAAGCTCAGGGCGTTTGGACGTTCGCTCGGCCCGGATTCCCGCCGCTGAAGCTCGACCAGTCCCTCGATC 18971
8191 GCCGGCTTCGACTTCGAAGCTCAGGGCGTTTGGACGTTCGCTCGGCCCGGATTCCCGCCGCTGAAGCTCGACCAGTCCCTCGATC 8284

GCCGGCTTCGACTTCGAAGCTCAGGGCGTTTGGACGTTCGCTCGGCCCGGATTCCCGCCGCTGAAGCTCGACCAGTCCCTCGATC

18972 TTGTCGACGGATCGCTACTGACCTTGGTCTTGGCCAGTCGCACGGAGCGGTATCGGCCCCTCGTCGAGGACGTTATCGACGCGAT 19065
8285 TTGTCGACGGATCGCTACTGACCTTGGTCTTGGCCAGTCGCACGGAGCGGTATCGGCCCCTCGTCGAGGACGTTATCGACGCGAT 8378

TTGTCGACGGATCGCTACTGACCTTGGTCTTGGCCAGTCGCACGGAGCGGTATCGGCCCCTCGTCGAGGACGTTATCGACGCGAT

19066 CGACGAGTCGCCCGAGTTCAACCGCACCGCTCTAGAACGCTTCATCGCCGTGGCGATCCCCGTCTTCGCCCTGCCCATCACGGCG 19159
8379 CGACGAGTCGCCCGAGTTCAACCGCACCGCTCTAGAACGCTTCATCGCCGTGGCGATCCCCGTCTTCGCCCTGCCCATCACGGCG 8472

CGACGAGTCGCCCGAGTTCAACCGCACCGCTCTAGAACGCTTCATCGCCGTGGCGATCCCCGTCTTCGCCCTGCCCATCACGGCG

19160 CGGGCCTGGTGGCAAACCGGGCGCAGCGTGTTCTGGCCGCTGGTGATCGGCCTGATTGGGCTCGCCGCCTTGGCGAGTTCCTTC 19253
8473 CGGGCCTGGTGGCAAACCGGGCGCAGCGTGTTCTGGCCGCTGGTGATCGGCCTGATTGGGCTCGCCGCCTTGGCGAGTTCCTTC 8566

CGGGCCTGGTGGCAAACCGGGCGCAGCGTGTTCTGGCCGCTGGTGATCGGCCTGATTGGGCTCGCCGCCTTGGCGAGTTCCTTC

19254 GGTTTTACCAAAACTCGCGGCTCGCCGAGAGCTTGCTGGTCACGTCGTACGGGGTCATCGCCGCGGCGGCAGCGATTGCCGTTC 19347
8567 GGTTTTACCAAAACTCGCGGCTCGCCGAGAGCTTGCTGGTCACGTCGTACGGGGTCATCGCCGCGGCGGCAGCGATTGCCGTTC 8660

GGTTTTACCAAAACTCGCGGCTCGCCGAGAGCTTGCTGGTCACGTCGTACGGGGTCATCGCCGCGGCGGCAGCGATTGCCGTTC

19348 CGGGTTCCATTCGCTGGGGGCGCCCCAGCTCGCCGCCGCCGCCACGGCGGTGTTGTTCGTCACCTTGATGACGCGCGGCGGGCC 19441
8661 CGGGTTCCATTCGCTGGGGGCGCCCCAGCTCGCCGCCGCCGCCACGGCGGTGTTGTTCGTCACCTTGATGACGCGCGGCGGGCC 8754
CGGGTTCCATTCGCTGGGGGCGCCCCAGCTCGCCGCCGCCGCCACGGCGGTGTTGTTCGTCACCTTGATGACGCGCGGCGGGCC

19442 CACGACATCGCGGCGTTTGTCGTGATTACATCGATCGCGGTCATTTCGGCGGCGGTCGCCTTCGGATACGGGTATCAGGAATGGC 19535
8755 CACGACATCGCGGCGTTTGTCGTGATTACATCGATCGCGGTCATTTCGGCGGCGGTCGCCTTCGGATACGGGTATCAGGAATGGC 8848
CACGACATCGCGGCGTTTGTCGTGATTACATCGATCGCGGTCATTTCGGCGGCGGTCGCCTTCGGATACGGGTATCAGGAATGGC

19536 GGGCGATTGCGTTCGGGTTGTTCGTCGTGACGAACGCGGCCAAGCTCACTGTCGCCGTCGCGCGGATCGCGCTACCGCCCATCC 19629
8849 GGGCGATTGCGTTCGGGTTGTTCGTCGTGACGAACGCGGCCAAGCTCACTGTCGCCGTCGCGCGGATCGCGCTACCGCCCATCC 8942
GGGCGATTGCGTTCGGGTTGTTCGTCGTGACGAACGCGGCCAAGCTCACTGTCGCCGTCGCGCGGATCGCGCTACCGCCCATCC

19630 AGAGACCGTGGACAACGAGGAATTGCTCGATCCCATCACGGCCCAAGACGCGACCAACGAAGAGACACCAACCTGGCAAGCCAT 19723
8943 AGAGACCGTGGACAACGAGGAATTGCTCGATCCCATCACGGCCCAAGACGCGACCAACGAAGAGACACCAACCTGGCAAGCCAT 9036
AGAGACCGTGGACAACGAGGAATTGCTCGATCCCATCACGGCCCAAGACGCGACCAACGAAGAGACACCAACCTGGCAAGCCAT

19724 GCGCCGGCGTCGGCAGCCCGGCTCACTGAGCGCAGCAAATTGGCTAAGCAGCTGCTCGTCGGCTACGTCACCGCGGGAACGCTG 19817
9037 GCGCCGGCGTCGGCAGCCCGGCTCACTGAGCGCAGCAAATTGGCTAAGCAGCTGCTCGTCGGCTACGTCACCGCGGGAACGCTG 9130
GCGCCGGCGTCGGCAGCCCGGCTCACTGAGCGCAGCAAATTGGCTAAGCAGCTGCTCGTCGGCTACGTCACCGCGGGAACGCTG

19818 TGGGTTCGATAGCCGTGGTGGTACACGGGCACTTCTTCATACACAGCCTGATTGTGGCGGGTCTGATCACGGTGATCTGTTCGTT 19911
9131 TGGGTTCGATAGCCGTGGTGGTACACGGGCACTTCTTCATACACAGCCTGATTGTGGCGGGTCTGATCACGGTGATCTGTTCGTT 9224
TGGGTTCGATAGCCGTGGTGGTACACGGGCACTTCTTCATACACAGCCTGATTGTGGCGGGTCTGATCACGGTGATCTGTTCGTT

19912 GCTGTACGCGGATCCGTGGTGCGCATGGGCGCTGCTGGCGGCGACCGTCGCCATACCGACCGGGCTTGCCGTGAAGCTGAGCCT 20005
9225 GCTGTACGCGGATCCGTGGTGCGCATGGGCGCTGCTGGCGGCGACCGTCGCCATACCGACCGGGCTTGCCGTGAAGCTGAGCCT 9318
GCTGTACGCGGATCCGTGGTGCGCATGGGCGCTGCTGGCGGCGACCGTCGCCATACCGACCGGGCTTGCCGTGAAGCTGAGCCT

20006 CACTATGCCTGGCTGTTGTTGACCATCTACCTCGCCGCGGCCGTCATTACGCTCATCTCGGTCGGGGCGATGAACCAGGTACGTC 20099
9319 CACTATGCCTGGCTGTTGTTGACCATCTACCTCGCCGCGGCCGTCATTACGCTCATCTCGGTCGGGGCGATGAACCAGGTACGTC 9412
CACTATGCCTGGCTGTTGTTGACCATCTACCTCGCCGCGGCCGTCATTACGCTCATCTCGGTCGGGGCGATGAACCAGGTACGTC

20100 CGGTCATGAAACGGGCGCTGGAATTGTTCGATGGCGCGATGATCGCATCAATCGCCCCGCTGCTGCTCTGGATTACCGGTGTCTA 20193
9413 CGGTCATGAAACGGGCGCTGGAATTGTTCGATGGCGCGATGATCGCATCAATCGCCCCGCTGCTGCTCTGGATTACCGGTGTCTA 9506
CGGTCATGAAACGGGCGCTGGAATTGTTCGATGGCGCGATGATCGCATCAATCGCCCCGCTGCTGCTCTGGATTACCGGTGTCTA

20194 CCGCAATATCCGATTCTGAGTCACCGGCGGGCTACGTCCGGGCGGTCGTGGTAGCGGATCGGCCCGCACCGAAATTGGCGCAAT 20287
9507 CCGCAATATCCGATTCTGAGTCACCGGCGGGCTACGTCCGGGCGGTCGTGGTAGCGGATCGGCCCGCACCGAAATTGGCGCAAT 9600
CCGCAATATCCGATTCTGAGTCACCGGCGGGCTACGTCCGGGCGGTCGTGGTAGCGGATCGGCCCGCACCGAAATTGGCGCAAT

20288 CCAGGTCGGGTAAAATTTGCTGAATCCACTACGTACAGGAGGGATTCTGCGATGGCTGAGCCTCTGGCCGTCGATCCCGCCCGTC 20381
9601 CCAGGTCGGGTAAAATTTGCTGAATCCACTACGTACAGGAGGGATTCTGCGATGGCTGAGCCTCTGGCCGTCGATCCCGCCCGTC 9694
CCAGGTCGGGTAAAATTTGCTGAATCCACTACGTACAGGAGGGATTCTGCGATGGCTGAGCCTCTGGCCGTCGATCCCGCCCGTC

20382 CGGGAAGCAAGCTCGCCGAGCTGGTTTTTCCGGCGCCACCAGCGCCGATAGCAGCAACTGGAGGGGATCCGGTTTCGGCTGCAA 20475
9695 CGGGAAGCAAGCTCGCCGAGCTGGTTTTTCCGGCGCCACCAGCGCCGATAGCAGCAACTGGAGGGGATCCGGTTTCGGCTGCAA 9788
CGGGAAGCAAGCTCGCCGAGCTGGTTTTTCCGGCGCCACCAGCGCCGATAGCAGCAACTGGAGGGGATCCGGTTTCGGCTGCAA

20476 AATGCCTGGCATCGAGTCCTTGGTGTCCGATGGGATGCCCGGTGTAACCGCCGCCTTGAAACGAACCGCTTCCAGCATGTCGACT 20569
9789 AATGCCTGGCATCGAGTCCTTGGTGTCCGATGGGATGCCCGGTGTAACCGCCGCCTTGAAACGAACCGCTTCCAGCATGTCGACT 9882
AATGCCTGGCATCGAGTCCTTGGTGTCCGATGGGATGCCCGGTGTAACCGCCGCCTTGAAACGAACCGCTTCCAGCATGTCGACT

20570 ATCTACGCCAAAGCCGACCAAGCCCTTGGCGATGCACTGACGCAGTACCAATTCGGCGGCGACGGCCAAGCGCTGGGCGCAAGC 20663
9883 ATCTACGCCAAAGCCGACCAAGCCCTTGGCGATGCACTGACGCAGTACCAATTCGGCGGCGACGGCCAAGCGCTGGGCGCAAGC 9976
ATCTACGCCAAAGCCGACCAAGCCCTTGGCGATGCACTGACGCAGTACCAATTCGGCGGCGACGGCCAAGCGCTGGGCGCAAGC

20664 CTGTGGCACAGAGCCAGACCGTGCAGTCGTTGGCCGCGCCCGCCGCCGGGCTATTGGGCGCGCCCGTGGCGCAAGCATTGGCCC 20757
9977 CTGTGGCACAGAGCCAGACCGTGCAGTCGTTGGCCGCGCCCGCCGCCGGGCTATTGGGCGCGCCCGTGGCGCAAGCATTGGCCC 10070
CTGTGGCACAGAGCCAGACCGTGCAGTCGTTGGCCGCGCCCGCCGCCGGGCTATTGGGCGCGCCCGTGGCGCAAGCATTGGCCC

20758 CGGGCTGCTGGGTGCACCCGCGGCCGCGGCGACACAGATCGGCGAGGCGGTCAGCGCTCAGGCGGAAGCCCTCTCGCCCCGTG 20851
10071 CGGGCTGCTGGGTGCACCCGCGGCCGCGGCGACACAGATCGGCGAGGCGGTCAGCGCTCAGGCGGAAGCCCTCTCGCCCCGTG 10164
CGGGCTGCTGGGTGCACCCGCGGCCGCGGCGACACAGATCGGCGAGGCGGTCAGCGCTCAGGCGGAAGCCCTCTCGCCCCGTG

20852 ATTCCCCAGCTGGTGCAACTGGCCCCGCAGGCCGGTCAGATGGCGCAGCAGGCCTCACCGATCGCGCAGACCATCAGTCAGTCG 20945
10165 ATTCCCCAGCTGGTGCAACTGGCCCCGCAGGCCGGTCAGATGGCGCAGCAGGCCTCACCGATCGCGCAGACCATCAGTCAGTCG 10258
ATTCCCCAGCTGGTGCAACTGGCCCCGCAGGCCGGTCAGATGGCGCAGCAGGCCTCACCGATCGCGCAGACCATCAGTCAGTCG

20946 GGTCCTCGCAGGGCGGCGCAGCGCCGGCACAACTCGTCTCGGACACCAAACCCGACGAAGACGCGGAGCTGGCTGACGAGACCA 21039
10259 GGTCCTCGCAGGGCGGCGCAGCGCCGGCACAACTCGTCTCGGACACCAAACCCGACGAAGACGCGGAGCTGGCTGACGAGACCA 10352
GGTCCTCGCAGGGCGGCGCAGCGCCGGCACAACTCGTCTCGGACACCAAACCCGACGAAGACGCGGAGCTGGCTGACGAGACCA

21040 AGAAGACGCCGCGGCAGCGGCCGGTGCAGAAGGCGCCGCGGCCGGCCACGCCACACTGGTGAGCGCTCCCGTCGAAAGCACCG 21133
10353 AGAAGACGCCGCGGCAGCGGCCGGTGCAGAAGGCGCCGCGGCCGGCCACGCCACACTGGTGAGCGCTCCCGTCGAAAGCACCG 10446

AGAAGACGCCGCGGCAGCGGCCGGTGCAGAAGGCGCCGCGGCCGGCCACGCCACACTGGTGAGCGCTCCCGTCGAAAGCACCG

21134 ACGTCGACGGGTTCGGTCTCAGCTCCGATCTGATAATTCGGCGTAAGCCGCGCCCCGATCGGGTGTACGGCGGATACGCGCGGT 21227
10447 ACGTCGACGGGTTCGGTCTCAGCTCCGATCTGATAATTCGGCGTAAGCCGCGCCCCGATCGGGTGTACGGCGGATACGCGCGGT 10540

ACGTCGACGGGTTCGGTCTCAGCTCCGATCTGATAATTCGGCGTAAGCCGCGCCCCGATCGGGTGTACGGCGGATACGCGCGGT

21228 ACATCCAACCTCGGCCGGGCGCCAAATGGGAGCGTGCCCACATCGGGCGCCGTCATACCGGCCAACCCGGGGATCGCGTCCGAC 21321
10541 ACATCCAACCTCGGCCGGGCGCCAAATGGGAGCGTGCCCACATCGGGCGCCGTCATACCGGCCAACCCGGGGATCGCGTCCGAC 10634

ACATCCAACCTCGGCCGGGCGCCAAATGGGAGCGTGCCCACATCGGGCGCCGTCATACCGGCCAACCCGGGGATCGCGTCCGAC

21322 GTGCCGACCGTGCCGCCAACATTTCGGTCGCTCGCTGTGTCGCCGCCCGCAGGCTGCTGGCGTTCGAGGAGGTCTTGTTCGCCA 21415
10635 GTGCCGACCGTGCCGCCAACATTTCGGTCGCTCGCTGTGTCGCCGCCCGCAGGCTGCTGGCGTTCGAGGAGGTCTTGTTCGCCA 10728

GTGCCGACCGTGCCGCCAACATTTCGGTCGCTCGCTGTGTCGCCGCCCGCAGGCTGCTGGCGTTCGAGGAGGTCTTGTTCGCCA

21416 GGTGGCGGCGGCGGCAACCTGCACAACACCGCATGCGGCGACCGCAATGTGGAAGGACGGGAGCTGGCCCTCGGGCCCCCCTA 21509
10729 GGTGGCGGCGGCGGCAACCTGCACAACACCGCATGCGGCGACCGCAATGTGGAAGGACGGGAGCTGGCCCTCGGGCCCCCCTA 10822

GGTGGCGGCGGCGGCAACCTGCACAACACCGCATGCGGCGACCGCAATGTGGAAGGACGGGAGCTGGCCCTCGGGCCCCCCTA

21510 GCACAAACCACCAGCACACCCAGGACTACCGCGATCAGTACCGACAGCCGCTCGCGGGCTTGCTTGACGGCATCGGCTTGGGCG 21603
10823 GCACAAACCACCAGCACACCCAGGACTACCGCGATCAGTACCGACAGCCGCTCGCGGGCTTGCTTGACGGCATCGGCTTGGGCG 10916

GCACAAACCACCAGCACACCCAGGACTACCGCGATCAGTACCGACAGCCGCTCGCGGGCTTGCTTGACGGCATCGGCTTGGGCG

21604 CGGCGGTCAGCCGGTCCAGGTCGGCCATCAGCGTGGCGTGCCGCGACTGTGCGAGGTTCTGAACCCAATAGGCCTGCGCCGCAT 21697
10917 CGGCGGTCAGCCGGTCCAGGTCGGCCATCAGCGTGGCGTGCCGCGACTGTGCGAGGTTCTGAACCCAATAGGCCTGCGCCGCAT 11010

CGGCGGTCAGCCGGTCCAGGTCGGCCATCAGCGTGGCGTGCCGCGACTGTGCGAGGTTCTGAACCCAATAGGCCTGCGCCGCAT

21698 ACCGCCGTCCGGATCAAGGGCGGCGACCTGGGCGCCGACGTCGTCGAACAACGACCCACTGCTCGCCAAGCGATCACCCTGCTC 21791
11011 ACCGCCGTCCGGATCAAGGGCGGCGACCTGGGCGCCGACGTCGTCGAACAACGACCCACTGCTCGCCAAGCGATCACCCTGCTC 11104

ACCGCCGTCCGGATCAAGGGCGGCGACCTGGGCGCCGACGTCGTCGAACAACGACCCACTGCTCGCCAAGCGATCACCCTGCTC

21792 TCTCCAAGCCATCCCCACATGACACCAAGGATGGTCAGGGTCGACGCGCAAACAACGGTTCCTCGGCATAGCTCGTGGGGATTAT 21885
11105 TCTCCAAGCCATCCCCACATGACACCAAGGATGGTCAGGGTCGACGCGCAAACAACGGTTCCTCGGCATAGCTCGTGGGGATTAT 11198

TCTCCAAGCCATCCCCACATGACACCAAGGATGGTCAGGGTCGACGCGCAAACAACGGTTCCTCGGCATAGCTCGTGGGGATTAT

21886 CCGCTTCCGCTTCTCGCTCAATCTCCCCTACGTCCGATTCGAATCCGTCAGGTTCAAACGGTCCTGCGTCCGGCGCGAAGCGGTC 21979
11199 CCGCTTCCGCTTCTCGCTCAATCTCCCCTACGTCCGATTCGAATCCGTCAGGTTCAAACGGTCCTGCGTCCGGCGCGAAGCGGTC 11292

CCGCTTCCGCTTCTCGCTCAATCTCCCCTACGTCCGATTCGAATCCGTCAGGTTCAAACGGTCCTGCGTCCGGCGCGAAGCGGTC

21980 TTCAAGCATTCGGTCATATGTGGCCGCTTCATCCGGGTGCATGAGGCGAAGATTCCTTTCGAGCTATCGTTGTGCCGACCCAGCA[illegible] 22073
11293 TTCAAGCATTCGGTCATATGTGGCCGCTTCATCCGGGTGCATGAGGCGAAGATTCCTTTCGAGCTATCGTTGTGCCGACCCAGCA[illegible] 11386

TTCAAGCATTCGGTCATATGTGGCCGCTTCATCCGGGTGCATGAGGCGAAGATTCCTTTCGAGCTATCGTTGTGCCGACCCAGCA[illegible]

22074 TGCATCGTCATGTCAAAACCGGGTGGTGGGCTGCTGCGGACCCACGTACCGCCCGAAACCCGTTCAACACGTTGCTAGGCCGAG[illegible] 22167
11387 TGCATCGTCATGTCAAAACCGGGTGGTGGGCTGCTGCGGACCCACGTACCGCCCGAAACCCGTTCAACACGTTGCTAGGCCGAG[illegible] 11480

TGCATCGTCATGTCAAAACCGGGTGGTGGGCTGCTGCGGACCCACGTACCGCCCGAAACCCGTTCAACACGTTGCTAGGCCGAG[illegible]

22168 GTCCGCACGCCGCCGCACTCGAGAGCGAACATCGGGTGTTCGCATCTGGTCTCCCGCGCCGAACCGCCCCGTAAGGCACCAGCA 22261
11481 GTCCGCACGCCGCCGCACTCGAGAGCGAACATCGGGTGTTCGCATCTGGTCTCCCGCGCCGAACCGCCCCGTAAGGCACCAGCA 11574

GTCCGCACGCCGCCGCACTCGAGAGCGAACATCGGGTGTTCGCATCTGGTCTCCCGCGCCGAACCGCCCCGTAAGGCACCAGCA

22262 ACGAATTCGCCGGATCGCGCACTAAATGCGGGCCTGCAAATTTCAGAATCAACTTCTGAAATACGTCACAGAAGAATCGTGCATG[illegible] 22355
11575 ACGAATTCGCCGGATCGCGCACTAAATGCGGGCCTGCAAATTTCAGAATCAACTTCTGAAATACGTCACAGAAGAATCGTGCATG[illegible] 11668

ACGAATTCGCCGGATCGCGCACTAAATGCGGGCCTGCAAATTTCAGAATCAACTTCTGAAATACGTCACAGAAGAATCGTGCATG[illegible]

22356 ATCGACTATAACTTACATTCGCAAAATGCACTGGCAGCGGCCTTCTTCAGGCACGCGCGCAGCGGTGTCGTCTCCACTCGGTCCT[illegible] 22449
11669 ATCGACTATAACTTACATTCGCAAAATGCACTGGCAGCGGCCTTCTTCAGGCACGCGCGCAGCGGTGTCGTCTCCACTCGGTCCT[illegible] 11762

ATCGACTATAACTTACATTCGCAAAATGCACTGGCAGCGGCCTTCTTCAGGCACGCGCGCAGCGGTGTCGTCTCCACTCGGTCCT[illegible]

22450 TCGGATCACTCCGAATTCAGGACGCGTCCGCCAAAGCGTTGGCAAGCAGCCCGGCGACATAACGCCAGTACAGCCAGTCGGCGA[illegible] 22543
11763 TCGGATCACTCCGAATTCAGGACGCGTCCGCCAAAGCGTTGGCAAGCAGCCCGGCGACATAACGCCAGTACAGCCAGTCGGCGA[illegible] 11856

TCGGATCACTCCGAATTCAGGACGCGTCCGCCAAAGCGTTGGCAAGCAGCCCGGCGACATAACGCCAGTACAGCCAGTCGGCGA[illegible]

22544 CTGGGCTTCAGCGTCTGCCGCGCTGTATGCCTGGTGCAGCGCGATCTCCTGGCAATGTTCGGCATAGGCGCGGAATGCACGCAG[illegible] 22637
11857 CTGGGCTTCAGCGTCTGCCGCGCTGTATGCCTGGTGCAGCGCGATCTCCTGGCAATGTTCGGCATAGGCGCGGAATGCACGCAG[illegible] 11950

CTGGGCTTCAGCGTCTGCCGCGCTGTATGCCTGGTGCAGCGCGATCTCCTGGCAATGTTCGGCATAGGCGCGGAATGCACGCAG[illegible]

22638 TCGCGTCCGGTGGCGTTACTCGTCATCGGCTTCATCAGGTCGAACCAGAACATGTGCCGCTCGTCGTCTGGCGGATTGGCGTCG[illegible] 22731
11951 TCGCGTCCGGTGGCGTTACTCGTCATCGGCTTCATCAGGTCGAACCAGAACATGTGCCGCTCGTCGTCTGGCGGATTGGCGTCG[illegible] 12044

TCGCGTCCGGTGGCGTTACTCGTCATCGGCTTCATCAGGTCGAACCAGAACATGTGCCGCTCGTCGTCTGGCGGATTGGCGTCG[illegible]

22732 GCGGCAGCAACTCCAGCAGCCGCAGATCTTCGGTCTCTGCCAGCTGGGCCGCCGCCGAGGGGTCTACCACCTCGAGCCGCGGAC 22825
12045 GCGGCAGCAACTCCAGCAGCCGCAGATCTTCGGTCTCTGCCAGCTGGGCCGCCGCCGAGGGGTCTACCACCTCGAGCCGCGGAC 12138
GCGGCAGCAACTCCAGCAGCCGCAGATCTTCGGTCTCTGCCAGCTGGGCCGCCGCCGAGGGGTCTACCACCTCGAGCCGCGGAC

22826 CTTGCCGCTCTCCGGAATGTCGTCGGCTTCCAGGATGATCTTGGCCGCGCCCGCGTCCGAGTTGGCCAATTGTTCGGCCGTCCCG 22919
12139 CTTGCCGCTCTCCGGAATGTCGTCGGCTTCCAGGATGATCTTGGCCGCGCCCGCGTCCGAGTTGGCCAATTGTTCGGCCGTCCCG 12232
CTTGCCGCTCTCCGGAATGTCGTCGGCTTCCAGGATGATCTTGGCCGCGCCCGCGTCCGAGTTGGCCAATTGTTCGGCCGTCCCG

22920 CGCAGCTTCAAGTCGTGAAACGCCGCCCAACCCTGCACGGCCAACACCGGGTAGGTGGCGAACCGGGCCTGCTCATCGGCCGGG 23013
12233 CGCAGCTTCAAGTCGTGAAACGCCGCCCAACCCTGCACGGCCAACACCGGGTAGGTGGCGAACCGGGCCTGCTCATCGGCCGGG 12326
CGCAGCTTCAAGTCGTGAAACGCCGCCCAACCCTGCACGGCCAACACCGGGTAGGTGGCGAACCGGGCCTGCTCATCGGCCGGG

23014 CAGCGCTGGCCATGTAGACCTTGTGCGGTAGCTCGACATTCTCGGGTATGTAGGCCAGCCCATAACTGTTGGCCACCACGATGTC 23107
12327 CAGCGCTGGCCATGTAGACCTTGTGCGGTAGCTCGACATTCTCGGGTATGTAGGCCAGCCCATAACTGTTGGCCACCACGATGTC 12420
CAGCGCTGGCCATGTAGACCTTGTGCGGTAGCTCGACATTCTCGGGTATGTAGGCCAGCCCATAACTGTTGGCCACCACGATGTC

23108 GGTCACCGCGGTGATCCAGAAGAACCCGTAGTCGCCCTTGTTGTAGGTGTCGGGCGCGTTCAACGCGGCCGCGATGCGTCGCGC 23201
12421 GGTCACCGCGGTGATCCAGAAGAACCCGTAGTCGCCCTTGTTGTAGGTGTCGGGCGCGTTCAACGCGGCCGCGATGCGTCGCGC 12514
GGTCACCGCGGTGATCCAGAAGAACCCGTAGTCGCCCTTGTTGTAGGTGTCGGGCGCGTTCAACGCGGCCGCGATGCGTCGCGC

23202 GGGTCCTTCTTTTGACTGCGACGAGACGCCGAAGCGATGGCATCGCGCGCGGCCCGCGCCGCCGAGACCGGAACCATCGGCGAT 23295
12515 GGGTCCTTCTTTTGACTGCGACGAGACGCCGAAGCGATGGCATCGCGCGCGGCCCGCGCCGCCGAGACCGGAACCATCGGCGAT 12608
GGGTCCTTCTTTTGACTGCGACGAGACGCCGAAGCGATGGCATCGCGCGCGGCCCGCGCCGCCGAGACCGGAACCATCGGCGAT

23296 CATCGGGCGCTTCGGACTTTTCTTTTCCGTCCGGCTTGGGTCGCTCCGGAGCCGGACGGGTCGGCGATGCGGTGCGAGCCGCCG 23389
12609 CATCGGGCGCTTCGGACTTTTCTTTTCCGTCCGGCTTGGGTCGCTCCGGAGCCGGACGGGTCGGCGATGCGGTGCGAGCCGCCG 12702
CATCGGGCGCTTCGGACTTTTCTTTTCCGTCCGGCTTGGGTCGCTCCGGAGCCGGACGGGTCGGCGATGCGGTGCGAGCCGCCG

23390 CGCGGCCGTATTGGGGCCCCGGCCGCCCGACTCCACCGGTGCGCGCCCCGAGGCGGCACGCGAGCCGGCACCCGAGGCCGCTG 23483
12703 CGCGGCCGTATTGGGGCCCCGGCCGCCCGACTCCACCGGTGCGCGCCCCGAGGCGGCACGCGAGCCGGCACCCGAGGCCGCTG 12796
CGCGGCCGTATTGGGGCCCCGGCCGCCCGACTCCACCGGTGCGCGCCCCGAGGCGGCACGCGAGCCGGCACCCGAGGCCGCTG

23484 CTCGAACTCGCTCCGGCCGAGGACAGGCCCGAGCTTGATCCTGCGGACACTCCTCGACCGCCGCCCGGCATCGCGCTGGCGGCA 23577
12797 CTCGAACTCGCTCCGGCCGAGGACAGGCCCGAGCTTGATCCTGCGGACACTCCTCGACCGCCGCCCGGCATCGCGCTGGCGGCA 12890
CTCGAACTCGCTCCGGCCGAGGACAGGCCCGAGCTTGATCCTGCGGACACTCCTCGACCGCCGCCCGGCATCGCGCTGGCGGCA

23578 CGGCCCCTCCCGACGAGTCGTCGCCGTGCGCAGGGCCCGAAGGTCCCGACGGCTGGGTTGACGGTGCCGTGGACGTCTCCGTTG 23671
12891 CGGCCCCTCCCGACGAGTCGTCGCCGTGCGCAGGGCCCGAAGGTCCCGACGGCTGGGTTGACGGTGCCGTGGACGTCTCCGTTG 12984
CGGCCCCTCCCGACGAGTCGTCGCCGTGCGCAGGGCCCGAAGGTCCCGACGGCTGGGTTGACGGTGCCGTGGACGTCTCCGTTG

23672 CTTCACGTGAGCCGGGTCGGTTGACGGCTGCCCCGGCGTGGACGGCGACCCCGGCTGCGATGGCGCGGGCTGCGGACTCGCGC 23765
12985 CTTCACGTGAGCCGGGTCGGTTGACGGCTGCCCCGGCGTGGACGGCGACCCCGGCTGCGATGGCGCGGGCTGCGGACTCGCGC 13078
CTTCACGTGAGCCGGGTCGGTTGACGGCTGCCCCGGCGTGGACGGCGACCCCGGCTGCGATGGCGCGGGCTGCGGACTCGCGC

23766 GGCCCCGGCTGCGGACCGGGCCCCGGAGCTGGCGCGGGCTGCGGTGCCGGCGCTGGGGCAGGTGCCGGGGCGGGCGCAGGGG 23859
13079 GGCCCCGGCTGCGGACCGGGCCCCGGAGCTGGCGCGGGCTGCGGTGCCGGCGCTGGGGCAGGTGCCGGGGCGGGCGCAGGGG 13172
GGCCCCGGCTGCGGACCGGGCCCCGGAGCTGGCGCGGGCTGCGGTGCCGGCGCTGGGGCAGGTGCCGGGGCGGGCGCAGGGG

23860 CCGGCGCTGGGGCCGGCGCTGGGGCAGGGGTTGGCTCGACCGGCAAGACCGGGGTGTCGGGCTTCCCTGGCTTCGGCTTGCCC 23953
13173 CCGGCGCTGGGGCCGGCGCTGGGGCAGGGGTTGGCTCGACCGGCAAGACCGGGGTGTCGGGCTTCCCTGGCTTCGGCTTGCCC 13266
CCGGCGCTGGGGCCGGCGCTGGGGCAGGGGTTGGCTCGACCGGCAAGACCGGGGTGTCGGGCTTCCCTGGCTTCGGCTTGCCC

23954 CGTCACCGGCTTGCCCGGAGTACCTGGGGTGATCGGAGTACCCGGGATACCCGGGATACCCGGGGTCCCCGGGATACCCGGGAT 24047
13267 CGTCACCGGCTTGCCCGGAGTACCTGGGGTGATCGGAGTACCCGGGATACCCGGGATACCCGGGGTCCCCGGGATACCCGGGAT 13360
CGTCACCGGCTTGCCCGGAGTACCTGGGGTGATCGGAGTACCCGGGATACCCGGGATACCCGGGGTCCCCGGGATACCCGGGAT

24048 CCCGGGGTAACCGGGTGCGTTGGCCCGCCGGGTGTCGGAACCACCGGAGCACCCGGCTTCGGGTTCACCGGCGTGCCCGGTCC 24141
13361 CCCGGGGTAACCGGGTGCGTTGGCCCGCCGGGTGTCGGAACCACCGGAGCACCCGGCTTCGGGTTCACCGGCGTGCCCGGTCC 13454
CCCGGGGTAACCGGGTGCGTTGGCCCGCCGGGTGTCGGAACCACCGGAGCACCCGGCTTCGGGTTCACCGGCGTGCCCGGTCC

24142 CCGGCTTGTGCGGGTTGACCGGCGTGGGTTCGAACGGCTTCGGTCCCGGTGGCCTCGGGGCTGGCTGACCTGGCGTCGGCACGC 24235
13455 CCGGCTTGTGCGGGTTGACCGGCGTGGGTTCGAACGGCTTCGGTCCCGGTGGCCTCGGGGCTGGCTGACCTGGCGTCGGCACGC 13548
CCGGCTTGTGCGGGTTGACCGGCGTGGGTTCGAACGGCTTCGGTCCCGGTGGCCTCGGGGCTGGCTGACCTGGCGTCGGCACGC

24236 AATGCCCGGGGCCGGAGGCGTCACCTGGTGCAGCAGATCCTTGAGCGCATTGTGCGGCGGCTTCCAGTTTCTGGATGCCAGAAC 24329
13549 AATGCCCGGGGCCGGAGGCGTCACCTGGTGCAGCAGATCCTTGAGCGCATTGTGCGGCGGCTTCCAGTTTCTGGATGCCAGAAC 13642
AATGCCCGGGGCCGGAGGCGTCACCTGGTGCAGCAGATCCTTGAGCGCATTGTGCGGCGGCTTCCAGTTTCTGGATGCCAGAAC

24330 GCTTCGGCCACCAGACCCGAGTTGGCCTCGTGAGCTGAGCGGATCAGGGATGCGATCGCCGCCTTGCGCTCCTCGGGGTCAAGG 24423
13643 GCTTCGGCCACCAGACCCGAGTTGGCCTCGTGAGCTGAGCGGATCAGGGATGCGATCGCCGCCTTGCGCTCCTCGGGGTCAAGG 13736
GCTTCGGCCACCAGACCCGAGTTGGCCTCGTGAGCTGAGCGGATCAGGGATGCGATCGCCGCCTTGCGCTCCTCGGGGTCAAGG

24424 CCTCCAGAACCCGTATTTCGCGATGAGCACCGTCCACGTTGTTGTCAATGTTTGCCTTCGCCTCGGCGATCAACCCAGCCACATG 24517
13737 CCTCCAGAACCCGTATTTCGCGATGAGCACCGTCCACGTTGTTGTCAATGTTTGCCTTCGCCTCGGCGATCAACCCAGCCACATG 13830
CCTCCAGAACCCGTATTTCGCGATGAGCACCGTCCACGTTGTTGTCAATGTTTGCCTTCGCCTCGGCGATCAACCCAGCCACATG

24518 GGTAATAACCGTGGCGAGATAGTCCTGCAGCGTGCTCATTTGCTCGAGATTGCCGCCCAGCGCACCGTTGGCCGCACTGGCGGC 24611
13831 GGTAATAACCGTGGCGAGATAGTCCTGCAGCGTGCTCATTTGCTCGAGATTGCCGCCCAGCGCACCGTTGGCCGCACTGGCGGC 13924
GGTAATAACCGTGGCGAGATAGTCCTGCAGCGTGCTCATTTGCTCGAGATTGCCGCCCAGCGCACCGTTGGCCGCACTGGCGGC

24612 CAGACACCACCGTCGAAGACCTCGACCTGCTGGTGCCGGCAGGCGTCCATCACATCGGTAAACAGGTGCAGGACCCGGTTGTAC 24705
13925 CAGACACCACCGTCGAAGACCTCGACCTGCTGGTGCCGGCAGGCGTCCATCACATCGGTAAACAGGTGCAGGACCCGGTTGTAC 14018
CAGACACCACCGTCGAAGACCTCGACCTGCTGGTGCCGGCAGGCGTCCATCACATCGGTAAACAGGTGCAGGACCCGGTTGTAC

24706 GGTCGTAGTGGATGTCCTCGTCAGCGTCGGGCCACCCACCCGCCTCGAGCATCCGCCCGGCGTACTCCCCCGTCGGCCTCGGAA 24799
14019 GGTCGTAGTGGATGTCCTCGTCAGCGTCGGGCCACCCACCCGCCTCGAGCATCCGCCCGGCGTACTCCCCCGTCGGCCTCGGAA 14112
GGTCGTAGTGGATGTCCTCGTCAGCGTCGGGCCACCCACCCGCCTCGAGCATCCGCCCGGCGTACTCCCCCGTCGGCCTCGGAA

24800 CTACTCTCCTCCCCGCAGCATCCAGGCCGCAGCGACCAGACCTGTCAGGTAGGTACCCGCAGTGGATCGTTGGACCGGCGCGCA 24893
14113 CTACTCTCCTCCCCGCAGCATCCAGGCCGCAGCGACCAGACCTGTCAGGTAGGTACCCGCAGTGGATCGTTGGACCGGCGCGCA 14206
CTACTCTCCTCCCCGCAGCATCCAGGCCGCAGCGACCAGACCTGTCAGGTAGGTACCCGCAGTGGATCGTTGGACCGGCGCGCA

24894 CCGTTGCGCTGGCTTGCGGGCCGCGTCAGCTGGTCAGTGGTACCGGACGTCATGGTTAACCAGCCTAACCGAGGTTAAGACCAG 24987
14207 CCGTTGCGCTGGCTTGCGGGCCGCGTCAGCTGGTCAGTGGTACCGGACGTCATGGTTAACCAGCCTAACCGAGGTTAAGACCAG 14300
CCGTTGCGCTGGCTTGCGGGCCGCGTCAGCTGGTCAGTGGTACCGGACGTCATGGTTAACCAGCCTAACCGAGGTTAAGACCAG

24988 CAATTTGGGGATCGGGACGGGCCGACGGAAATAACACCACAGCACGGCAGACCGCCGTATCGGAGAGATTTCGCGCAGGGTTGA 25081
14301 CAATTTGGGGATCGGGACGGGCCGACGGAAATAACACCACAGCACGGCAGACCGCCGTATCGGAGAGATTTCGCGCAGGGTTGA 14394
CAATTTGGGGATCGGGACGGGCCGACGGAAATAACACCACAGCACGGCAGACCGCCGTATCGGAGAGATTTCGCGCAGGGTTGA

25082 GAACGACGCAGACGCCGCGACACGAGGCACCACGGCGGTGGCGTCAATGGTCCGCTAGCGCGAACCAACAGCCGCTGACAGGC 25175
14395 GAACGACGCAGACGCCGCGACACGAGGCACCACGGCGGTGGCGTCAATGGTCCGCTAGCGCGAACCAACAGCCGCTGACAGGC 14488
GAACGACGCAGACGCCGCGACACGAGGCACCACGGCGGTGGCGTCAATGGTCCGCTAGCGCGAACCAACAGCCGCTGACAGGC

25176 ACTGTTTCCACCCCGGCGCAGGGACGGATCAGACCATCCCGTTGTTCATTGTTTGAGACATTGAGGACAACACCGCGGTCAGCTT 25269
14489 ACTGTTTCCACCCCGGCGCAGGGACGGATCAGACCATCCCGTTGTTCATTGTTTGAGACATTGAGGACAACACCGCGGTCAGCTT 14582
ACTGTTTCCACCCCGGCGCAGGGACGGATCAGACCATCCCGTTGTTCATTGTTTGAGACATTGAGGACAACACCGCGGTCAGCTT

25270 CGCGTCGTTGTATTTGGATGCGGCGGCCTGGGCGTTCTTCAACGCCTGGTTGACCCGCGCACTAACGACCTCCGCACCAACTTCC 25363
14583 CGCGTCGTTGTATTTGGATGCGGCGGCCTGGGCGTTCTTCAACGCCTGGTTGACCCGCGCACTAACGACCTCCGCACCAACTTCC 14676

CGCGTCGTTGTATTTGGATGCGGCGGCCTGGGCGTTCTTCAACGCCTGGTTGACCCGCGCACTAACGACCTCCGCACCAACTTCC

25364 CCGTCGTCGATGCGCACAGCGGTGAGCCACTGGTGCCCATTGATCGTCACCTCGACCGTCTCGGTGTCGTCTTTGCCACGGAAAT 25457
14677 CCGTCGTCGATGCGCACAGCGGTGAGCCACTGGTGCCCATTGATCGTCACCTCGACCGTCTCGGTGTCGTCTTTGCCACGGAAAT 14770

CCGTCGTCGATGCGCACAGCGGTGAGCCACTGGTGCCCATTGATCGTCACCTCGACCGTCTCGGTGTCGTCTTTGCCACGGAAAT

25458 TCATTTGGTTCAGCGTTCCGTCCAAGGCCGACTGGAACCGTGCCGCCAACGCCAACACCTGCGCGACTTGGGGGTCCATCTCCAT 25551
14771 TCATTTGGTTCAGCGTTCCGTCCAAGGCCGACTGGAACCGTGCCGCCAACGCCAACACCTGCGCGACTTGGGGGTCCATCTCCAT 14864

TCATTTGGTTCAGCGTTCCGTCCAAGGCCGACTGGAACCGTGCCGCCAACGCCAACACCTGCGCGACTTGGGGGTCCATCTCCAT

25552 CTTGACTCCTTACTGTCCTGACGACTGTCCTGACGGCTACTTGTTGTCCTGACGGCGGCGGTTACCGATTACGGCCTCGGTCCAC 25645
14865 CTTGACTCCTTACTGTCCTGACGACTGTCCTGACGGCTACTTGTTGTCCTGACGGCGGCGGTTACCGATTACGGCCTCGGTCCAC 14958

CTTGACTCCTTACTGTCCTGACGACTGTCCTGACGGCTACTTGTTGTCCTGACGGCGGCGGTTACCGATTACGGCCTCGGTCCAC

25646 TCGGTGTAGAGCGCTTCCTCGTCTTGTTGAGCGCCCTTGGACTTGGCGCCGCCCTGGCCCTGGCCAGCGCCGCCCATCGGCATTC 25739
14959 TCGGTGTAGAGCGCTTCCTCGTCTTGTTGAGCGCCCTTGGACTTGGCGCCGCCCTGGCCCTGGCCAGCGCCGCCCATCGGCATTC 15052

TCGGTGTAGAGCGCTTCCTCGTCTTGTTGAGCGCCCTTGGACTTGGCGCCGCCCTGGCCCTGGCCAGCGCCGCCCATCGGCATTC

25740 CTCCGGCCATTCCGCGACCTGCGGCGCCACCACCCTGGCCGGCACCGGCGACGTCCCCTGCCGCGGCCGGTCGCACGGACTCGC 25833
15053 CTCCGGCCATTCCGCGACCTGCGGCGCCACCACCCTGGCCGGCACCGGCGACGTCCCCTGCCGCGGCCGGTCGCACGGACTCGC 15146

CTCCGGCCATTCCGCGACCTGCGGCGCCACCACCCTGGCCGGCACCGGCGACGTCCCCTGCCGCGGCCGGTCGCACGGACTCGC

25834 GGCCGCATCACCCATCGGCATCCCGCCGAGACCACCACCGCCGCCGCCCAAAGACATCGGTTTGACACCGAGGCCCTTGGACAG 25927
15147 GGCCGCATCACCCATCGGCATCCCGCCGAGACCACCACCGCCGCCGCCCAAAGACATCGGTTTGACACCGAGGCCCTTGGACAG 15240

GGCCGCATCACCCATCGGCATCCCGCCGAGACCACCACCGCCGCCGCCCAAAGACATCGGTTTGACACCGAGGCCCTTGGACAG

25928 GCCTCGCGACCCGCCGAGGTGAGCTCGGCGGTGTTGACGTCGGGTGTGCCGCCCGCTCCACCGGTGGGCGGAATCATCGGGGG 26021
15241 GCCTCGCGACCCGCCGAGGTGAGCTCGGCGGTGTTGACGTCGGGTGTGCCGCCCGCTCCACCGGTGGGCGGAATCATCGGGGG 15334

GCCTCGCGACCCGCCGAGGTGAGCTCGGCGGTGTTGACGTCGGGTGTGCCGCCCGCTCCACCGGTGGGCGGAATCATCGGGGG

26022 AGGCCAGCCCGGTGGAACCGTCGCCGGGCGGCATCAAGAAGCCCGGGATCAGGCCCTGCGGTTGCGCGGGTGGCGGCGGGTCG 26115
15335 AGGCCAGCCCGGTGGAACCGTCGCCGGGCGGCATCAAGAAGCCCGGGATCAGGCCCTGCGGTTGCGCGGGTGGCGGCGGGTCG 15428

AGGCCAGCCCGGTGGAACCGTCGCCGGGCGGCATCAAGAAGCCCGGGATCAGGCCCTGCGGTTGCGCGGGTGGCGGCGGGTCG

26116 CGCCGGAGGCTTCGGCGGATTCACCGGCTCCAGATCAGCCTTGGTGTTGTATTCGCTCAACACCTTCTCCGAGGTCTCTTGATAC 26209
15429 CGCCGGAGGCTTCGGCGGATTCACCGGCTCCAGATCAGCCTTGGTGTTGTATTCGCTCAACACCTTCTCCGAGGTCTCTTGATAC 15522
CGCCGGAGGCTTCGGCGGATTCACCGGCTCCAGATCAGCCTTGGTGTTGTATTCGCTCAACACCTTCTCCGAGGTCTCTTGATAC

26210 AGCTTGATGGCTTGCTCTTGATAGTCCGGGTCTTTCGCCAGTCTCTCGAGTTCGACGATGTCGGCCAGGGTGGGGTGCCCCCGCC 26303
15523 AGCTTGATGGCTTGCTCTTGATAGTCCGGGTCTTTCGCCAGTCTCTCGAGTTCGACGATGTCGGCCAGGGTGGGGTGCCCCCGCC 15616
AGCTTGATGGCTTGCTCTTGATAGTCCGGGTCTTTCGCCAGTCTCTCGAGTTCGACGATGTCGGCCAGGGTGGGGTGCCCCCGCC

26304 GCTGCAGTTGCGCCATGAAATTGGCCTGCTTGGCCAGCGATGCACTCAGCTTGGCCATGTGGAGTATCCACTCCTTCTGCTGGTC 26397
15617 GCTGCAGTTGCGCCATGAAATTGGCCTGCTTGGCCAGCGATGCACTCAGCTTGGCCATGTGGAGTATCCACTCCTTCTGCTGGTC 15710
GCTGCAGTTGCGCCATGAAATTGGCCTGCTTGGCCAGCGATGCACTCAGCTTGGCCATGTGGAGTATCCACTCCTTCTGCTGGTC

26398 CTCGCAGGCGGTAGCGGCGTCACCCTCCCAGTTCTCGAAGATCCGGAACCGCTTGATGTCGCGTTGCAGGGCCAGGTTGAAGTT 26491
15711 CTCGCAGGCGGTAGCGGCGTCACCCTCCCAGTTCTCGAAGATCCGGAACCGCTTGATGTCGCGTTGCAGGGCCAGGTTGAAGTT 15804
CTCGCAGGCGGTAGCGGCGTCACCCTCCCAGTTCTCGAAGATCCGGAACCGCTTGATGTCGCGTTGCAGGGCCAGGTTGAAGTT

26492 TCGGCGAAGTTGACCAACGACGTGCCCTGGTCACCGGATTCGAGCTTCGTCGCCGCGGTCTTGAGGTCGGTGAAGTCTGATTCCC 26585
15805 TCGGCGAAGTTGACCAACGACGTGCCCTGGTCACCGGATTCGAGCTTCGTCGCCGCGGTCTTGAGGTCGGTGAAGTCTGATTCCC 15898
TCGGCGAAGTTGACCAACGACGTGCCCTGGTCACCGGATTCGAGCTTCGTCGCCGCGGTCTTGAGGTCGGTGAAGTCTGATTCCC

26586 CCACCTTCGGGGTTTCCTCCAGTGAATCGGTCTGGCCGGCGCCGGCGCCGCCCGCAGATTGGGCTTCGACCTCGCCATTGCCGT 26679
15899 CCACCTTCGGGGTTTCCTCCAGTGAATCGGTCTGGCCGGCGCCGGCGCCGCCCGCAGATTGGGCTTCGACCTCGCCATTGCCGT 15992
CCACCTTCGGGGTTTCCTCCAGTGAATCGGTCTGGCCGGCGCCGGCGCCGCCCGCAGATTGGGCTTCGACCTCGCCATTGCCGT

26680 CGCGGTTGCGGACTCGTCCTCGACCTCGCCATAAGCCGCGGCGGCGTTACGCAGCGACGTCGCCAGACGTTGCCGCTCCCGCTC 26773
15993 CGCGGTTGCGGACTCGTCCTCGACCTCGCCATAAGCCGCGGCGGCGTTACGCAGCGACGTCGCCAGACGTTGCCGCTCCCGCTC 16086
CGCGGTTGCGGACTCGTCCTCGACCTCGCCATAAGCCGCGGCGGCGTTACGCAGCGACGTCGCCAGACGTTGCCGCTCCCGCTC

26774 AGATATAGGCGGACGTTGTCGGCGGAGACCGCCAGTTGTTCGGCCGCGTTGGTGGCTGCCGTGAGGCCGCACGGTGCCTGGGG 26867
16087 AGATATAGGCGGACGTTGTCGGCGGAGACCGCCAGTTGTTCGGCCGCGTTGGTGGCTGCCGTGAGGCCGCACGGTGCCTGGGG 16180
AGATATAGGCGGACGTTGTCGGCGGAGACCGCCAGTTGTTCGGCCGCGTTGGTGGCTGCCGTGAGGCCGCACGGTGCCTGGGG

26868 GCGGAGTCGCCATCGGCGCCTCCACCTCGTTGGCCCTGTTCAAGATTTCCTGCTGATCCACCGTGACGGTCTGCGGCTGGCTCAT 26961
16181 GCGGAGTCGCCATCGGCGCCTCCACCTCGTTGGCCCTGTTCAAGATTTCCTGCTGATCCACCGTGACGGTCTGCGGCTGGCTCAT 16274
GCGGAGTCGCCATCGGCGCCTCCACCTCGTTGGCCCTGTTCAAGATTTCCTGCTGATCCACCGTGACGGTCTGCGGCTGGCTCAT

26962 TCCTCCTTAGTGCTCCATGCCATTATCGTCGCTGAAACGACCGCTTGCTGCACCAAAAAATTTCGCCGCCCTCTTCCAGCCCACTT 27055
16275 TCCTCCTTAGTGCTCCATGCCATTATCGTCGCTGAAACGACCGCTTGCTGCACCAAAAAATTTCGCCGCCCTCTTCCAGCCCACTT 16368
TCCTCCTTAGTGCTCCATGCCATTATCGTCGCTGAAACGACCGCTTGCTGCACCAAAAAATTTCGCCGCCCTCTTCCAGCCCACTT

27056 TTGGGCAGGGCCATCTGGAAGCGACTTTCCTCAGGTGGAGAGACCCGCCGGATCGGCAACTTGCGGTGTCGCGGGGTGCCGACC 27149
16369 TTGGGCAGGGCCATCTGGAAGCGACTTTCCTCAGGTGGAGAGACCCGCCGGATCGGCAACTTGCGGTGTCGCGGGGTGCCGACC 16462
TTGGGCAGGGCCATCTGGAAGCGACTTTCCTCAGGTGGAGAGACCCGCCGGATCGGCAACTTGCGGTGTCGCGGGGTGCCGACC

27150 GCAGGATCACCCGATCGATACCGGGGTGTGCGCCCAGATAGGTCGTCGCGTTCGGCCACACCACCTTGGCCTCCGCACCGACAT 27243
16463 GCAGGATCACCCGATCGATACCGGGGTGTGCGCCCAGATAGGTCGTCGCGTTCGGCCACACCACCTTGGCCTCCGCACCGACAT 16556
GCAGGATCACCCGATCGATACCGGGGTGTGCGCCCAGATAGGTCGTCGCGTTCGGCCACACCACCTTGGCCTCCGCACCGACAT

27244 AAGCCCGGCGAATTGTTCGAAGTGTGGTCCCACGGTGACGACGCCCCCCGCAGCCGCCGAACGCACCGCGAACTGGGTGAAGGT 27337
16557 AAGCCCGGCGAATTGTTCGAAGTGTGGTCCCACGGTGACGACGCCCCCCGCAGCCGCCGAACGCACCGCGAACTGGGTGAAGGT 16650
AAGCCCGGCGAATTGTTCGAAGTGTGGTCCCACGGTGACGACGCCCCCCGCAGCCGCCGAACGCACCGCGAACTGGGTGAAGGT

27338 CCCAGGTTGACGGTCGCATCCACGTCATCGAACGGCATGTACACCGGGCAGCGGTTCACGGTCTCGCCGATTAACACGCCCGCT 27431
16651 CCCAGGTTGACGGTCGCATCCACGTCATCGAACGGCATGTACACCGGGCAGCGGTTCACGGTCTCGCCGATTAACACGCCCGCT 16744
CCCAGGTTGACGGTCGCATCCACGTCATCGAACGGCATGTACACCGGGCAGCGGTTCACGGTCTCGCCGATTAACACGCCCGCT

27432 GCAGTTGGCAGTGGTGATTGGCAACCAGGGTCTGCCCTTCCAACGCCGGCCGCTGACCGCCGAACAGGCGCGAGAAGCCCCGGG 27525
16745 GCAGTTGGCAGTGGTGATTGGCAACCAGGGTCTGCCCTTCCAACGCCGGCCGCTGACCGCCGAACAGGCGCGAGAAGCCCCGGG 16838
GCAGTTGGCAGTGGTGATTGGCAACCAGGGTCTGCCCTTCCAACGCCGGCCGCTGACCGCCGAACAGGCGCGAGAAGCCCCGGG

27526 CTTTCCCACTGTGGTCAGCAACACCGTGGACCGCGGCGGCATCCCGGGAGCGATCCGGACCCTGGTAATGGTGTGGTCCGCTCG 27619
16839 CTTTCCCACTGTGGTCAGCAACACCGTGGACCGCGGCGGCATCCCGGGAGCGATCCGGACCCTGGTAATGGTGTGGTCCGCTCG 16932
CTTTCCCACTGTGGTCAGCAACACCGTGGACCGCGGCGGCATCCCGGGAGCGATCCGGACCCTGGTAATGGTGTGGTCCGCTCG

27620 CACAAATCCGGGCCGCCGGGTGCTGTGTAGGCGGCGGTGTAGCTGTCGTGGCCCTTGATCATCGACCATTTCTCCCGCACATAAC 27713
16933 CACAAATCCGGGCCGCCGGGTGCTGTGTAGGCGGCGGTGTAGCTGTCGTGGCCCTTGATCATCGACCATTTCTCCCGCACATAAC 17026
CACAAATCCGGGCCGCCGGGTGCTGTGTAGGCGGCGGTGTAGCTGTCGTGGCCCTTGATCATCGACCATTTCTCCCGCACATAAC

27714 TGGCGTGGTCGTAGTCGTCGAAGCTGCGCCCGCATACCGCGTCGACGCCGTTGCTGGCCAGGCCATCGGCGATGCGAGTCGCCG 27807
17027 TGGCGTGGTCGTAGTCGTCGAAGCTGCGCCCGCATACCGCGTCGACGCCGTTGCTGGCCAGGCCATCGGCGATGCGAGTCGCCG 17120
TGGCGTGGTCGTAGTCGTCGAAGCTGCGCCCGCATACCGCGTCGACGCCGTTGCTGGCCAGGCCATCGGCGATGCGAGTCGCCG

27808 ATACCGAGCCAAACCGGCGAGGCCGGCCTCGCGGCGCTGCGCAGACTTGCGGGTTTGTTCCGGGTCGGCTCGCAGCATGATCCA 27901
17121 ATACCGAGCCAAACCGGCGAGGCCGGCCTCGCGGCGCTGCGCAGACTTGCGGGTTTGTTCCGGGTCGGCTCGCAGCATGATCCA 17214
ATACCGAGCCAAACCGGCGAGGCCGGCCTCGCGGCGCTGCGCAGACTTGCGGGTTTGTTCCGGGTCGGCTCGCAGCATGATCCA

27902 CTTGCGGGAGCCGGATCGGCGCCGATCACCCGCTGGTAAAGGCTCACCACCTCTTCGGAGGCAGTGTTGCCAACGCGGTAGCCC 27995
17215 CTTGCGGGAGCCGGATCGGCGCCGATCACCCGCTGGTAAAGGCTCACCACCTCTTCGGAGGCAGTGTTGCCAACGCGGTAGCCC 17308
CTTGCGGGAGCCGGATCGGCGCCGATCACCCGCTGGTAAAGGCTCACCACCTCTTCGGAGGCAGTGTTGCCAACGCGGTAGCCC

27996 CATCTGCTTCCAGGTCCGGGCAATGCACCGACAGCAGGTCCTCGAGCAGCCGGGTGTCCAGCACGTCGTCGGTGTGGGCCTTGC 28089
17309 CATCTGCTTCCAGGTCCGGGCAATGCACCGACAGCAGGTCCTCGAGCAGCCGGGTGTCCAGCACGTCGTCGGTGTGGGCCTTGC 17402
CATCTGCTTCCAGGTCCGGGCAATGCACCGACAGCAGGTCCTCGAGCAGCCGGGTGTCCAGCACGTCGTCGGTGTGGGCCTTGC

28090 GACCGTCGGGGTAAACGGGCGGGGTTTGAGTTCGATCACCGCAATCAACCGGTCGCGTCGCCAACGCACCGCGACGTGATCCCC 28183
17403 GACCGTCGGGGTAAACGGGCGGGGTTTGAGTTCGATCACCGCAATCAACCGGTCGCGTCGCCAACGCACCGCGACGTGATCCCC 17496
GACCGTCGGGGTAAACGGGCGGGGTTTGAGTTCGATCACCGCAATCAACCGGTCGCGTCGCCAACGCACCGCGACGTGATCCCC

28184 GTGGCACCAACCACCGGCTCGGACGGGACATCCGGCGGTCGCCGACGCCGACGCAACCAGGCGAACACGGTAGCCACCCACCCC 28277
17497 GTGGCACCAACCACCGGCTCGGACGGGACATCCGGCGGTCGCCGACGCCGACGCAACCAGGCGAACACGGTAGCCACCCACCCC 17590
GTGGCACCAACCACCGGCTCGGACGGGACATCCGGCGGTCGCCGACGCCGACGCAACCAGGCGAACACGGTAGCCACCCACCCC

28278 GACCGGCGAAAGTCACCGTCGCCACGATGACGCCCAGCGCTACCAGGGCGATGCCCACCCACCAGTAGCGCAGGTGCAGGAACA 28371
17591 GACCGGCGAAAGTCACCGTCGCCACGATGACGCCCAGCGCTACCAGGGCGATGCCCACCCACCAGTAGCGCAGGTGCAGGAACA 17684
GACCGGCGAAAGTCACCGTCGCCACGATGACGCCCAGCGCTACCAGGGCGATGCCCACCCACCAGTAGCGCAGGTGCAGGAACA

28372 GGGCGGCGCCAGCACCGCAACGACAAGTGTGTGACCGGTGCTGACCCGGAACCGTATTGAACTGAAGGGGTTCCTCATCGGCGC 28465
17685 GGGCGGCGCCAGCACCGCAACGACAAGTGTGTGACCGGTGCTGACCCGGAACCGTATTGAACTGAAGGGGTTCCTCATCGGCGC 17778
GGGCGGCGCCAGCACCGCAACGACAAGTGTGTGACCGGTGCTGACCCGGAACCGTATTGAACTGAAGGGGTTCCTCATCGGCGC

28466 GGGCGGCCAACGTGCCCAATCCGAGCACCAGCGTCAGCCCCAGTAGCGACACCGCCACCATGGTGATCGGGCGGTGATCCGGGC 28559
17779 GGGCGGCCAACGTGCCCAATCCGAGCACCAGCGTCAGCCCCAGTAGCGACACCGCCACCATGGTGATCGGGCGGTGATCCGGGC 17872
GGGCGGCCAACGTGCCCAATCCGAGCACCAGCGTCAGCCCCAGTAGCGACACCGCCACCATGGTGATCGGGCGGTGATCCGGGC

28560 GCGTGGGGGCGGTAGTCGTCTGACGCTGGGGGGCACCGACGCCGGGCCCGGCGGGATGTCCCACGTCAGCGCCGCGACGGCAT 28653
17873 GCGTGGGGGCGGTAGTCGTCTGACGCTGGGGGGCACCGACGCCGGGCCCGGCGGGATGTCCCACGTCAGCGCCGCGACGGCAT 17966
GCGTGGGGGCGGTAGTCGTCTGACGCTGGGGGGCACCGACGCCGGGCCCGGCGGGATGTCCCACGTCAGCGCCGCGACGGCAT

```
28654 GAGCCGACGAGATTGTCGATACCGCCTCCGGGATGTCTCGCGGTGGCCGTGATCCGGTTCATCACCTGCACCGGCGTCAGTTCCC 28747
17967 GAGCCGACGAGATTGTCGATACCGCCTCCGGGATGTCTCGCGGTGGCCGTGATCCGGTTCATCACCTGCACCGGCGTCAGTTCCC 18060
      GAGCCGACGAGATTGTCGATACCGCCTCCGGGATGTCTCGCGGTGGCCGTGATCCGGTTCATCACCTGCACCGGCGTCAGTTCCC

28748 GCCGGACCAGCGCGGCCAGACCCGAGACATAAGCCGCCGCAAACGAGGTGCCGGCGATCGGGACGGGTCCTTCTCGACCTTGCA 28841
18061 GCCGGACCAGCGCGGCCAGACCCGAGACATAAGCCGCCGCAAACGAGGTGCCGGCGATCGGGACGGGTCCTTCTCGACCTTGCA 18154
      GCCGGACCAGCGCGGCCAGACCCGAGACATAAGCCGCCGCAAACGAGGTGCCGGCGATCGGGACGGGTCCTTCTCGACCTTGCA

28842 CGGTTCGCCGTGATCACCGAGCGCGATGATGTTCTCCGCGGGTGCCGCTACCCCCACCCACGGTCCGTGCATGGAAAACGAACTC 28935
18155 CGGTTCGCCGTGATCACCGAGCGCGATGATGTTCTCCGCGGGTGCCGCTACCCCCACCCACGGTCCGTGCATGGAAAACGAACTC 18248
      CGGTTCGCCGTGATCACCGAGCGCGATGATGTTCTCCGCGGGTGCCGCTACCCCCACCCACGGTCCGTGCATGGAAAACGAACTC

28936 TTCTGGCCGATGCCCCCGACAGTCAGCACCAAGGGCGCATACCAAGCCGGTGTGACCACGGTCTGCACCTTGTTCCAGCCCCGGC 29029
18249 TTCTGGCCGATGCCCCCGACAGTCAGCACCAAGGGCGCATACCAAGCCGGTGTGACCACGGTCTGCACCTTGTTCCAGCCCCGGC 18342
      TTCTGGCCGATGCCCCCGACAGTCAGCACCAAGGGCGCATACCAAGCCGGTGTGACCACGGTCTGCACCTTGTTCCAGCCCCGGC

29030 GTGTCGACGCGTCGGGCATCGGATTCTGCGAGCAGTCGCCCGCCGGTGTTACCCGCGGCGACCACGACCACGGCGTTCTTGGCGT 29123
18343 GTGTCGACGCGTCGGGCATCGGATTCTGCGAGCAGTCGCCCGCCGGTGTTACCCGCGGCGACCACGACCACGGCGTTCTTGGCGT 18436
      GTGTCGACGCGTCGGGCATCGGATTCTGCGAGCAGTCGCCCGCCGGTGTTACCCGCGGCGACCACGACCACGGCGTTCTTGGCGT

29124 GTCGATGGCCGCGCCCAGACTTATTTCGTCGATCGGCCTGCTCACCTTGTAACACGCCGCCTCGCTGATGTTGATCACTCCCGCC 29217
18437 GTCGATGGCCGCGCCCAGACTTATTTCGTCGATCGGCCTGCTCACCTTGTAACACGCCGCCTCGCTGATGTTGATCACTCCCGCC 18530
      GTCGATGGCCGCGCCCAGACTTATTTCGTCGATCGGCCTGCTCACCTTGTAACACGCCGCCTCGCTGATGTTGATCACTCCCGCC

29218 GCCGCATGCACCACCGCACGGGCAAGACTGCGGATGGACCCGGCCGCCGGCGTCGCGTTGGGATCATTGGGGTTGGGTTGGGAC 29311
18531 GCCGCATGCACCACCGCACGGGCAAGACTGCGGATGGACCCGGCCGCCGGCGTCGCGTTGGGATCATTGGGGTTGGGTTGGGAC 18624
      GCCGCATGCACCACCGCACGGGCAAGACTGCGGATGGACCCGGCCGCCGGCGTCGCGTTGGGATCATTGGGGTTGGGTTGGGAC

29312 CGAAGGCCTCCGATGTCTGGCGCAACGAAAGCAGTCGCACGTCGGGGGCAACCCCGACGAAACCGTCAGTCGGTGCGGGGCGCC 29405
18625 CGAAGGCCTCCGATGTCTGGCGCAACGAAAGCAGTCGCACGTCGGGGGCAACCCCGACGAAACCGTCAGTCGGTGCGGGGCGCC 18718
      CGAAGGCCTCCGATGTCTGGCGCAACGAAAGCAGTCGCACGTCGGGGGCAACCCCGACGAAACCGTCAGTCGGTGCGGGGCGCC

29406 CGAGGCCGTGAGCGTGCCGTGCGCATCACAGTCCGACAGTCCGTCGCCCGCCTGATCGACGAAGTCCCCGCCCGGCTCGGCCGG 29499
18719 CGAGGCCGTGAGCGTGCCGTGCGCATCACAGTCCGACAGTCCGTCGCCCGCCTGATCGACGAAGTCCCCGCCCGGCTCGGCCGG 18812
      CGAGGCCGTGAGCGTGCCGTGCGCATCACAGTCCGACAGTCCGTCGCCCGCCTGATCGACGAAGTCCCCGCCCGGCTCGGCCGG
```

29500 GAGGCATCCACGCCGGTATCGATCACCGCCACCGTGACGCCGGCCCCGGTCGCAAACTTGTGGGCCTCTCCGACACCCATATAGC 29593
18813 GAGGCATCCACGCCGGTATCGATCACCGCCACCGTGACGCCGGCCCCGGTCGCAAACTTGTGGGCCTCTCCGACACCCATATAGC 18906

GAGGCATCCACGCCGGTATCGATCACCGCCACCGTGACGCCGGCCCCGGTCGCAAACTTGTGGGCCTCTCCGACACCCATATAGC

29594 ACGGCGGATCGTGAAAGCTGGAATCGGGCAGCGTCGTGGGGCTGGTACAGAGCACTCTCTGCTCGGTGGGTTGGTCGGGACCGC 29687
18907 ACGGCGGATCGTGAAAGCTGGAATCGGGCAGCGTCGTGGGGCTGGTACAGAGCACTCTCTGCTCGGTGGGTTGGTCGGGACCGC 19000

ACGGCGGATCGTGAAAGCTGGAATCGGGCAGCGTCGTGGGGCTGGTACAGAGCACTCTCTGCTCGGTGGGTTGGTCGGGACCGC

29688 CGGTACCGCGCCGGGATCAATCGAGGGAGGATCGATTGCCAAAGCCGGCGGTGCGGTCAGCAACGCCAGCGCGACCATCGTCAC 29781
19001 CGGTACCGCGCCGGGATCAATCGAGGGAGGATCGATTGCCAAAGCCGGCGGTGCGGTCAGCAACGCCAGCGCGACCATCGTCAC 19094

CGGTACCGCGCCGGGATCAATCGAGGGAGGATCGATTGCCAAAGCCGGCGGTGCGGTCAGCAACGCCAGCGCGACCATCGTCAC

29782 TGCACCCCGGAATCACTCCGTTCTCTAGCCGCAGTGAAGCTCTGGCACGCTCGTGTCAGTCGTGCCTGCATTTTAGACGTAACGG 29875
19095 TGCACCCCGGAATCACTCCGTTCTCTAGCCGCAGTGAAGCTCTGGCACGCTCGTGTCAGTCGTGCCTGCATTTTAGACGTAACGG 19188

TGCACCCCGGAATCACTCCGTTCTCTAGCCGCAGTGAAGCTCTGGCACGCTCGTGTCAGTCGTGCCTGCATTTTAGACGTAACGG

29876 CGACGCTATTCAACCCGGTGCCAGCCTGTCCATCCTGAACTGTGACCAAGCGGCGTCGGCAACTGGCTCCGCACGCCGTGCCGTC 29969
19189 CGACGCTATTCAACCCGGTGCCAGCCTGTCCATCCTGAACTGTGACCAAGCGGCGTCGGCAACTGGCTCCGCACGCCGTGCCGTC 19282

CGACGCTATTCAACCCGGTGCCAGCCTGTCCATCCTGAACTGTGACCAAGCGGCGTCGGCAACTGGCTCCGCACGCCGTGCCGTC

29970 GCGTCGCTCAAAGCTGCACGAACACATGCCATTCCACGCGCCCGGGCTTACCCTCGCCAACCGGCGCGACCCGTCACCAAACGAT 30063
19283 GCGTCGCTCAAAGCTGCACGAACACATGCCATTCCACGCGCCCGGGCTTACCCTCGCCAACCGGCGCGACCCGTCACCAAACGAT 19376

GCGTCGCTCAAAGCTGCACGAACACATGCCATTCCACGCGCCCGGGCTTACCCTCGCCAACCGGCGCGACCCGTCACCAAACGAT

30064 GTCACGGTTGTCCGCGCACACGCTGCGCACCGCCGCTTCGACATCACCAGCGGCGATCATCTGAAGATCCTCGACGGACACCGCT 30157
19377 GTCACGGTTGTCCGCGCACACGCTGCGCACCGCCGCTTCGACATCACCAGCGGCGATCATCTGAAGATCCTCGACGGACACCGCT 19470

GTCACGGTTGTCCGCGCACACGCTGCGCACCGCCGCTTCGACATCACCAGCGGCGATCATCTGAAGATCCTCGACGGACACCGCT

30158 CGCTTCTGGGCGACCACTCGAGTGTCCCGATATCCCTCGGCGCGCTCGATGACGTTGCGCGCGAACCGCCCGTTCTGCATGGCAT 30251
19471 CGCTTCTGGGCGACCACTCGAGTGTCCCGATATCCCTCGGCGCGCTCGATGACGTTGCGCGCGAACCGCCCGTTCTGCATGGCAT 19564

CGCTTCTGGGCGACCACTCGAGTGTCCCGATATCCCTCGGCGCGCTCGATGACGTTGCGCGCGAACCGCCCGTTCTGCATGGCAT

30252 GTTGCCCACCCGGGGTGGTGTAGTTGCGGATGGTGGTGGCCGCATCCAAAAAGGTCTCGCGCGCCGCCTCATCGAGCAGGCTGG 30345
19565 GTTGCCCACCCGGGGTGGTGTAGTTGCGGATGGTGGTGGCCGCATCCAAAAAGGTCTCGCGCGCCGCCTCATCGAGCAGGCTGG 19658

GTTGCCCACCCGGGGTGGTGTAGTTGCGGATGGTGGTGGCCGCATCCAAAAAGGTCTCGCGCGCCGCCTCATCGAGCAGGCTGG

30346 GGCATAGCGCTGCCCGATCTCGACGATTTCCGTGGGCGAATAGGACTCAAAGCGCAGCTTGCGATTGAATCGGCCGGCCAAACC[illegible] 30439
19659 GGCATAGCGCTGCCCGATCTCGACGATTTCCGTGGGCGAATAGGACTCAAAGCGCAGCTTGCGATTGAATCGGCCGGCCAAACC[illegible] 19752

GGCATAGCGCTGCCCGATCTCGACGATTTCCGTGGGCGAATAGGACTCAAAGCGCAGCTTGCGATTGAATCGGCCGGCCAAACC[illegible]

30440 CTGAGGAATTCGTCGACCTGATCCTCATAGCCCGCACCGATGAAACAGAAATCGAATCGGTGGGTCTCCAACTTGACCAGGAGCT 30533
19753 CTGAGGAATTCGTCGACCTGATCCTCATAGCCCGCACCGATGAAACAGAAATCGAATCGGTGGGTCTCCAACTTGACCAGGAGCT 19846

CTGAGGAATTCGTCGACCTGATCCTCATAGCCCGCACCGATGAAACAGAAATCGAATCGGTGGGTCTCCAACTTGACCAGGAGCT

30534 CCTCCATGCCAATCATGTCCGGTGTTCCGTCCTGGTGACGTTCCACCAGTGAGTAAAACTCGTGTTTCATCCGCACCGAGAAGCG[illegible] 30627
19847 CCTCCATGCCAATCATGTCCGGTGTTCCGTCCTGGTGACGTTCCACCAGTGAGTAAAACTCGTGTTTCATCCGCACCGAGAAGCG[illegible] 19940

CCTCCATGCCAATCATGTCCGGTGTTCCGTCCTGGTGACGTTCCACCAGTGAGTAAAACTCGTGTTTCATCCGCACCGAGAAGCG[illegible]

30628 CGCACGCAGCCTGCGGTTGTAGAAGAGGTGCCCGGAGTTTTGGTCCCGGTTGGACAAACTCAATGGCATTGCTCAGTACCCCCTT 30721
19941 CGCACGCAGCCTGCGGTTGTAGAAGAGGTGCCCGGAGTTTTGGTCCCGGTTGGACAAACTCAATGGCATTGCTCAGTACCCCCTT 20034

CGCACGCAGCCTGCGGTTGTAGAAGAGGTGCCCGGAGTTTTGGTCCCGGTTGGACAAACTCAATGGCATTGCTCAGTACCCCCTT

30722 CGTGGATCGGCTTGCTGGATCCGATCAGCCAGGTTCGATGCATCAGCGGCAACCCCGTAGCGGCCCTGCGCGTAGTTGATGAAC[illegible] 30815
20035 CGTGGATCGGCTTGCTGGATCCGATCAGCCAGGTTCGATGCATCAGCGGCAACCCCGTAGCGGCCCTGCGCGTAGTTGATGAAC[illegible] 20128

CGTGGATCGGCTTGCTGGATCCGATCAGCCAGGTTCGATGCATCAGCGGCAACCCCGTAGCGGCCCTGCGCGTAGTTGATGAAC[illegible]

30816 GGGCCACCAGGTTGGTGATGTTGGTCGAGGTGCCCGGCTGGTGGTAGGCCGCGAAGGTCGGTGCGATGAACTGCCACGCACCCC[illegible] 30909
20129 GGGCCACCAGGTTGGTGATGTTGGTCGAGGTGCCCGGCTGGTGGTAGGCCGCGAAGGTCGGTGCGATGAACTGCCACGCACCCC[illegible] 20222

GGGCCACCAGGTTGGTGATGTTGGTCGAGGTGCCCGGCTGGTGGTAGGCCGCGAAGGTCGGTGCGATGAACTGCCACGCACCCC[illegible]

30910 GCCCTGAGACGCGTTAGCGTCCCAGTCGTTCACCGCGCTGGCGTTGTAGCCCGACTCTCGGCGGGCCACCAAATCCATCCCGCG[illegible] 31003
20223 GCCCTGAGACGCGTTAGCGTCCCAGTCGTTCACCGCGCTGGCGTTGTAGCCCGACTCTCGGCGGGCCACCAAATCCATCCCGCG[illegible] 20316

GCCCTGAGACGCGTTAGCGTCCCAGTCGTTCACCGCGCTGGCGTTGTAGCCCGACTCTCGGCGGGCCACCAAATCCATCCCGCG[illegible]

31004 GCTCGTGCTGCAGGGTCGTGAATACCCTTGATATCGAGGGCTTTACGGATCGCCGCCAACACAGCGGGCCGGCCGGTGGGTGGA 31097
20317 GCTCGTGCTGCAGGGTCGTGAATACCCTTGATATCGAGGGCTTTACGGATCGCCGCCAACACAGCGGGCCGGCCGGTGGGTGGA 20410

GCTCGTGCTGCAGGGTCGTGAATACCCTTGATATCGAGGGCTTTACGGATCGCCGCCAACACAGCGGGCCGGCCGGTGGGTGGA

31098 GCTGTCGCCTAGCGGCTGCCTGTAGGTACCGCAGCCGGCGCAGCCGCAACACCAGTTGTCGCGCCCGTGCACGCGACCGCACGA 31191
20411 GCTGTCGCCTAGCGGCTGCCTGTAGGTACCGCAGCCGGCGCAGCCGCAACACCAGTTGTCGCGCCCGTGCACGCGACCGCACGA 20504

GCTGTCGCCTAGCGGCTGCCTGTAGGTACCGCAGCCGGCGCAGCCGCAACACCAGTTGTCGCGCCCGTGCACGCGACCGCACGA

31192 CTGGGCTCGCAGTCTTGCCGCCATGCGGACCATCGCTTCGCGCCGGCCAAGCGGCGTATCGGCCGCGAGCGCGCTATCGGCGTG 31285
20505 CTGGGCTCGCAGTCTTGCCGCCATGCGGACCATCGCTTCGCGCCGGCCAAGCGGCGTATCGGCCGCGAGCGCGCTATCGGCGTG 20598
CTGGGCTCGCAGTCTTGCCGCCATGCGGACCATCGCTTCGCGCCGGCCAAGCGGCGTATCGGCCGCGAGCGCGCTATCGGCGTG

31286 AGGATGGCGCGCGTGGCCCTTCTCGCATGCGCGTGATCGGCCTGGGCTATCGACATGATCTGTGCGAATTCCCGGTCGGTGTCG 31379
20599 AGGATGGCGCGCGTGGCCCTTCTCGCATGCGCGTGATCGGCCTGGGCTATCGACATGATCTGTGCGAATTCCCGGTCGGTGTCG 20692
AGGATGGCGCGCGTGGCCCTTCTCGCATGCGCGTGATCGGCCTGGGCTATCGACATGATCTGTGCGAATTCCCGGTCGGTGTCG

31380 GTACGGCCTGGACGACGGCGTGCGATCGCGCTGCGGCCCGGTCGGGAAGCCGGTTCCCTCCTGTCTGCGATACGGAGTCGGCCA 31473
20693 GTACGGCCTGGACGACGGCGTGCGATCGCGCTGCGGCCCGGTCGGGAAGCCGGTTCCCTCCTGTCTGCGATACGGAGTCGGCCA 20786
GTACGGCCTGGACGACGGCGTGCGATCGCGCTGCGGCCCGGTCGGGAAGCCGGTTCCCTCCTGTCTGCGATACGGAGTCGGCCA

31474 CTGCGGCTCGTGGCGGCTTCTTTCGTCGACTAAGCGACCGGCGAACAGCCCGTGCCCGCGGGACAAGGTCGCCAGCGTCTGAAG 31567
20787 CTGCGGCTCGTGGCGGCTTCTTTCGTCGACTAAGCGACCGGCGAACAGCCCGTGCCCGCGGGACAAGGTCGCCAGCGTCTGAAG 20880
CTGCGGCTCGTGGCGGCTTCTTTCGTCGACTAAGCGACCGGCGAACAGCCCGTGCCCGCGGGACAAGGTCGCCAGCGTCTGAAG

31568 TCAGTCAAGAATTCCTCCGCCGCCGTGCCGAAACCGGCCCACGTCTGTCAGCAGACCACTCCGCCTGGAAACAAACACCCAAGCC 31661
20881 TCAGTCAAGAATTCCTCCGCCGCCGTGCCGAAACCGGCCCACGTCTGTCAGCAGACCACTCCGCCTGGAAACAAACACCCAAGCC 20974
TCAGTCAAGAATTCCTCCGCCGCCGTGCCGAAACCGGCCCACGTCTGTCAGCAGACCACTCCGCCTGGAAACAAACACCCAAGCC

31662 GTACCTCCGGGTCAGCGAAACGCGGCGCACCATGGGCGCGGACACCGTCGCCCGCCCATCGCAGGGTAGCTGCAGCGCATTTGC 31755
20975 GTACCTCCGGGTCAGCGAAACGCGGCGCACCATGGGCGCGGACACCGTCGCCCGCCCATCGCAGGGTAGCTGCAGCGCATTTGC 21068
GTACCTCCGGGTCAGCGAAACGCGGCGCACCATGGGCGCGGACACCGTCGCCCGCCCATCGCAGGGTAGCTGCAGCGCATTTGC

31756 CGATTGACCGGGCCACCATCGGTAATGGCGACACCCGTGACGAGATTACTTCGGCTCGGAGGAGCCGACTAGTCACCTAACCAC 31849
21069 CGATTGACCGGGCCACCATCGGTAATGGCGACACCCGTGACGAGATTACTTCGGCTCGGAGGAGCCGACTAGTCACCTAACCAC 21162
CGATTGACCGGGCCACCATCGGTAATGGCGACACCCGTGACGAGATTACTTCGGCTCGGAGGAGCCGACTAGTCACCTAACCAC

31850 ACGACTGACCAGGTTGCTAAGAGGTCGCCGGCGACACGTGAGAAAGGTCATTTCGCGGAACGCACGTCTTCCTGGGTGCGCTGC 31943
21163 ACGACTGACCAGGTTGCTAAGAGGTCGCCGGCGACACGTGAGAAAGGTCATTTCGCGGAACGCACGTCTTCCTGGGTGCGCTGC 21256
ACGACTGACCAGGTTGCTAAGAGGTCGCCGGCGACACGTGAGAAAGGTCATTTCGCGGAACGCACGTCTTCCTGGGTGCGCTGC

31944 GGGGACCATCCCGCGCGTTGCTCCCAACTGGGCAAGCATGAACTCATGCCGGCACGCACCTTCCGATATCTGGGCGTAGTCGGC 32037
21257 GGGGACCATCCCGCGCGTTGCTCCCAACTGGGCAAGCATGAACTCATGCCGGCACGCACCTTCCGATATCTGGGCGTAGTCGGC 21350
GGGGACCATCCCGCGCGTTGCTCCCAACTGGGCAAGCATGAACTCATGCCGGCACGCACCTTCCGATATCTGGGCGTAGTCGGC

```
32038 GGCCAGAAACGATGTCCGCCGAGCTACTGTCACTAGTCCTCGGCGGCTACCAACTAATGAATGCGTTCAACGTTGCGCGAATTCG 32131
21351 GGCCAGAAACGATGTCCGCCGAGCTACTGTCACTAGTCCTCGGCGGCTACCAACTAATGAATGCGTTCAACGTTGCGCGAATTCG 21444
      GGCCAGAAACGATGTCCGCCGAGCTACTGTCACTAGTCCTCGGCGGCTACCAACTAATGAATGCGTTCAACGTTGCGCGAATTCG

32132 ATTTGGATGCTCTCGGATGCCCGGGGACGGACCTCATAAGGCAATGCACCCTGCCCTTCATCGGATGACCATCGCCACCGCGGTG 32225
21445 ATTTGGATGCTCTCGGATGCCCGGGGACGGACCTCATAAGGCAATGCACCCTGCCCTTCATCGGATGACCATCGCCACCGCGGTG 21538
      ATTTGGATGCTCTCGGATGCCCGGGGACGGACCTCATAAGGCAATGCACCCTGCCCTTCATCGGATGACCATCGCCACCGCGGTG

32226 CTCGACAAGTGGGGCGCAAACGGGCGGTCAGCGCTGAGAGTGAATTGACGCGCCGACGCACGGTAGTAATGTTGCCGCCGATCG 32319
21539 CTCGACAAGTGGGGCGCAAACGGGCGGTCAGCGCTGAGAGTGAATTGACGCGCCGACGCACGGTAGTAATGTTGCCGCCGATCG 21632
      CTCGACAAGTGGGGCGCAAACGGGCGGTCAGCGCTGAGAGTGAATTGACGCGCCGACGCACGGTAGTAATGTTGCCGCCGATCG

32320 AAGGGCCGTTCGTAGCATGATGTCGTGCCAGGAAGCAGGTGCGCTTGGCTACAACGTCACATTGCAGGGACGTAGCTGCTGGAA 32413
21633 AAGGGCCGTTCGTAGCATGATGTCGTGCCAGGAAGCAGGTGCGCTTGGCTACAACGTCACATTGCAGGGACGTAGCTGCTGGAA 21726
      AAGGGCCGTTCGTAGCATGATGTCGTGCCAGGAAGCAGGTGCGCTTGGCTACAACGTCACATTGCAGGGACGTAGCTGCTGGAA

32414 ACAACACCGCGAACGCAGCTGGTAGCACTCGTCGGATAGCCCAGTGCACCTTCGACATATAAGCCAGGCT 32483
21727 ACAACACCGCGAACGCAGCTGGTAGCACTCGTCGGATAGCCCAGTGCACCTTCGACATATAAGC------ 21790
      ACAACACCGCGAACGCAGCTGGTAGCACTCGTCGGATAGCCCAGTGCACCTTCGACATATAAGCCAGGCT
```

Fig S2B

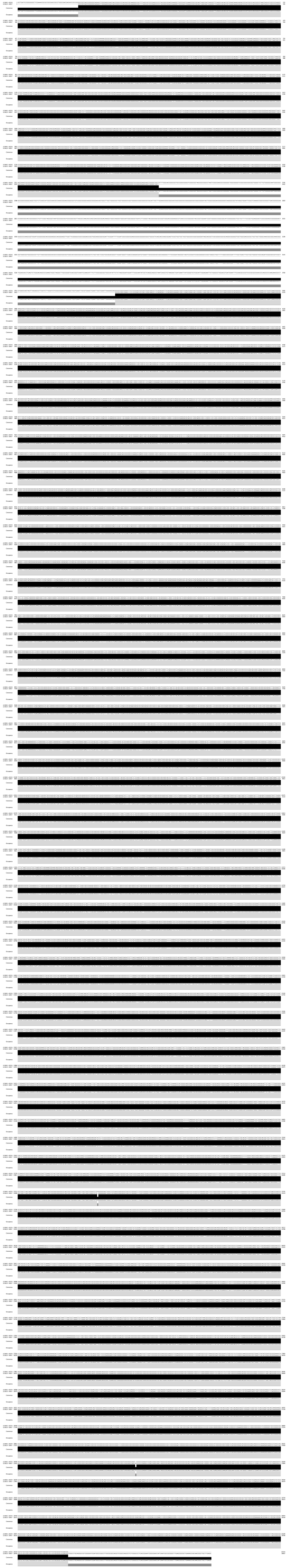

Figure S2C

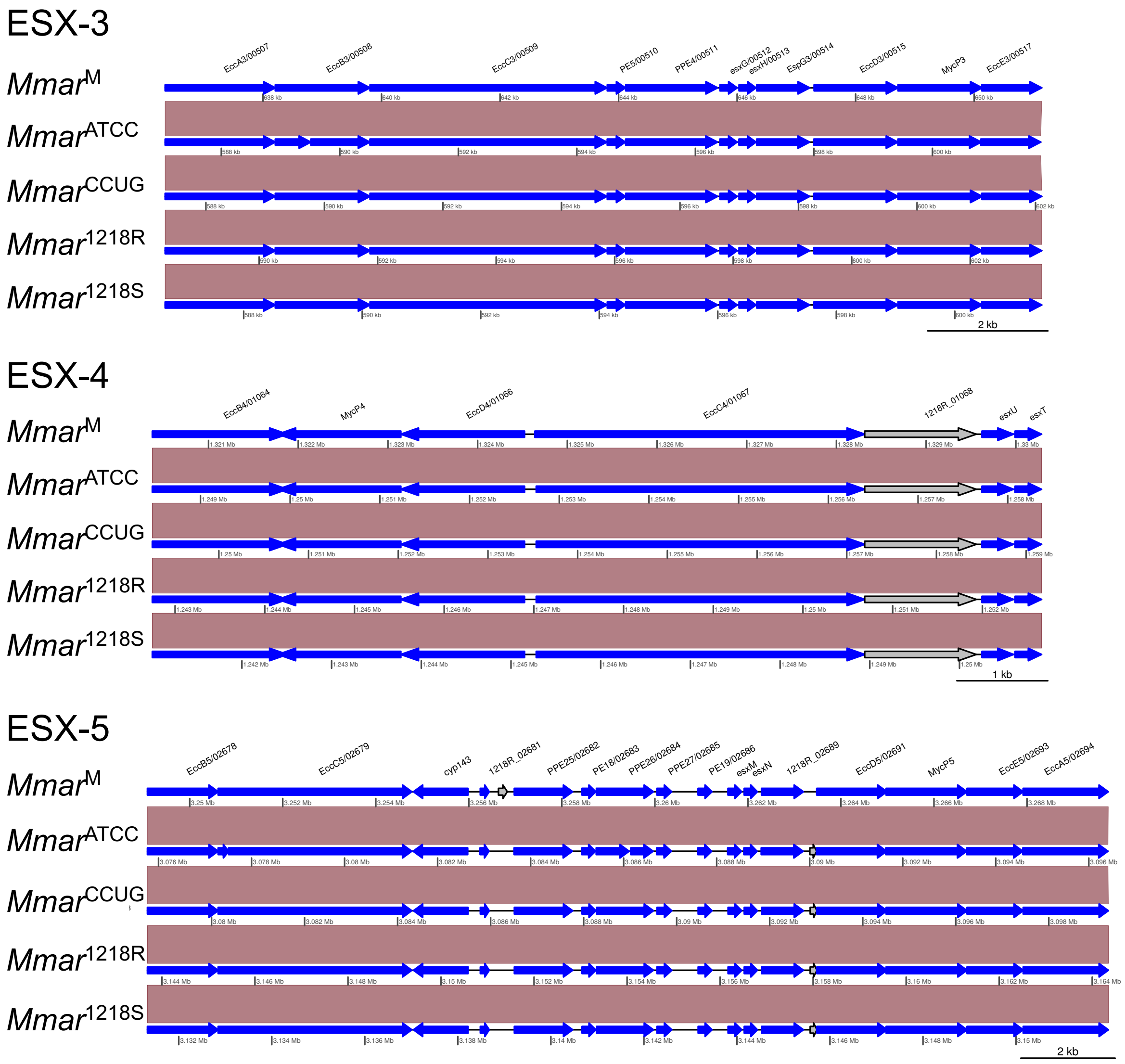
ESX-3
Mmar^M
Mmar^ATCC
Mmar^CCUG
Mmar^1218R
Mmar^1218S
EccA3/00507
EccB3/00508
EccC3/00509
PE5/00510
PPE4/00511
esxG/00512
esxH/00513
EspG3/00514
EccD3/00515
MycP3
EccE3/00517
2 kb
ESX-4
EccB4/01064
MycP4
EccD4/01066
EccC4/01067
1218R_01068
esxU
esxT
1 kb
ESX-5
EccB5/02678
EccC5/02679
cyp143
1218R_02681
PPE25/02682
PE18/02683
PPE26/02684
PPE27/02685
PE19/02686
esxM
esxN
1218R_02689
EccD5/02691
MycP5
EccE5/02693
EccA5/02694
2 kb

Fig S2D

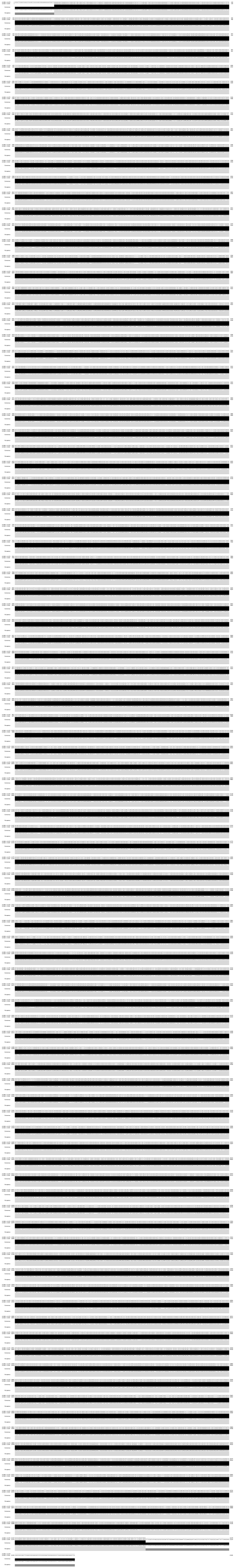

Fig S2E

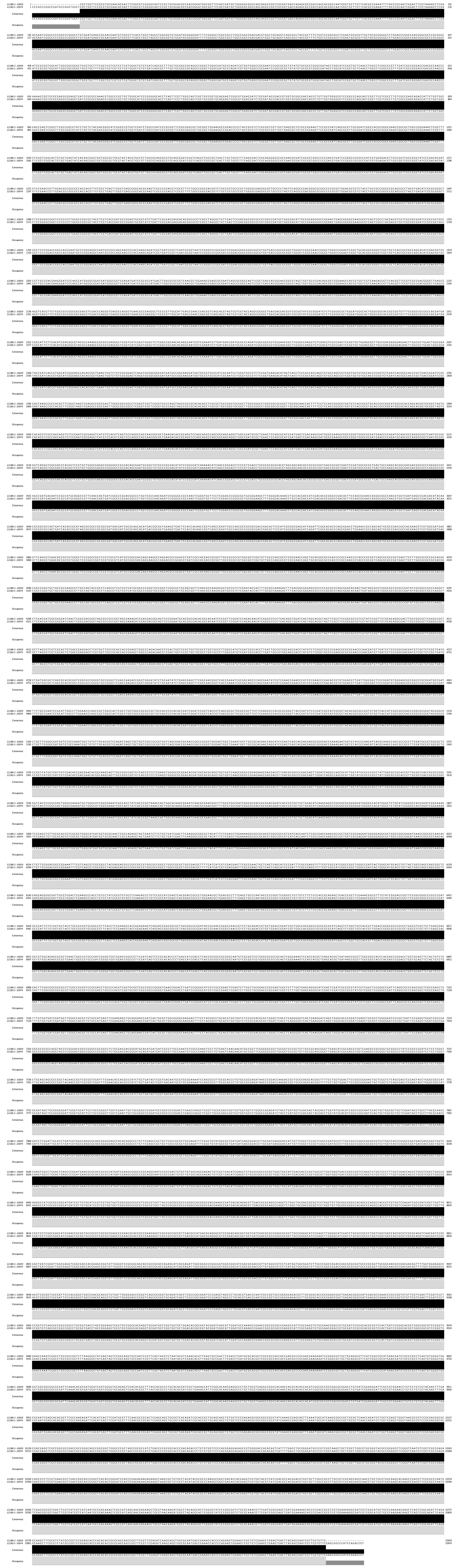

Fig S2F

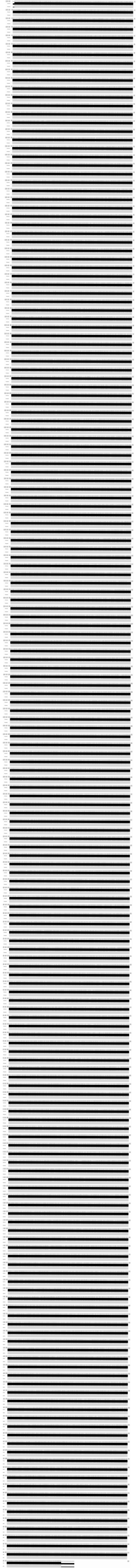

Figure S3 Illustration of gene differences/annotation for selected genes in 1218R and 1218S.

(a) Gene synteny and annotation of the RNase J gene.

(b) Gene synteny and annotation of *ppsB*_3 (and *ppsB*_1).

(c) Gene synteny reveals deletion of genes in 1218S compared to 1218R.

For further details see Table S1a-d.

Figure S3

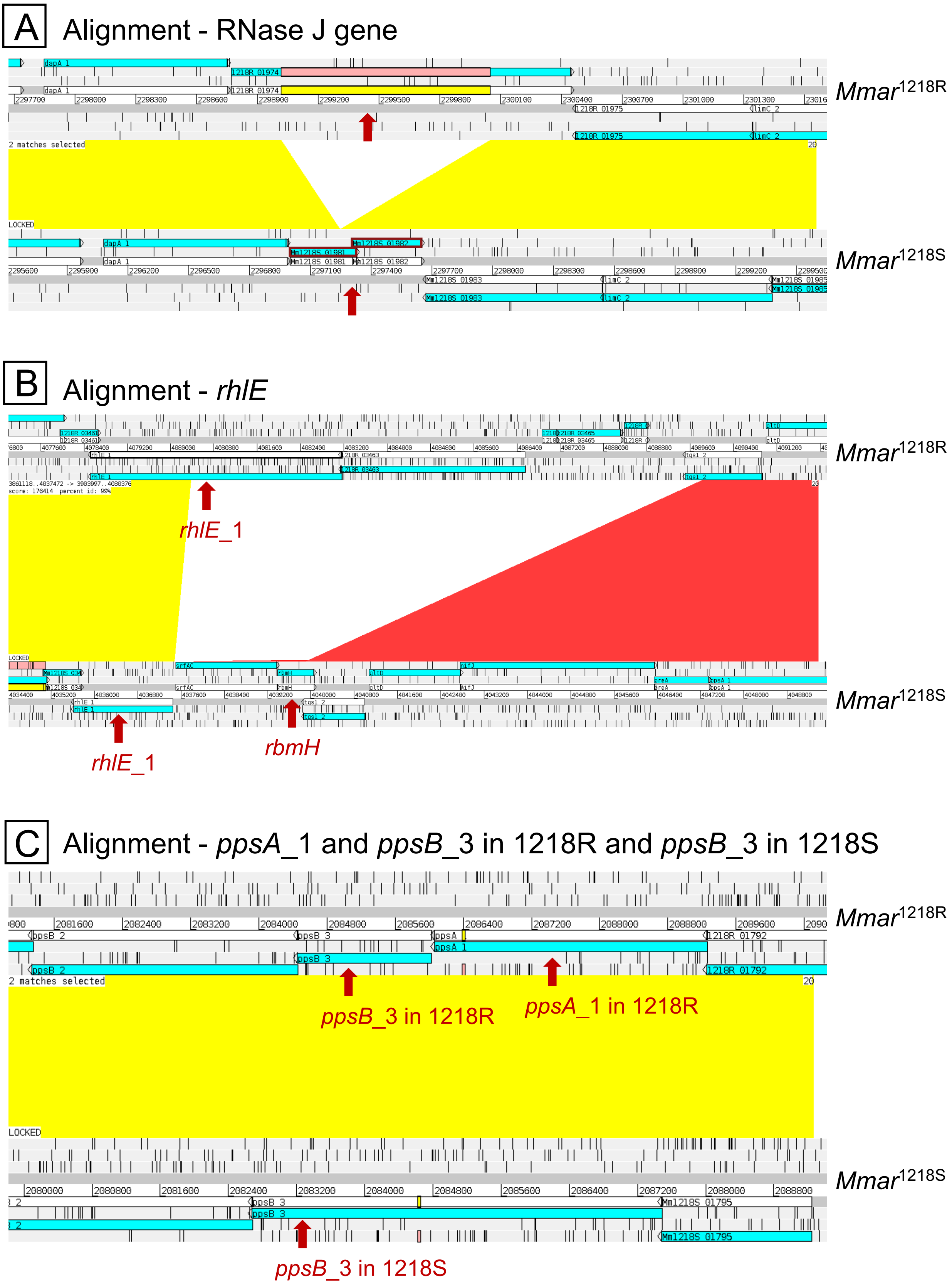
A
Alignment - RNase J gene
Mmar1218R
Mmar1218S
B
Alignment - rhlE
Mmar1218R
rhlE_1
rhlE_1
rbmH
Mmar1218S
C
Alignment - ppsA_1 and ppsB_3 in 1218R and ppsB_3 in 1218S
Mmar1218R
ppsB_3 in 1218R
ppsA_1 in 1218R
Mmar1218S
ppsB_3 in 1218S

Figure S4 Identification and classification of virulence-related genes in 1218R, 1218S, *Mmar*^{ATCC927}, *Mmar*^{CCUG} and *Mmar*^{M} using the VFanalyzer tool (the VF data base, VFDB; 333, 325, 250, 325 and 345 correspond to the total number of classified annotated virulence-related genes).

Figure S4

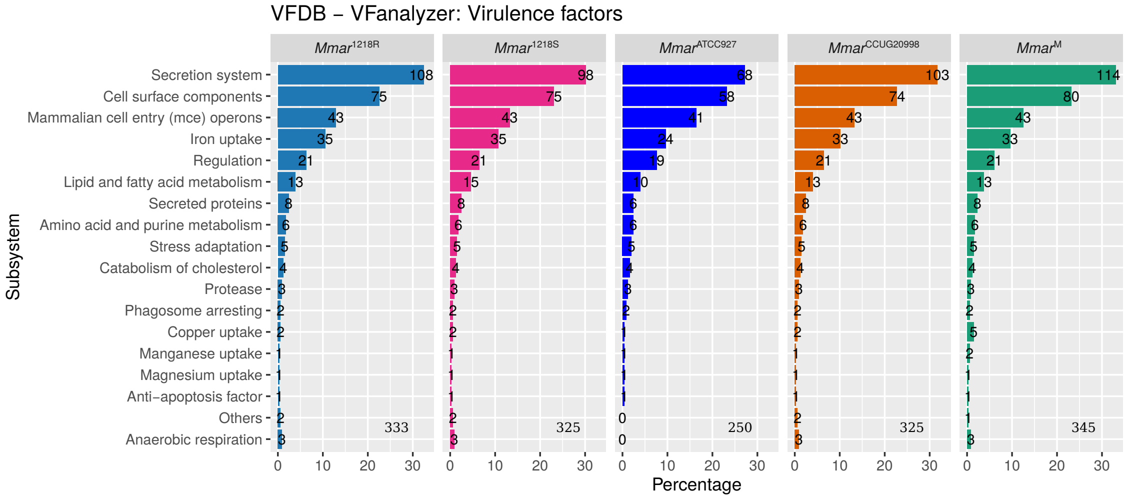

VFDB – VFanalyzer: Virulence factors
Mmar1218R
Mmar1218S
MmarATCC927
MmarCCUG20998
MmarM
Subsystem
Secretion system
Cell surface components
Mammalian cell entry (mce) operons
Iron uptake
Regulation
Lipid and fatty acid metabolism
Secreted proteins
Amino acid and purine metabolism
Stress adaptation
Catabolism of cholesterol
Protease
Phagosome arresting
Copper uptake
Manganese uptake
Magnesium uptake
Anti–apoptosis factor
Others
Anaerobic respiration
333
325
250
325
345
Percentage

Figure S5 Analysis of LOS and related regulatory genes in 1218R and 1218S.

(a) Sequence alignment of LOS genes, *mutA* to *ileS*.

(b) Sequence alignment of *lsr2*.

(c) Sequence alignment of *whiB*4.

(d) Sequence alignment of *pknL*.

Fig S5A

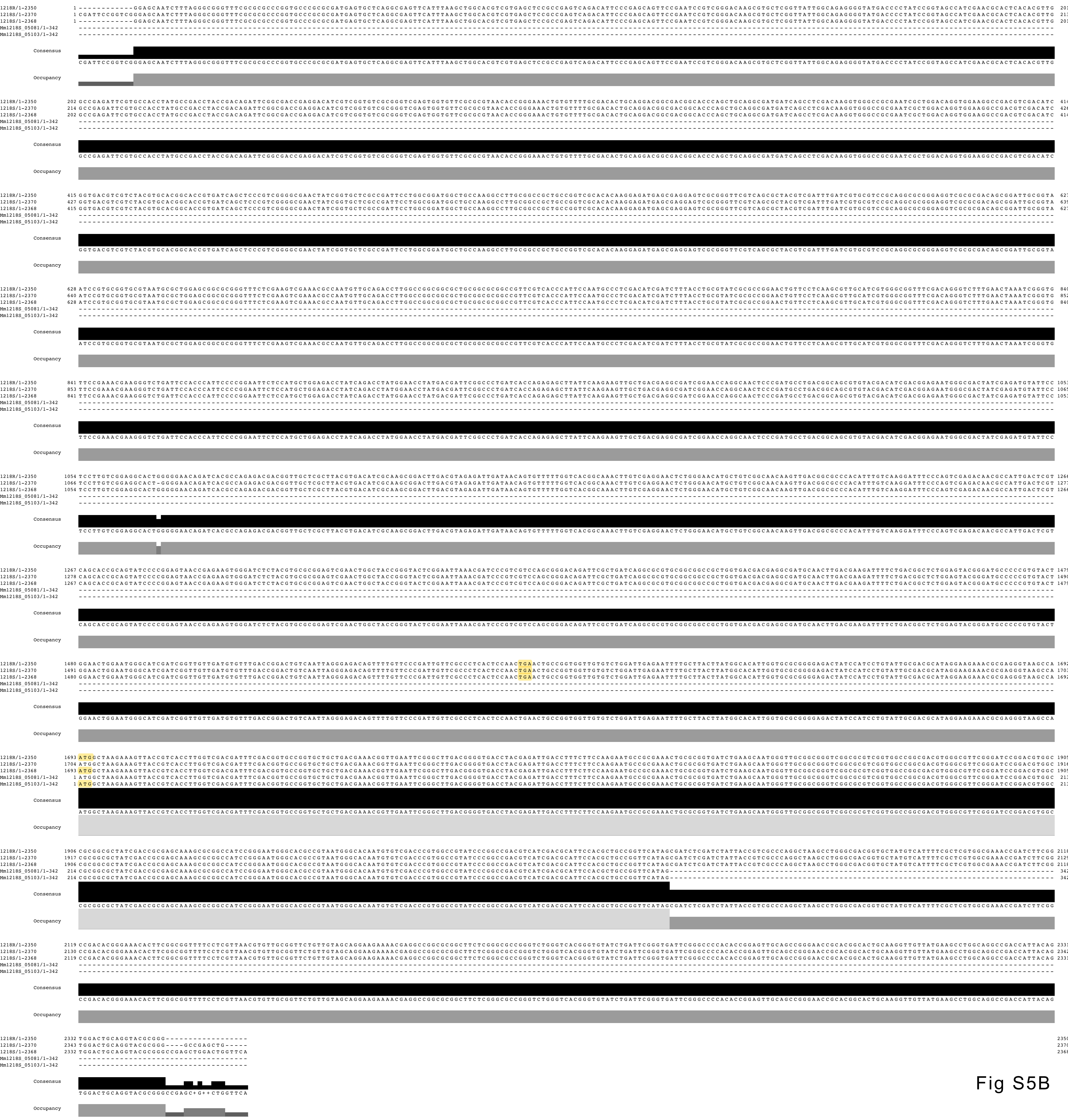
1218R/1-2350
1218S/1-2370
1218S/1-2368
Mm1218S_05081/1-342
Mm1218S_05103/1-342
Consensus
Occupancy

Fig S5B

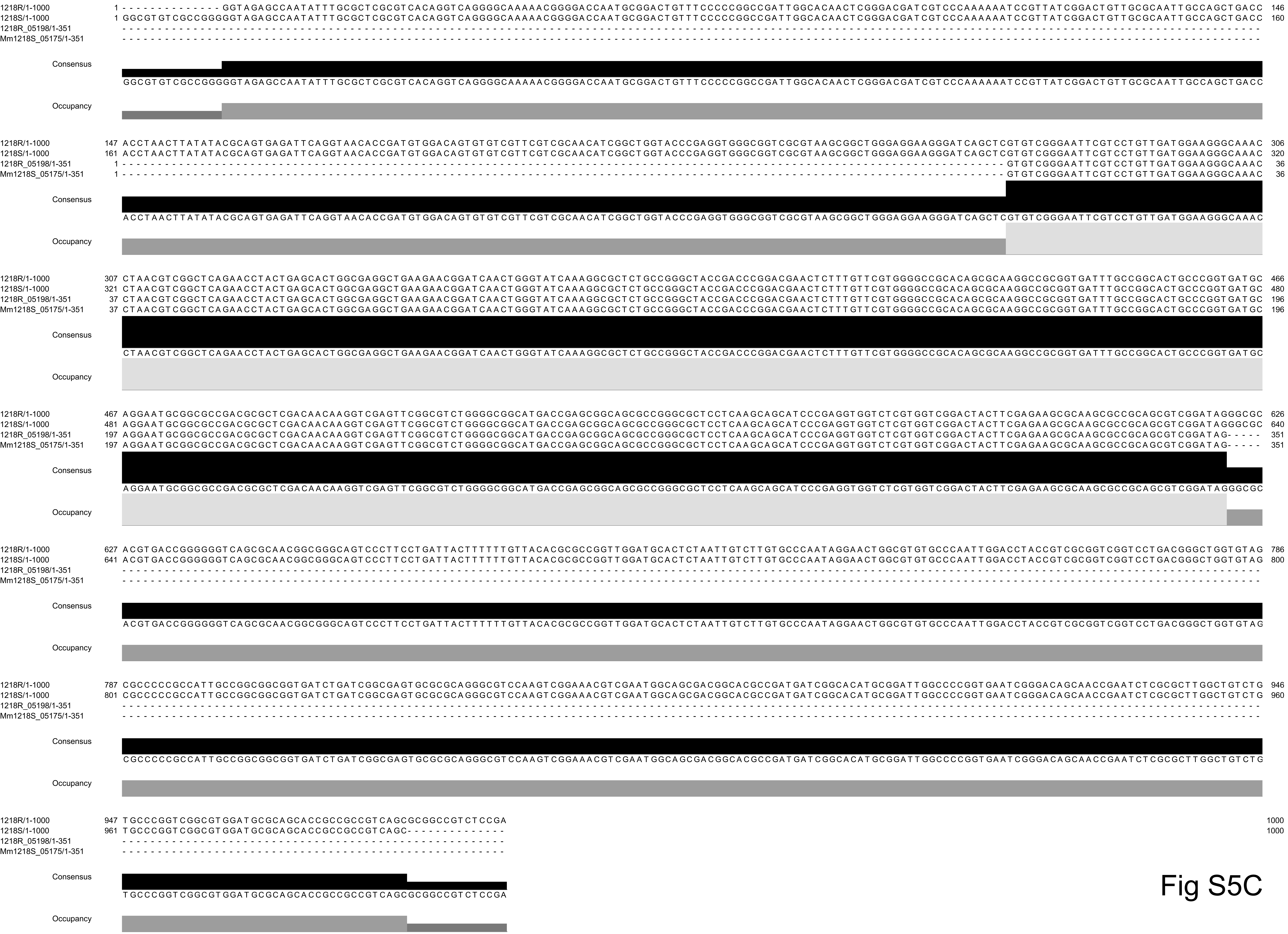
1218R/1-1000 1 --------------GGTAGAGCCAATATTTGCGCTCGCGTCACAGGTCAGGGGCAAAAACGGGGACCAATGCGGACTGTTTCCCCCGGCCGATTGGCACAACTCGGGACGATCGTCCCAAAAAATCCGTTATCGGACTGTTGCGCAATTGCCAGCTGACC 146
1218S/1-1000 1 GGCGTGTCGCCGGGGGTAGAGCCAATATTTGCGCTCGCGTCACAGGTCAGGGGCAAAAACGGGGACCAATGCGGACTGTTTCCCCCGGCCGATTGGCACAACTCGGGACGATCGTCCCAAAAAATCCGTTATCGGACTGTTGCGCAATTGCCAGCTGACC 160
1218R_05198/1-351
Mm1218S_05175/1-351
Consensus
GGCGTGTCGCCGGGGGTAGAGCCAATATTTGCGCTCGCGTCACAGGTCAGGGGCAAAAACGGGGACCAATGCGGACTGTTTCCCCCGGCCGATTGGCACAACTCGGGACGATCGTCCCAAAAAATCCGTTATCGGACTGTTGCGCAATTGCCAGCTGACC
Occupancy
1218R/1-1000 147 ACCTAACTTATATACGCAGTGAGATTCAGGTAACACCGATGTGGACAGTGTGTCGTTCGTCGCAACATCGGCTGGTACCCGAGGTGGGCGGTCGCGTAAGCGGCTGGGAGGAAGGGATCAGCTCGTGTCGGGAATTCGTCCTGTTGATGGAAGGGCAAAC 306
1218S/1-1000 161 ACCTAACTTATATACGCAGTGAGATTCAGGTAACACCGATGTGGACAGTGTGTCGTTCGTCGCAACATCGGCTGGTACCCGAGGTGGGCGGTCGCGTAAGCGGCTGGGAGGAAGGGATCAGCTCGTGTCGGGAATTCGTCCTGTTGATGGAAGGGCAAAC 320
1218R_05198/1-351 1 GTGTCGGGAATTCGTCCTGTTGATGGAAGGGCAAAC 36
Mm1218S_05175/1-351 1 GTGTCGGGAATTCGTCCTGTTGATGGAAGGGCAAAC 36
Consensus
ACCTAACTTATATACGCAGTGAGATTCAGGTAACACCGATGTGGACAGTGTGTCGTTCGTCGCAACATCGGCTGGTACCCGAGGTGGGCGGTCGCGTAAGCGGCTGGGAGGAAGGGATCAGCTCGTGTCGGGAATTCGTCCTGTTGATGGAAGGGCAAAC
Occupancy
1218R/1-1000 307 CTAACGTCGGCTCAGAACCTACTGAGCACTGGCGAGGCTGAAGAACGGATCAACTGGGTATCAAAGGCGCTCTGCCGGGCTACCGACCCGGACGAACTCTTTGTTCGTGGGGCCGCACAGCGCAAGGCCGCGGTGATTTGCCGGCACTGCCCGGTGATGC 466
1218S/1-1000 321 CTAACGTCGGCTCAGAACCTACTGAGCACTGGCGAGGCTGAAGAACGGATCAACTGGGTATCAAAGGCGCTCTGCCGGGCTACCGACCCGGACGAACTCTTTGTTCGTGGGGCCGCACAGCGCAAGGCCGCGGTGATTTGCCGGCACTGCCCGGTGATGC 480
1218R_05198/1-351 37 CTAACGTCGGCTCAGAACCTACTGAGCACTGGCGAGGCTGAAGAACGGATCAACTGGGTATCAAAGGCGCTCTGCCGGGCTACCGACCCGGACGAACTCTTTGTTCGTGGGGCCGCACAGCGCAAGGCCGCGGTGATTTGCCGGCACTGCCCGGTGATGC 196
Mm1218S_05175/1-351 37 CTAACGTCGGCTCAGAACCTACTGAGCACTGGCGAGGCTGAAGAACGGATCAACTGGGTATCAAAGGCGCTCTGCCGGGCTACCGACCCGGACGAACTCTTTGTTCGTGGGGCCGCACAGCGCAAGGCCGCGGTGATTTGCCGGCACTGCCCGGTGATGC 196
Consensus
CTAACGTCGGCTCAGAACCTACTGAGCACTGGCGAGGCTGAAGAACGGATCAACTGGGTATCAAAGGCGCTCTGCCGGGCTACCGACCCGGACGAACTCTTTGTTCGTGGGGCCGCACAGCGCAAGGCCGCGGTGATTTGCCGGCACTGCCCGGTGATGC
Occupancy
1218R/1-1000 467 AGGAATGCGGCGCCGACGCGCTCGACAACAAGGTCGAGTTCGGCGTCTGGGGCGGCATGACCGAGCGGCAGCGCCGGGCGCTCCTCAAGCAGCATCCCGAGGTGGTCTCGTGGTCGGACTACTTCGAGAAGCGCAAGCGCCGCAGCGTCGGATAGGGCGC 626
1218S/1-1000 481 AGGAATGCGGCGCCGACGCGCTCGACAACAAGGTCGAGTTCGGCGTCTGGGGCGGCATGACCGAGCGGCAGCGCCGGGCGCTCCTCAAGCAGCATCCCGAGGTGGTCTCGTGGTCGGACTACTTCGAGAAGCGCAAGCGCCGCAGCGTCGGATAGGGCGC 640
1218R_05198/1-351 197 AGGAATGCGGCGCCGACGCGCTCGACAACAAGGTCGAGTTCGGCGTCTGGGGCGGCATGACCGAGCGGCAGCGCCGGGCGCTCCTCAAGCAGCATCCCGAGGTGGTCTCGTGGTCGGACTACTTCGAGAAGCGCAAGCGCCGCAGCGTCGGATAG----- 351
Mm1218S_05175/1-351 197 AGGAATGCGGCGCCGACGCGCTCGACAACAAGGTCGAGTTCGGCGTCTGGGGCGGCATGACCGAGCGGCAGCGCCGGGCGCTCCTCAAGCAGCATCCCGAGGTGGTCTCGTGGTCGGACTACTTCGAGAAGCGCAAGCGCCGCAGCGTCGGATAG----- 351
Consensus
AGGAATGCGGCGCCGACGCGCTCGACAACAAGGTCGAGTTCGGCGTCTGGGGCGGCATGACCGAGCGGCAGCGCCGGGCGCTCCTCAAGCAGCATCCCGAGGTGGTCTCGTGGTCGGACTACTTCGAGAAGCGCAAGCGCCGCAGCGTCGGATAGGGCGC
Occupancy
1218R/1-1000 627 ACGTGACCGGGGGGTCAGCGCAACGGCGGGCAGTCCCTTCCTGATTACTTTTTTGTTACACGCGCCGGTTGGATGCACTCTAATTGTCTTGTGCCCAATAGGAACTGGCGTGTGCCCAATTGGACCTACCGTCGCGGTCGGTCCTGACGGGCTGGTGTAG 786
1218S/1-1000 641 ACGTGACCGGGGGGTCAGCGCAACGGCGGGCAGTCCCTTCCTGATTACTTTTTTGTTACACGCGCCGGTTGGATGCACTCTAATTGTCTTGTGCCCAATAGGAACTGGCGTGTGCCCAATTGGACCTACCGTCGCGGTCGGTCCTGACGGGCTGGTGTAG 800
1218R_05198/1-351
Mm1218S_05175/1-351
Consensus
ACGTGACCGGGGGGTCAGCGCAACGGCGGGCAGTCCCTTCCTGATTACTTTTTTGTTACACGCGCCGGTTGGATGCACTCTAATTGTCTTGTGCCCAATAGGAACTGGCGTGTGCCCAATTGGACCTACCGTCGCGGTCGGTCCTGACGGGCTGGTGTAG
Occupancy
1218R/1-1000 787 CGCCCCCGCCATTGCCGGCGGCGGTGATCTGATCGGCGAGTGCGCGCAGGGCGTCCAAGTCGGAAACGTCGAATGGCAGCGACGGCACGCCGATGATCGGCACATGCGGATTGGCCCCGGTGAATCGGGACAGCAACCGAATCTCGCGCTTGGCTGTCTG 946
1218S/1-1000 801 CGCCCCCGCCATTGCCGGCGGCGGTGATCTGATCGGCGAGTGCGCGCAGGGCGTCCAAGTCGGAAACGTCGAATGGCAGCGACGGCACGCCGATGATCGGCACATGCGGATTGGCCCCGGTGAATCGGGACAGCAACCGAATCTCGCGCTTGGCTGTCTG 960
1218R_05198/1-351
Mm1218S_05175/1-351
Consensus
CGCCCCCGCCATTGCCGGCGGCGGTGATCTGATCGGCGAGTGCGCGCAGGGCGTCCAAGTCGGAAACGTCGAATGGCAGCGACGGCACGCCGATGATCGGCACATGCGGATTGGCCCCGGTGAATCGGGACAGCAACCGAATCTCGCGCTTGGCTGTCTG
Occupancy
1218R/1-1000 947 TGCCCGGTCGGCGTGGATGCGCAGCACCGCCGCCGTCAGCGCGGCCGTCTCCGA 1000
1218S/1-1000 961 TGCCCGGTCGGCGTGGATGCGCAGCACCGCCGCCGTCAGC-------------- 1000
1218R_05198/1-351
Mm1218S_05175/1-351
Consensus
TGCCCGGTCGGCGTGGATGCGCAGCACCGCCGCCGTCAGCGCGGCCGTCTCCGA
Occupancy

Fig S5C

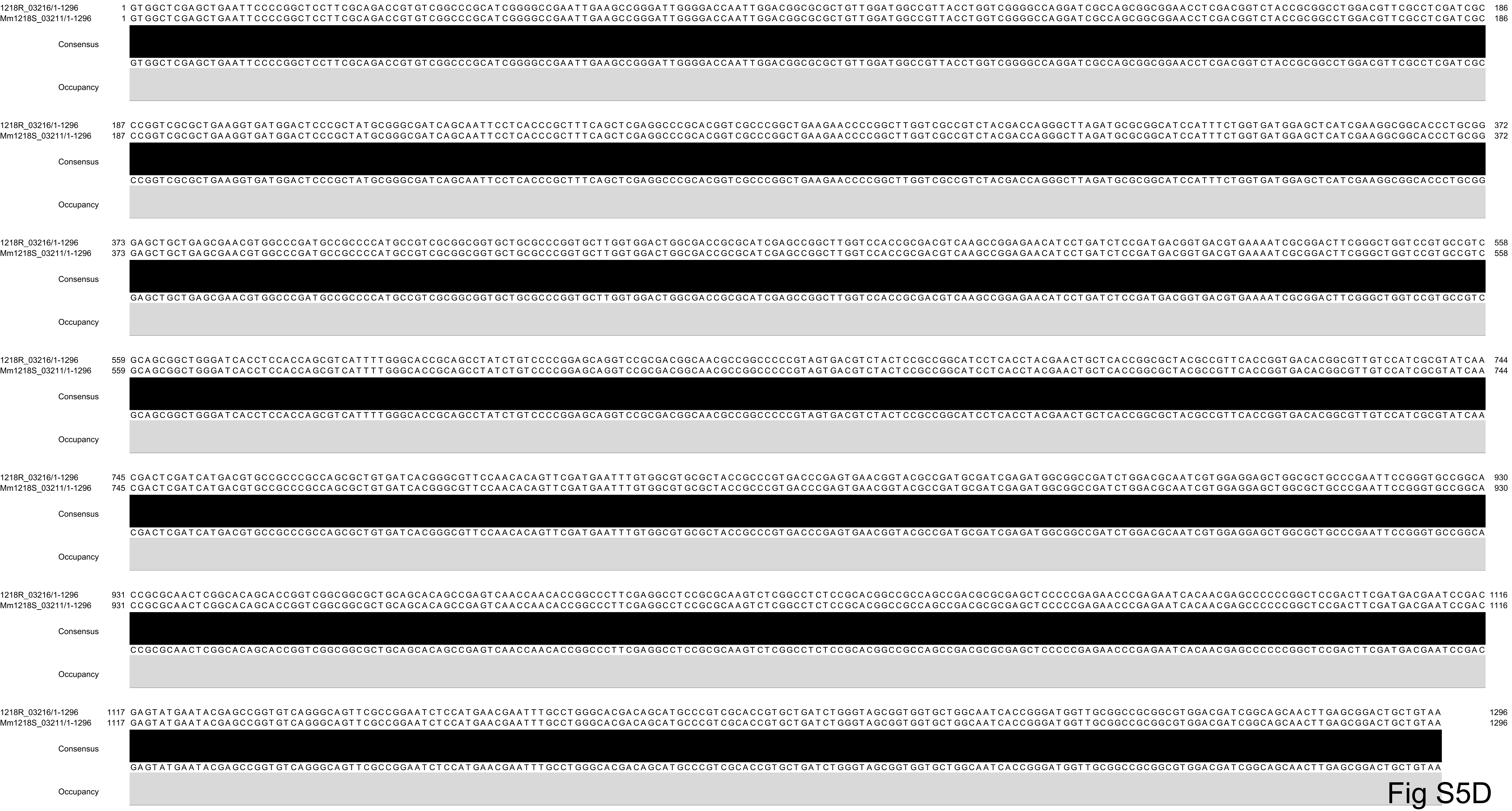
1218R_03216/1-1296
Mm1218S_03211/1-1296
Consensus
Occupancy
1
186
187
372
373
558
559
744
745
930
931
1116
1117
1296

Fig S5D

Figure S6 Bar plots showing transcription of virulence genes in 1218R, 1218S, *Mmar*^{CCUG} and *Mmar*^{M} .

(a) Transcript levels (distribution, expressed in TPM values) in exponentially growing and stationary 1218R cells.

(b) Transcript levels (distribution, expressed in TPM values) in exponentially growing and stationary 1218S cells.

(c) Comparing transcript levels in exponentially growing (red colour) and stationary (turquoise) 1218R vs 1218S cells. Negative $\log_2$-values suggest that the corresponding mRNA is more abundant in 1218S cells while a positive value suggests higher levels in 1218R cells.

(d) Transcript levels (distribution, expressed in TPM values) in exponentially growing and stationary *Mmar*^{CCUG} cells.

(e) Comparing transcript levels in exponentially growing (red colour) and stationary (turquoise) 1218R vs *Mmar*^{CCUG} cells. Negative $\log_2$-values suggest that the corresponding mRNA is more abundant in *Mmar*^{CCUG} cells while a positive value suggests higher levels in 1218R cells.

(f) Transcript levels (distribution, expressed in TPM values) in exponentially growing and stationary *Mmar*^{M} cells.

(g) Comparing transcript levels in exponentially growing (red colour) and stationary (turquoise) 1218R vs *Mmar*^{M} cells. Negative $\log_2$-values suggest that the corresponding mRNA is more abundant in *Mmar*^{M} cells while a positive value suggests higher levels in 1218R cells.

Statistical significance, see Materials and Methods; *p-value < 0.05; **p-value < 0.01; ***p-value < 0.001.

Fig S6A

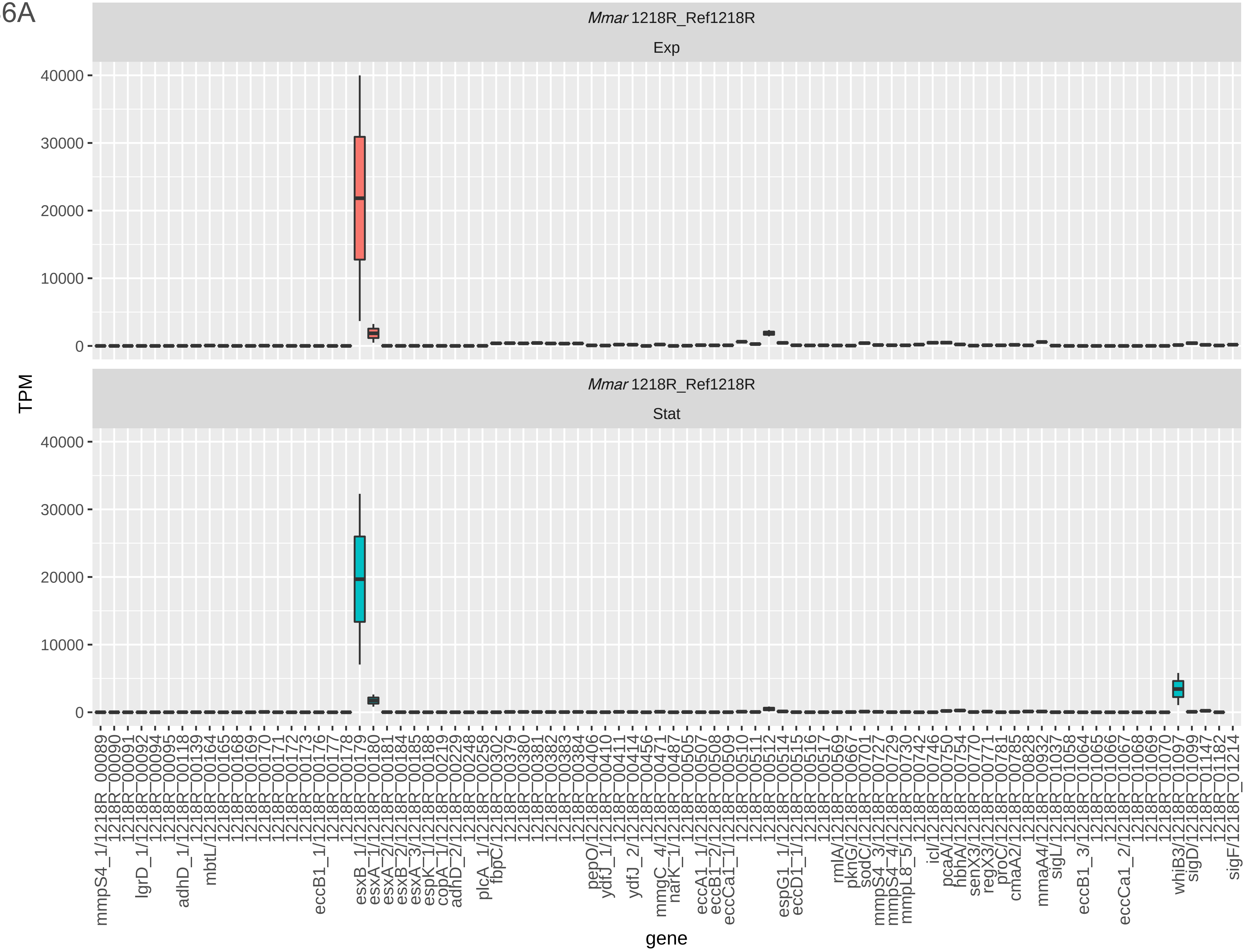

Mmar 1218R_Ref1218R
Exp
Mmar 1218R_Ref1218R
Stat
TPM
40000
30000
20000
10000
0
gene
mmpS4_1/1218R_00089
1218R_00090
1218R_00091
lgrD_1/1218R_00092
1218R_00094
1218R_00095
adhD_1/1218R_00118
1218R_00139
mbtL/1218R_00164
1218R_00165
1218R_00168
1218R_00169
1218R_00170
1218R_00171
1218R_00172
1218R_00173
1218R_00176
eccB1_1/1218R_00177
1218R_00178
esxB_1/1218R_00179
esxA_1/1218R_00180
esxA_2/1218R_00181
esxB_2/1218R_00184
esxA_3/1218R_00185
espK_1/1218R_00188
copA_1/1218R_00219
adhD_2/1218R_00229
1218R_00248
plcA_1/1218R_00258
fbpC/1218R_00302
1218R_00379
1218R_00380
1218R_00381
1218R_00382
1218R_00383
1218R_00384
pepO/1218R_00406
ydfJ_1/1218R_00410
1218R_00411
ydfJ_2/1218R_00414
1218R_00456
mmgC_4/1218R_00471
narK_1/1218R_00487
1218R_00505
eccA1_1/1218R_00507
eccB1_2/1218R_00508
eccCa1_1/1218R_00509
1218R_00510
1218R_00511
1218R_00512
espG1_1/1218R_00514
eccD1_1/1218R_00515
1218R_00516
1218R_00517
rmlA/1218R_00569
pknG/1218R_00667
sodC/1218R_00701
mmpS4_3/1218R_00727
mmpS4_4/1218R_00729
mmpL8_5/1218R_00730
1218R_00742
icl/1218R_00746
pcaA/1218R_00750
hbhA/1218R_00754
senX3/1218R_00770
regX3/1218R_00771
proC/1218R_00781
cmaA2/1218R_00785
1218R_00828
mmaA4/1218R_00932
sigL/1218R_01037
1218R_01058
eccB1_3/1218R_01064
1218R_01065
1218R_01066
eccCa1_2/1218R_01067
1218R_01068
1218R_01069
1218R_01070
whiB3/1218R_01097
sigD/1218R_01099
1218R_01147
1218R_01182
sigF/1218R_01214

Fig S6A

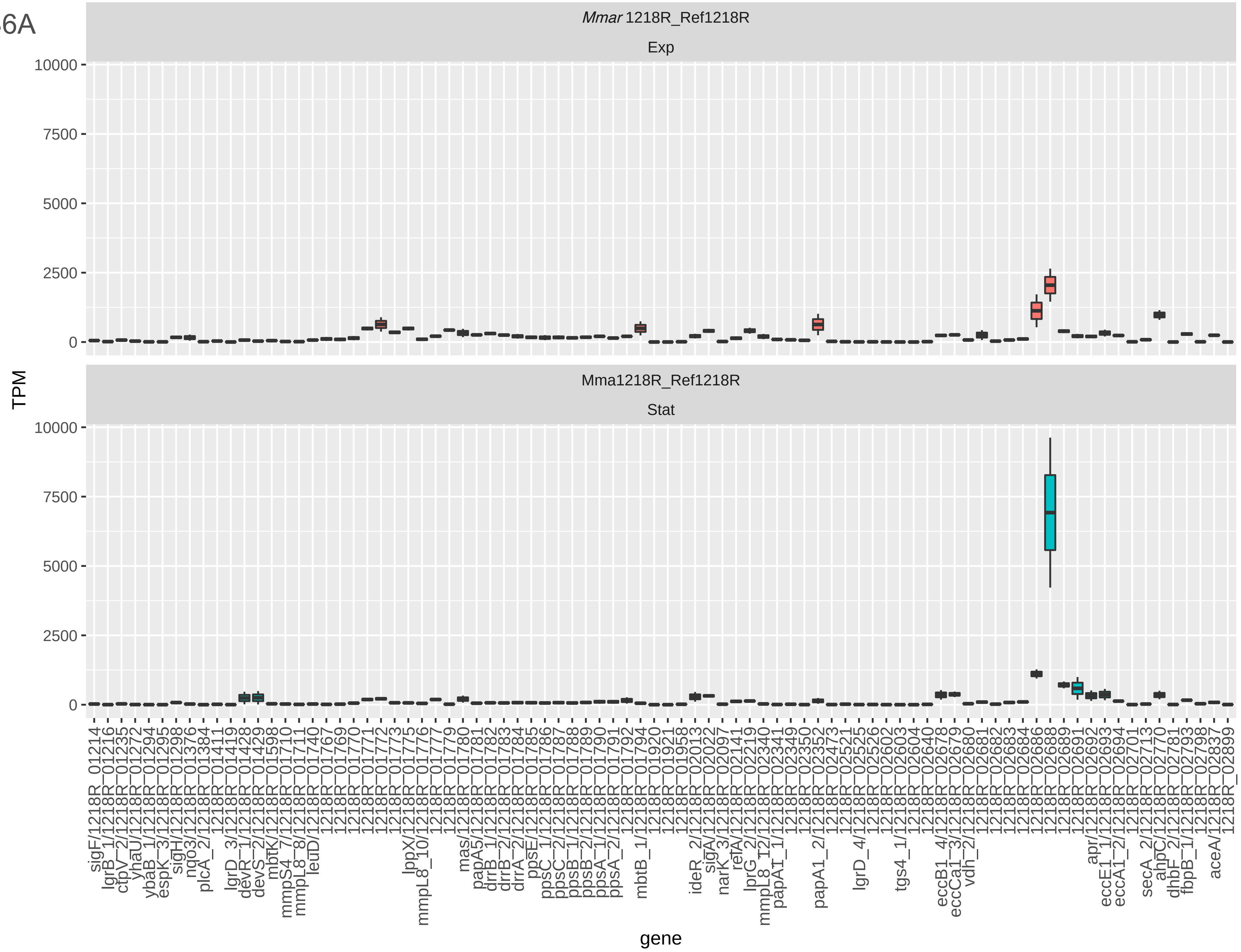
Mmar 1218R_Ref1218R
Exp
Mma1218R_Ref1218R
Stat
TPM
10000
7500
5000
2500
0
gene
sigF/1218R_01214
lgrB_1/1218R_01216
ctpV_2/1218R_01235
yhaU/1218R_01272
ybaB_1/1218R_01294
espK_3/1218R_01295
sigH/1218R_01298
ngo3/1218R_01376
plcA_2/1218R_01384
1218R_01411
lgrD_3/1218R_01419
devR_1/1218R_01428
devS_2/1218R_01429
mbtK/1218R_01598
mmpS4_7/1218R_01710
mmpL8_8/1218R_01711
leuD/1218R_01740
1218R_01767
1218R_01769
1218R_01770
1218R_01771
1218R_01772
1218R_01773
lppX/1218R_01775
mmpL8_10/1218R_01776
1218R_01777
1218R_01779
mas/1218R_01780
papA5/1218R_01781
drrB_1/1218R_01782
drrB_2/1218R_01783
drrA_2/1218R_01784
ppsE/1218R_01785
ppsC_1/1218R_01786
ppsC_2/1218R_01787
ppsB_1/1218R_01788
ppsB_2/1218R_01789
ppsA_1/1218R_01790
ppsA_2/1218R_01791
1218R_01792
mbtB_1/1218R_01794
1218R_01920
1218R_01921
1218R_01958
ideR_2/1218R_02013
sigA/1218R_02022
narK_3/1218R_02097
relA/1218R_02141
lprG_2/1218R_02219
mmpL8_12/1218R_02340
papA1_1/1218R_02341
1218R_02349
1218R_02350
papA1_2/1218R_02352
1218R_02473
1218R_02521
lgrD_4/1218R_02525
1218R_02526
1218R_02602
tgs4_1/1218R_02603
1218R_02604
1218R_02640
eccB1_4/1218R_02678
eccCa1_3/1218R_02679
vdh_2/1218R_02680
1218R_02681
1218R_02682
1218R_02683
1218R_02684
1218R_02686
1218R_02688
1218R_02689
apr/1218R_02691
eccE1_1/1218R_02692
eccA1_2/1218R_02693
1218R_02694
1218R_02701
secA_2/1218R_02713
ahpC/1218R_02770
dhbF_2/1218R_02781
fbpB_1/1218R_02798
aceA/1218R_02837
1218R_02899

Fig S6A

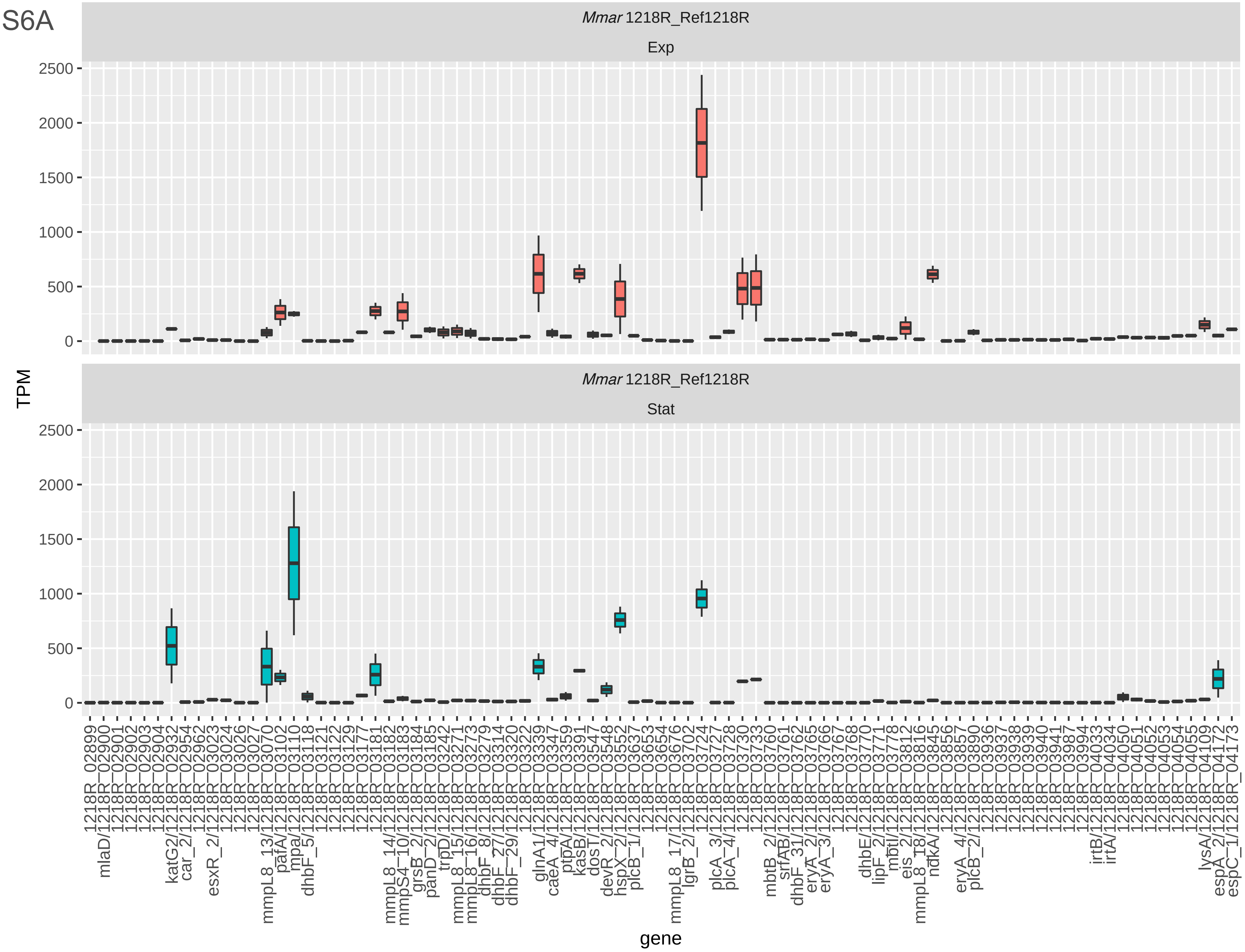
Mmar 1218R_Ref1218R
Exp
Mmar 1218R_Ref1218R
Stat
2500
2000
1500
1000
500
0
TPM
gene

Fig S6A

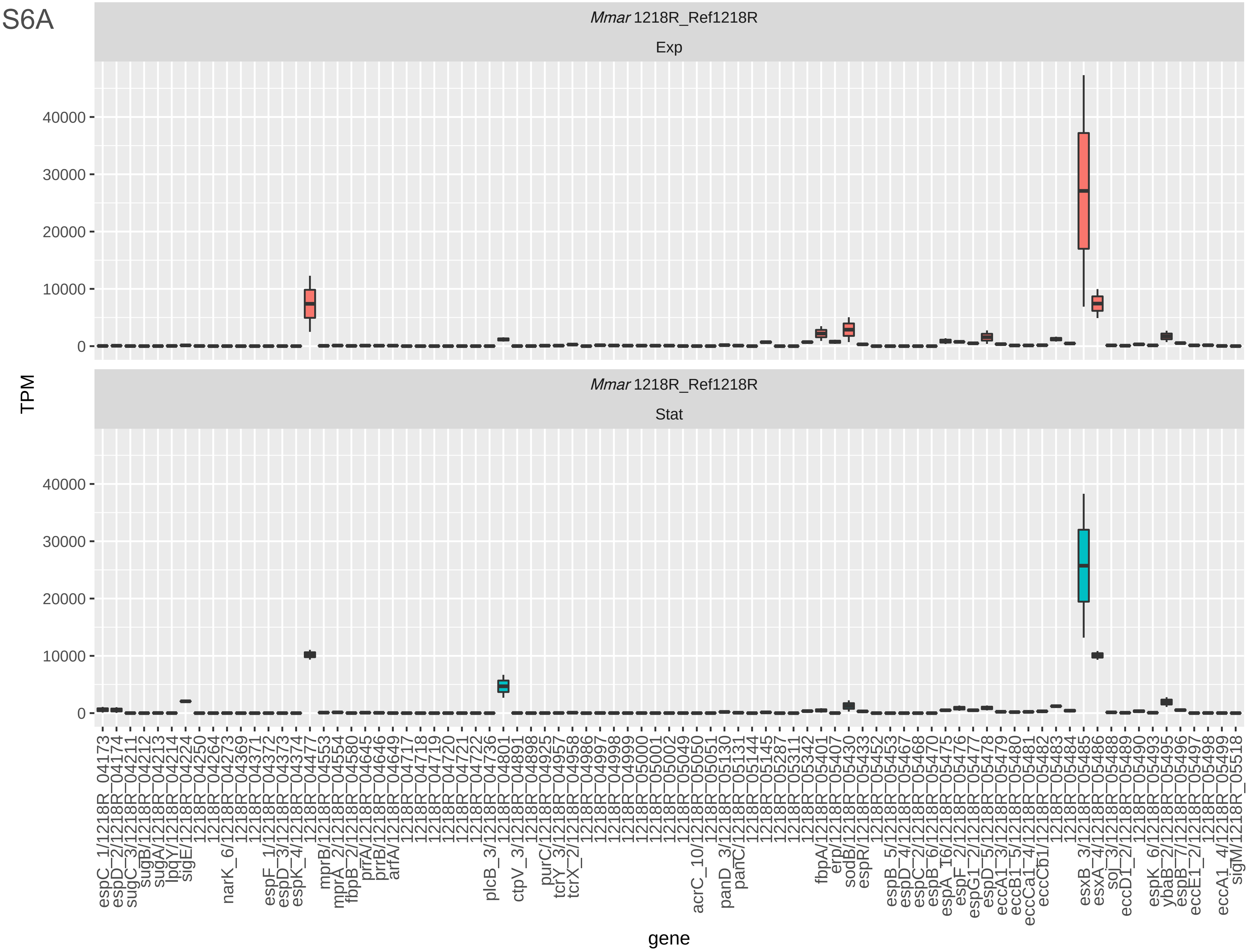
Mmar 1218R_Ref1218R
Exp
Mmar 1218R_Ref1218R
Stat
TPM
40000
30000
20000
10000
0
gene

Fig S6B

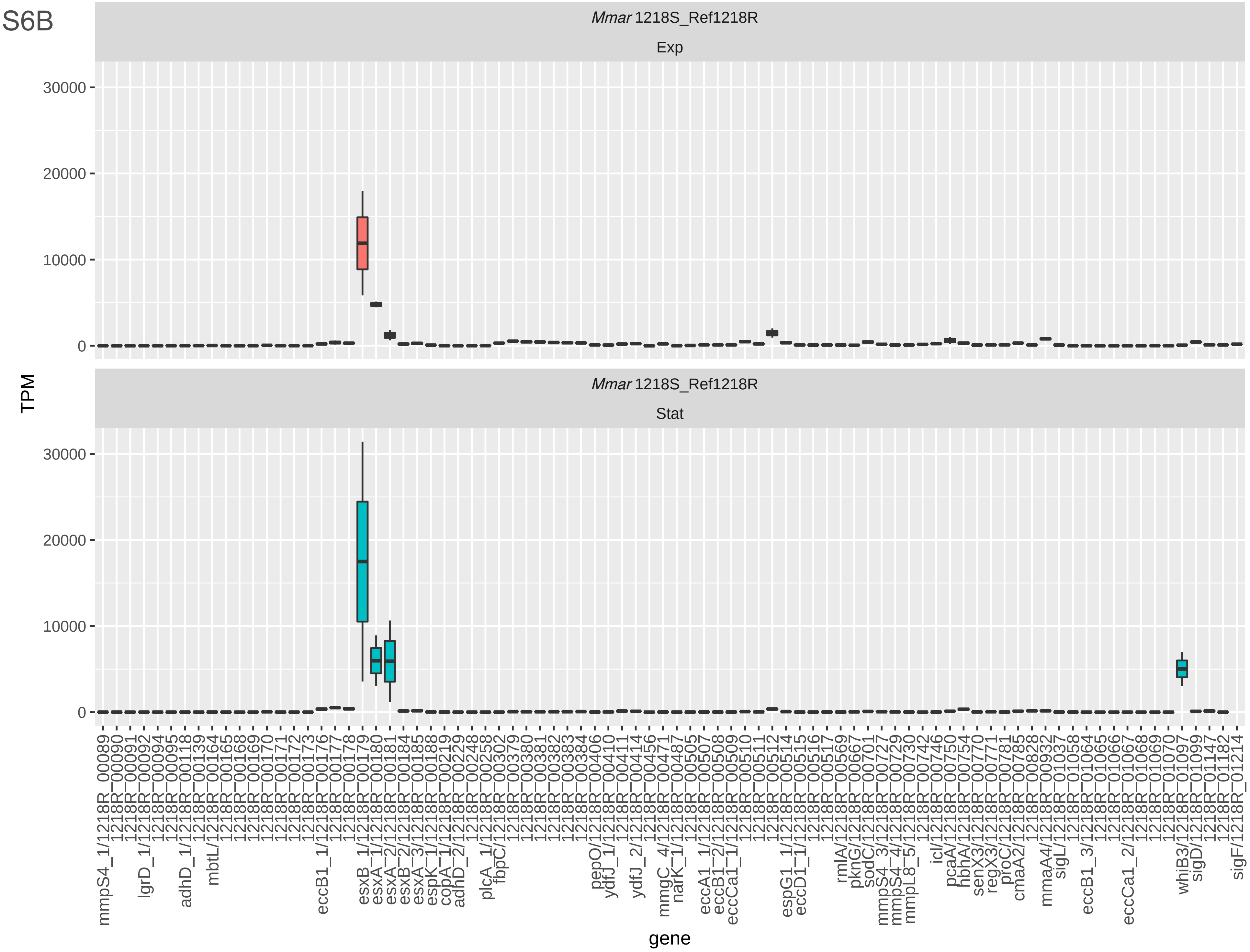
Mmar 1218S_Ref1218R
Exp
Mmar 1218S_Ref1218R
Stat
TPM
30000
20000
10000
0
gene

Fig S6B

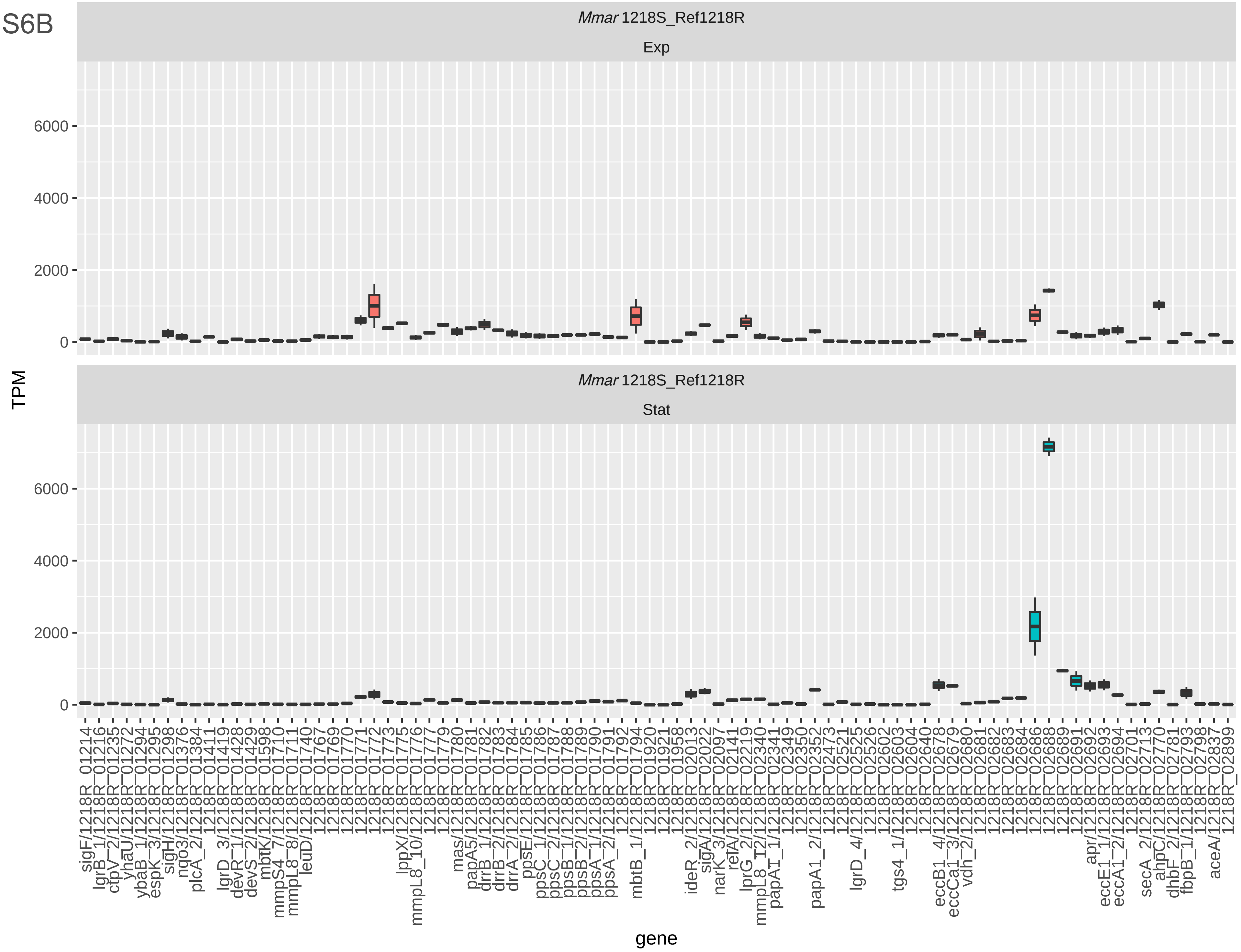

Mmar 1218S_Ref1218R
Exp
Mmar 1218S_Ref1218R
Stat
TPM
0
2000
4000
6000
gene

Fig S6B

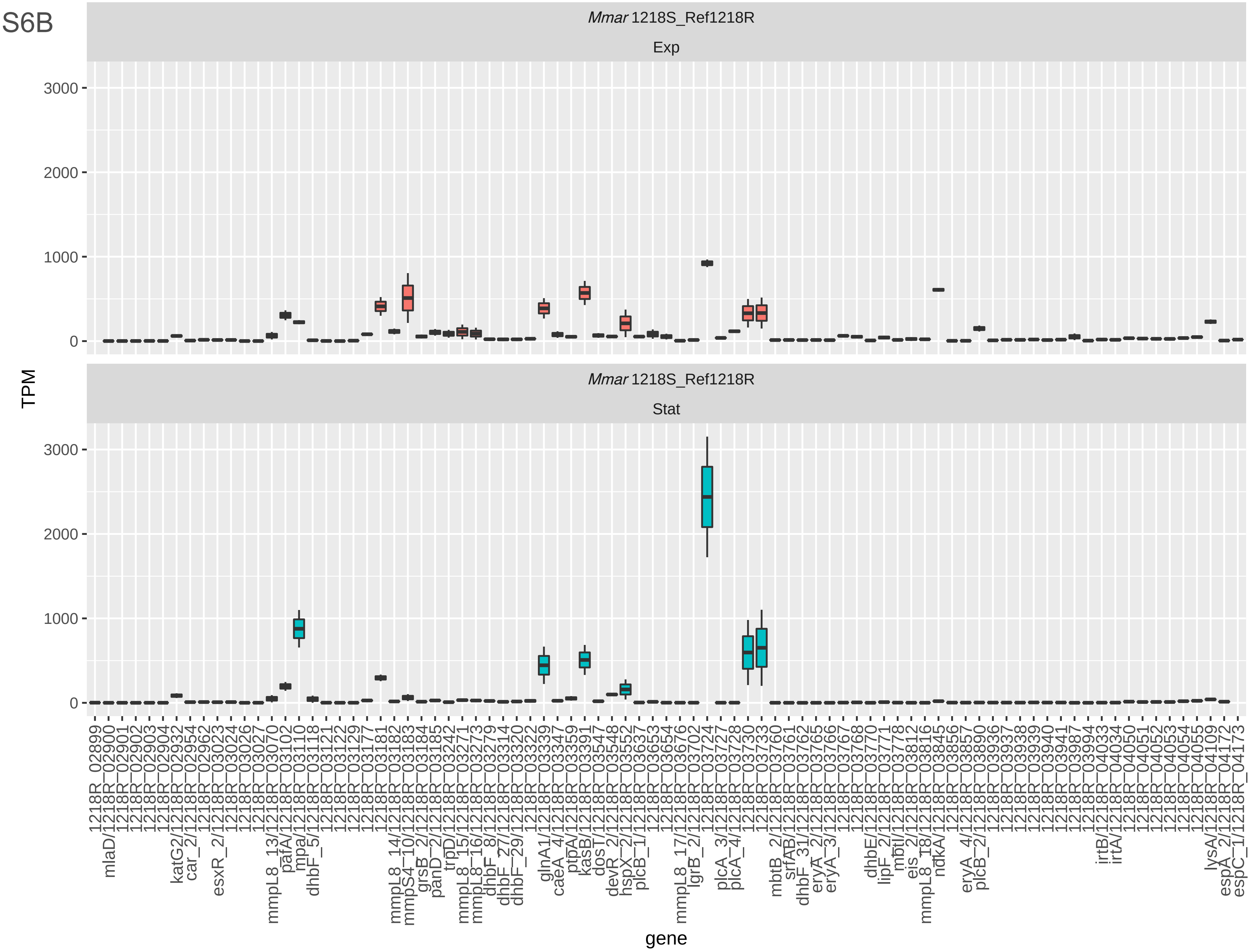
Mmar 1218S_Ref1218R
Exp
Mmar 1218S_Ref1218R
Stat
TPM
gene
3000
2000
1000
0

Fig S6B

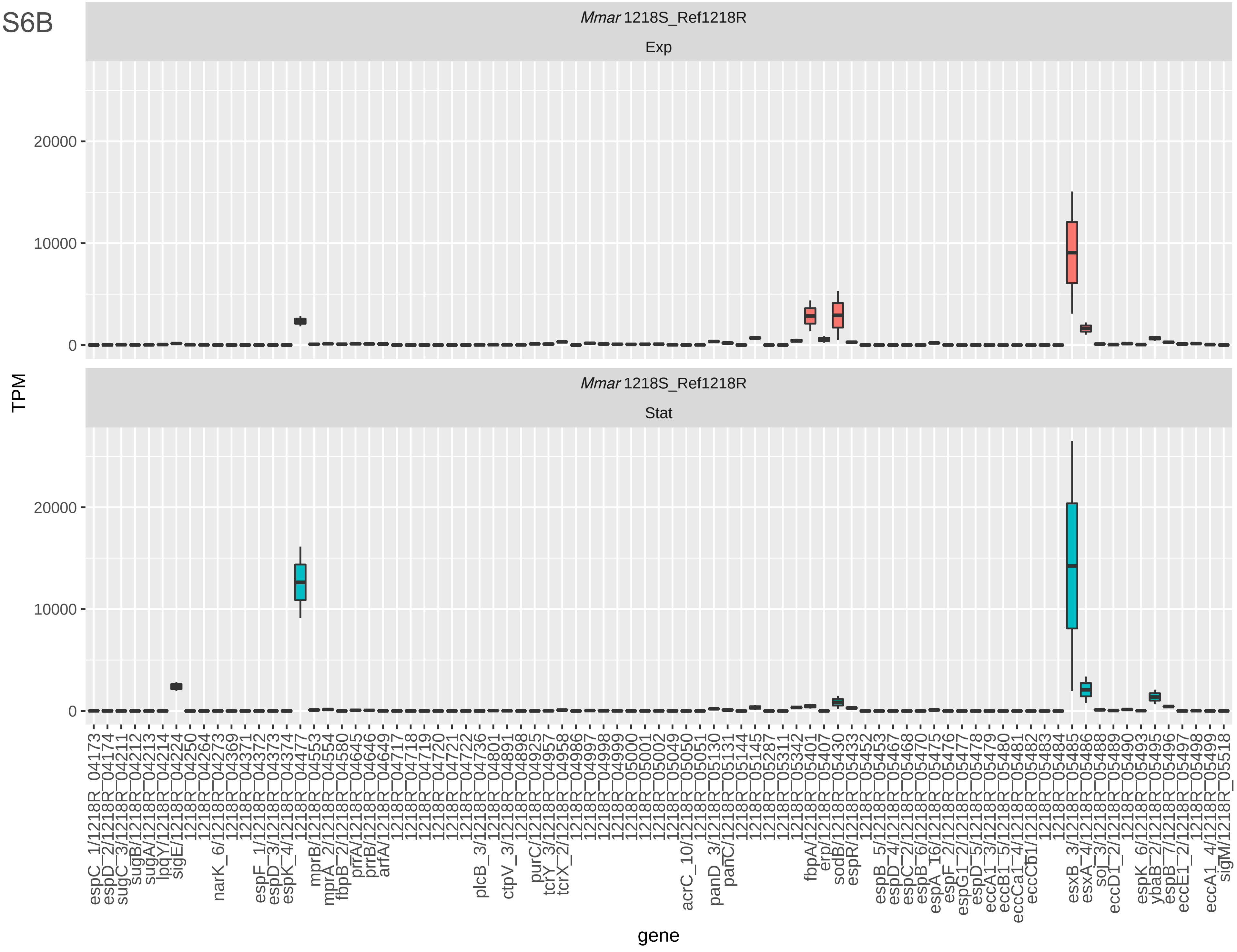

Mmar 1218S_Ref1218R
Exp
Mmar 1218S_Ref1218R
Stat
TPM
0
10000
20000
gene
espC_1/1218R_04173
espD_2/1218R_04174
sugC_3/1218R_04211
sugB/1218R_04212
sugA/1218R_04213
lpqY/1218R_04214
sigE/1218R_04224
1218R_04250
1218R_04264
narK_6/1218R_04273
1218R_04369
1218R_04371
espF_1/1218R_04372
espD_3/1218R_04373
espK_4/1218R_04374
1218R_04477
mprB/1218R_04553
mprA_2/1218R_04554
fbpB_2/1218R_04580
prrA/1218R_04645
prrB/1218R_04646
arfA/1218R_04649
1218R_04717
1218R_04718
1218R_04719
1218R_04720
1218R_04721
1218R_04722
plcB_3/1218R_04736
1218R_04801
ctpV_3/1218R_04891
1218R_04898
purC/1218R_04925
tcrY_3/1218R_04957
tcrX_2/1218R_04958
1218R_04986
1218R_04997
1218R_04998
1218R_04999
1218R_05000
1218R_05001
1218R_05002
1218R_05049
acrC_10/1218R_05050
1218R_05051
panD_3/1218R_05130
panC/1218R_05131
1218R_05144
1218R_05145
1218R_05287
1218R_05311
1218R_05342
fbpA/1218R_05401
erp/1218R_05407
sodB/1218R_05430
espR/1218R_05433
1218R_05452
espB_5/1218R_05453
espD_4/1218R_05467
espC_2/1218R_05468
espB_6/1218R_05470
espA_1/1218R_05475
espF_2/1218R_05476
espG1_2/1218R_05477
espD_5/1218R_05478
eccA1_3/1218R_05479
eccB1_5/1218R_05480
eccCa1_4/1218R_05481
eccCb1/1218R_05482
1218R_05483
1218R_05484
esxB_3/1218R_05485
esxA_4/1218R_05486
soj_3/1218R_05488
eccD1_2/1218R_05489
1218R_05490
espK_6/1218R_05493
ybaB_2/1218R_05495
espB_7/1218R_05496
eccE1_2/1218R_05497
1218R_05498
eccA1_4/1218R_05499
sigM/1218R_05518

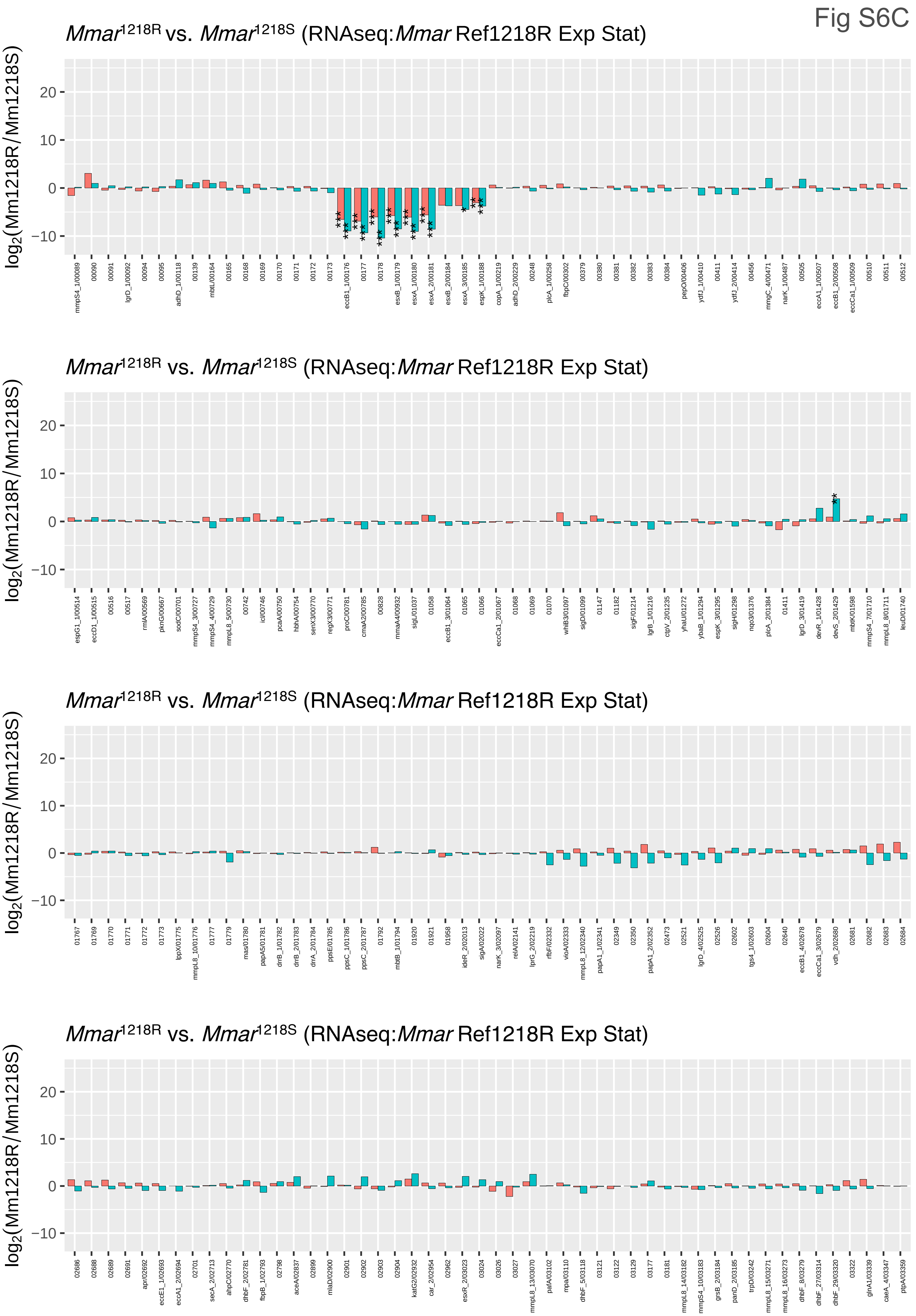
Fig S6C
Mmar1218R vs. Mmar1218S (RNAseq:Mmar Ref1218R Exp Stat)
log2(Mm1218R/Mm1218S)
Mmar1218R vs. Mmar1218S (RNAseq:Mmar Ref1218R Exp Stat)
log2(Mm1218R/Mm1218S)
Mmar1218R vs. Mmar1218S (RNAseq:Mmar Ref1218R Exp Stat)
log2(Mm1218R/Mm1218S)
Mmar1218R vs. Mmar1218S (RNAseq:Mmar Ref1218R Exp Stat)
log2(Mm1218R/Mm1218S)

Fig 6C

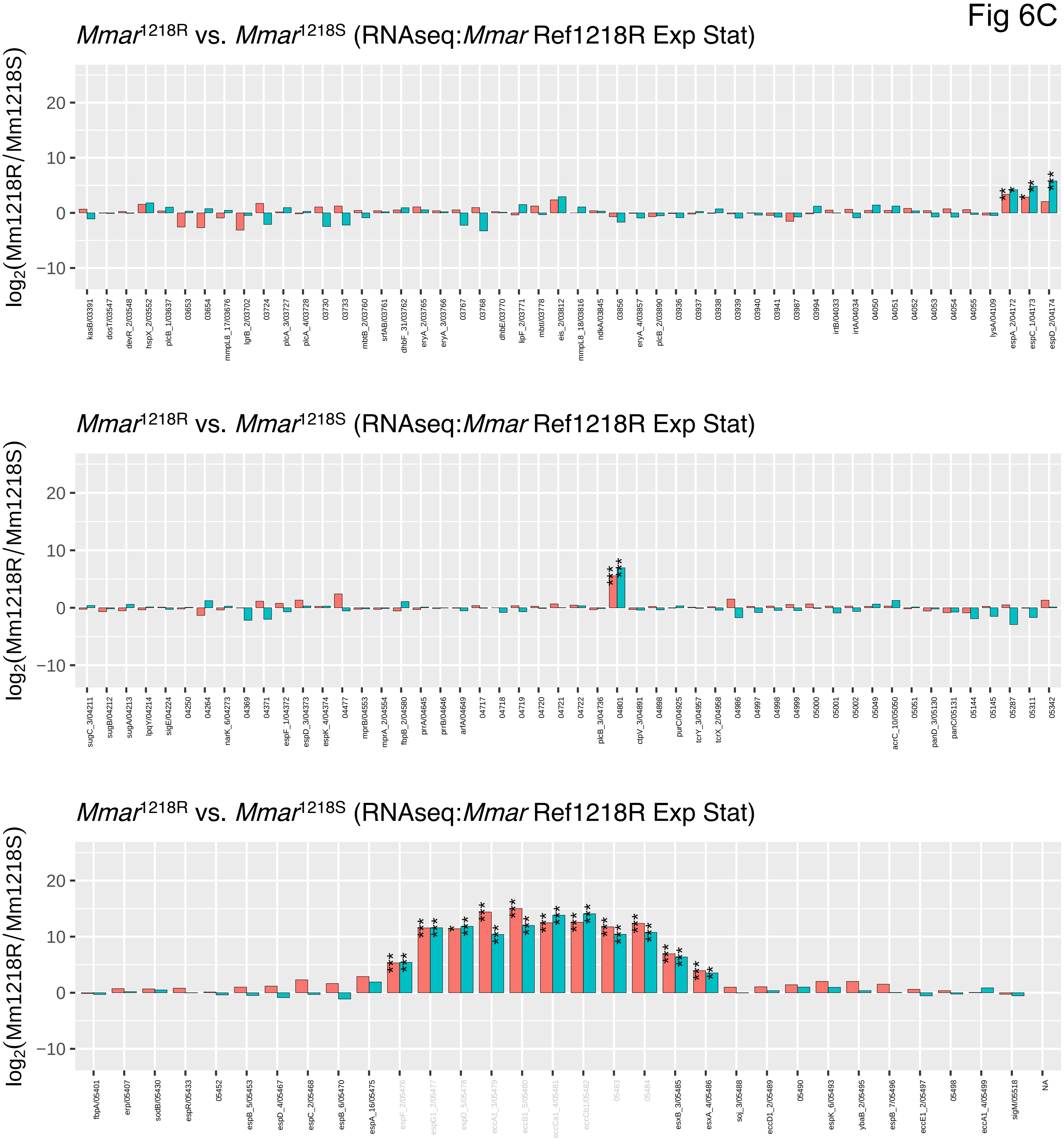

Mmar1218R vs. Mmar1218S (RNAseq:Mmar Ref1218R Exp Stat)
log2(Mm1218R/Mm1218S)
20
10
0
−10
kasB/03391
dosT/03547
devR_2/03548
hspX_2/03552
plcB_1/03637
03653
03654
mmpL8_17/03676
lgrB_2/03702
03724
plcA_3/03727
plcA_4/03728
03730
03733
mbtB_2/03760
srfAB/03761
dhbF_31/03762
eryA_2/03765
eryA_3/03766
03767
03768
dhbE/03770
lipF_2/03771
mbtI/03778
eis_2/03812
mmpL8_18/03816
ndkA/03845
03856
eryA_4/03857
plcB_2/03890
03936
03937
03938
03939
03940
03941
03987
03994
irtB/04033
irtA/04034
04050
04051
04052
04053
04054
04055
lysA/04109
espA_2/04172
espC_1/04173
espD_2/04174
Mmar1218R vs. Mmar1218S (RNAseq:Mmar Ref1218R Exp Stat)
log2(Mm1218R/Mm1218S)
20
10
0
−10
sugC_3/04211
sugB/04212
sugA/04213
lpqY/04214
sigE/04224
04250
04264
narK_6/04273
04369
04371
espF_1/04372
espD_3/04373
espK_4/04374
04477
mprB/04553
mprA_2/04554
fbpB_2/04580
prrA/04645
prrB/04646
arfA/04649
04717
04718
04719
04720
04721
04722
plcB_3/04736
04801
ctpV_3/04891
04898
purC/04925
tcrY_3/04957
tcrX_2/04958
04986
04997
04998
04999
05000
05001
05002
05049
acrC_10/05050
05051
panD_3/05130
panC/05131
05144
05145
05287
05311
05342
Mmar1218R vs. Mmar1218S (RNAseq:Mmar Ref1218R Exp Stat)
log2(Mm1218R/Mm1218S)
20
10
0
−10
fbpA/05401
erp/05407
sodB/05430
espR/05433
05452
espB_5/05453
espD_4/05467
espC_2/05468
espB_6/05470
espA_16/05475
espF_2/05476
espG1_2/05477
espD_5/05478
eccA1_3/05479
eccB1_5/05480
eccCa1_4/05481
eccCb1/05482
05483
05484
esxB_3/05485
esxA_4/05486
soj_3/05488
eccD1_2/05489
05490
espK_6/05493
ybaB_2/05495
espB_7/05496
eccE1_2/05497
05498
eccA1_4/05499
sigM/05518
NA

Fig S6D

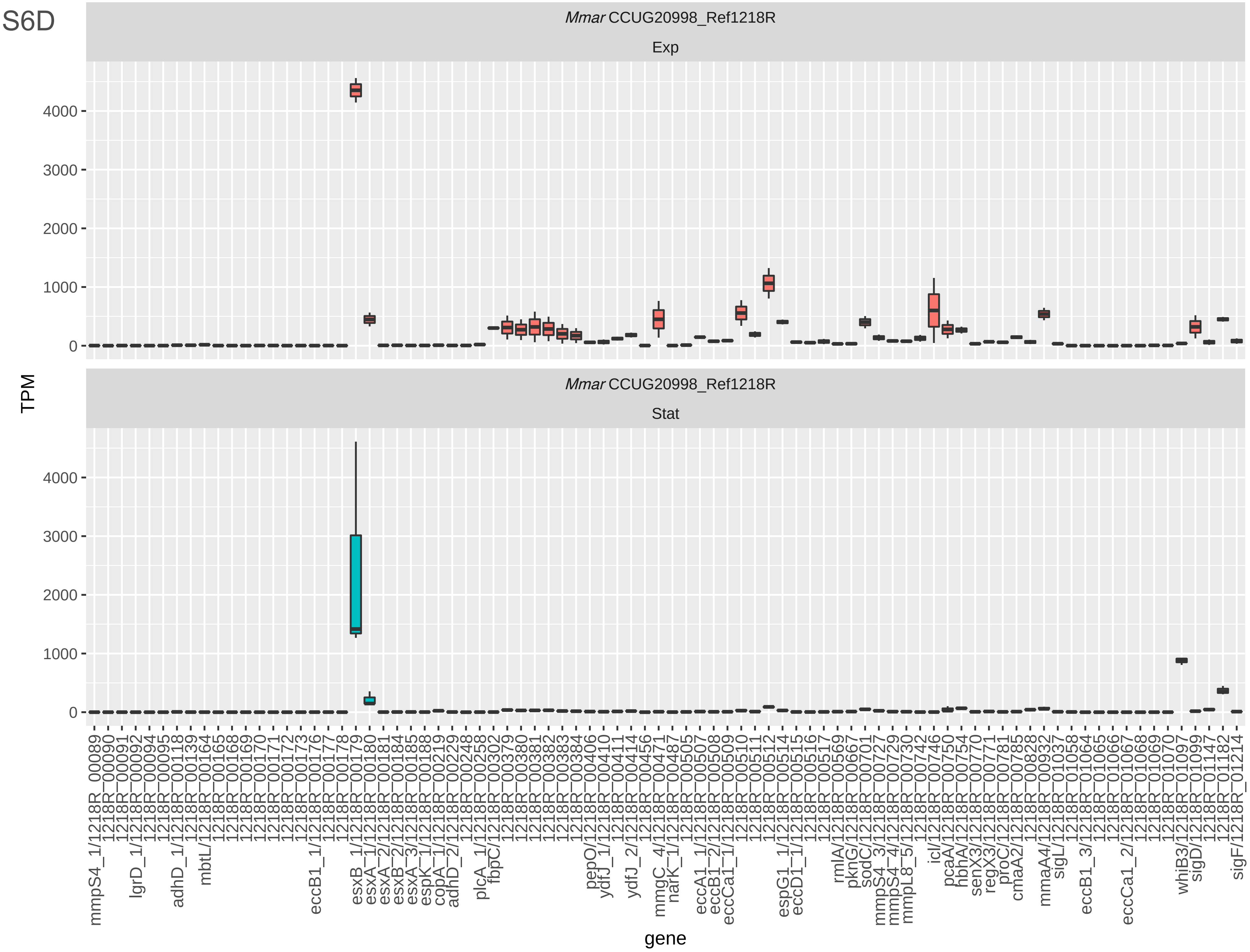
Mmar CCUG20998_Ref1218R
Exp
Mmar CCUG20998_Ref1218R
Stat
TPM
4000
3000
2000
1000
0
gene

Fig S6D

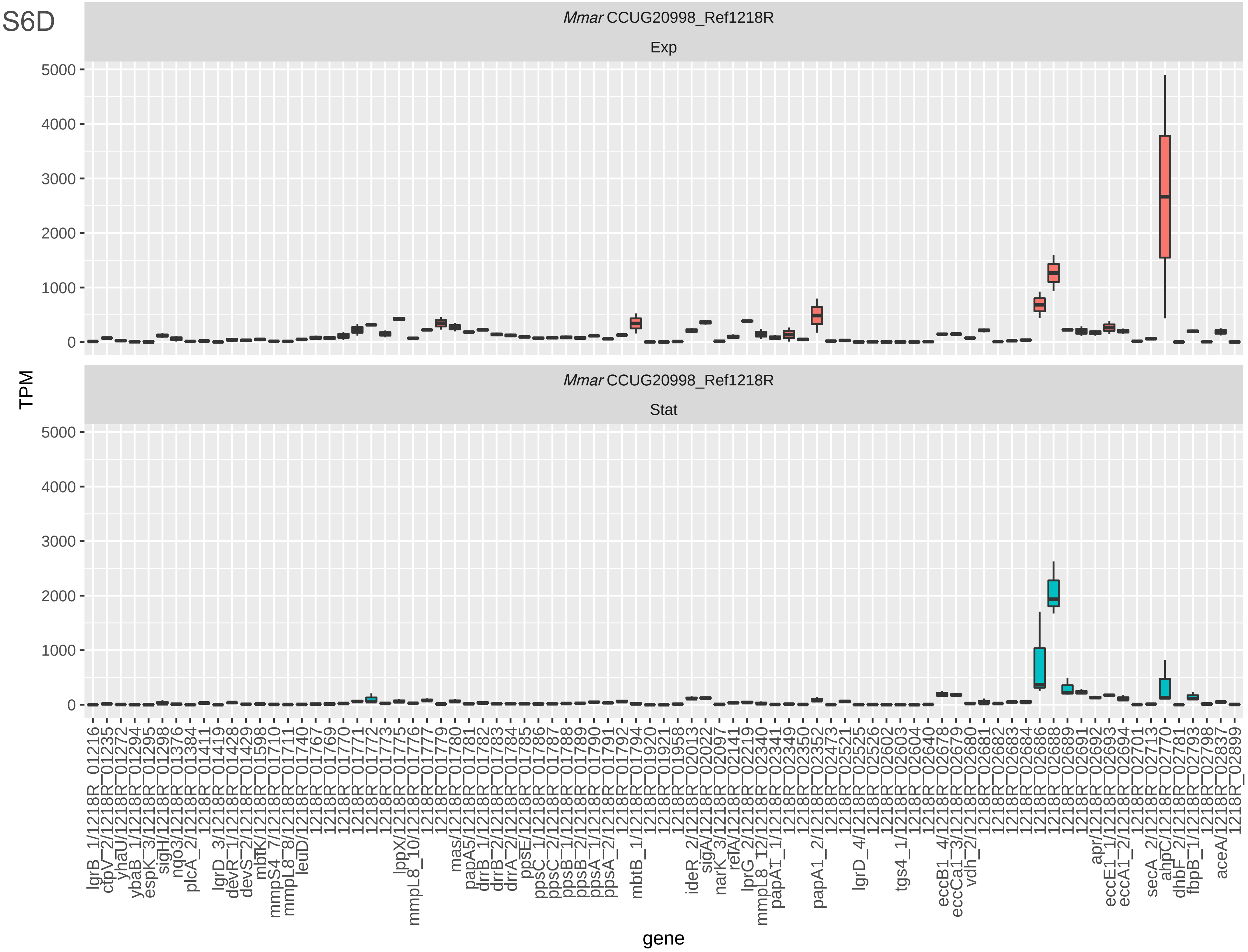
Mmar CCUG20998_Ref1218R
Exp
Stat
TPM
gene
0
1000
2000
3000
4000
5000

Fig S6D

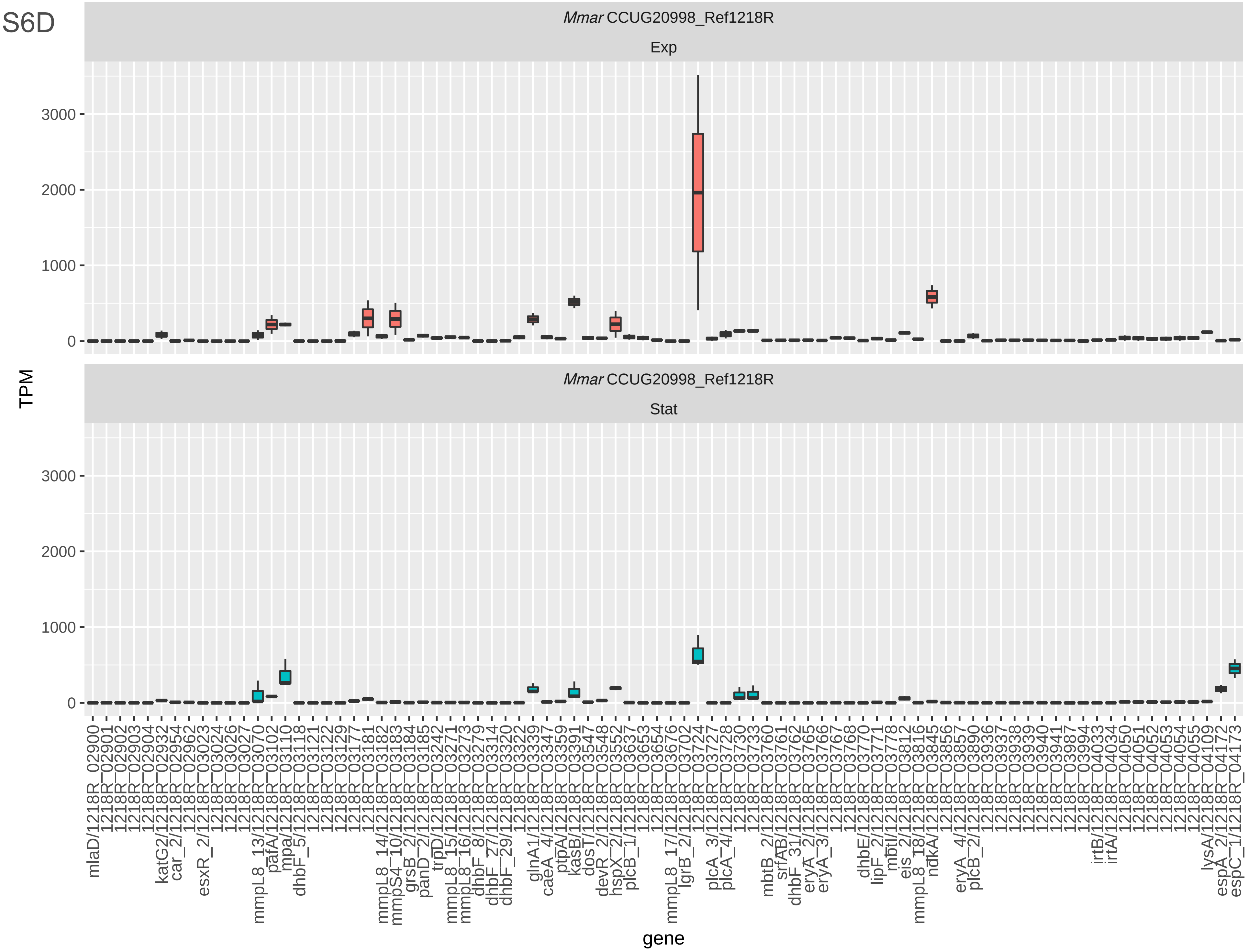
Mmar CCUG20998_Ref1218R
Exp
Mmar CCUG20998_Ref1218R
Stat
TPM
3000
2000
1000
0
gene
mlaD/1218R_02900
1218R_02901
1218R_02902
1218R_02903
1218R_02904
katG2/1218R_02932
car_2/1218R_02954
1218R_02962
esxR_2/1218R_03023
1218R_03024
1218R_03026
1218R_03027
mmpL8_13/1218R_03070
pafA/1218R_03102
mpa/1218R_03110
dhbF_5/1218R_03118
1218R_03121
1218R_03122
1218R_03129
1218R_03177
1218R_03181
mmpL8_14/1218R_03182
mmpS4_10/1218R_03183
grsB_2/1218R_03184
panD_2/1218R_03185
trpD/1218R_03242
mmpL8_15/1218R_03271
mmpL8_16/1218R_03273
dhbF_8/1218R_03279
dhbF_27/1218R_03314
dhbF_29/1218R_03320
1218R_03322
glnA1/1218R_03339
caeA_4/1218R_03347
ptpA/1218R_03359
kasB/1218R_03391
dosT/1218R_03547
devR_2/1218R_03548
hspX_2/1218R_03552
plcB_1/1218R_03637
1218R_03653
1218R_03654
mmpL8_17/1218R_03676
lgrB_2/1218R_03702
1218R_03724
plcA_3/1218R_03727
plcA_4/1218R_03728
1218R_03730
1218R_03733
mbtB_2/1218R_03760
srfAB/1218R_03761
dhbF_31/1218R_03762
eryA_2/1218R_03765
eryA_3/1218R_03766
1218R_03767
1218R_03768
dhbE/1218R_03770
lipF_2/1218R_03771
mbtI/1218R_03778
eis_2/1218R_03812
mmpL8_18/1218R_03816
ndkA/1218R_03845
1218R_03856
eryA_4/1218R_03857
plcB_2/1218R_03890
1218R_03936
1218R_03937
1218R_03938
1218R_03939
1218R_03940
1218R_03941
1218R_03987
1218R_03994
irtB/1218R_04033
irtA/1218R_04034
1218R_04050
1218R_04051
1218R_04052
1218R_04053
1218R_04054
1218R_04055
lysA/1218R_04109
espA_2/1218R_04172
espC_1/1218R_04173

Fig S6D

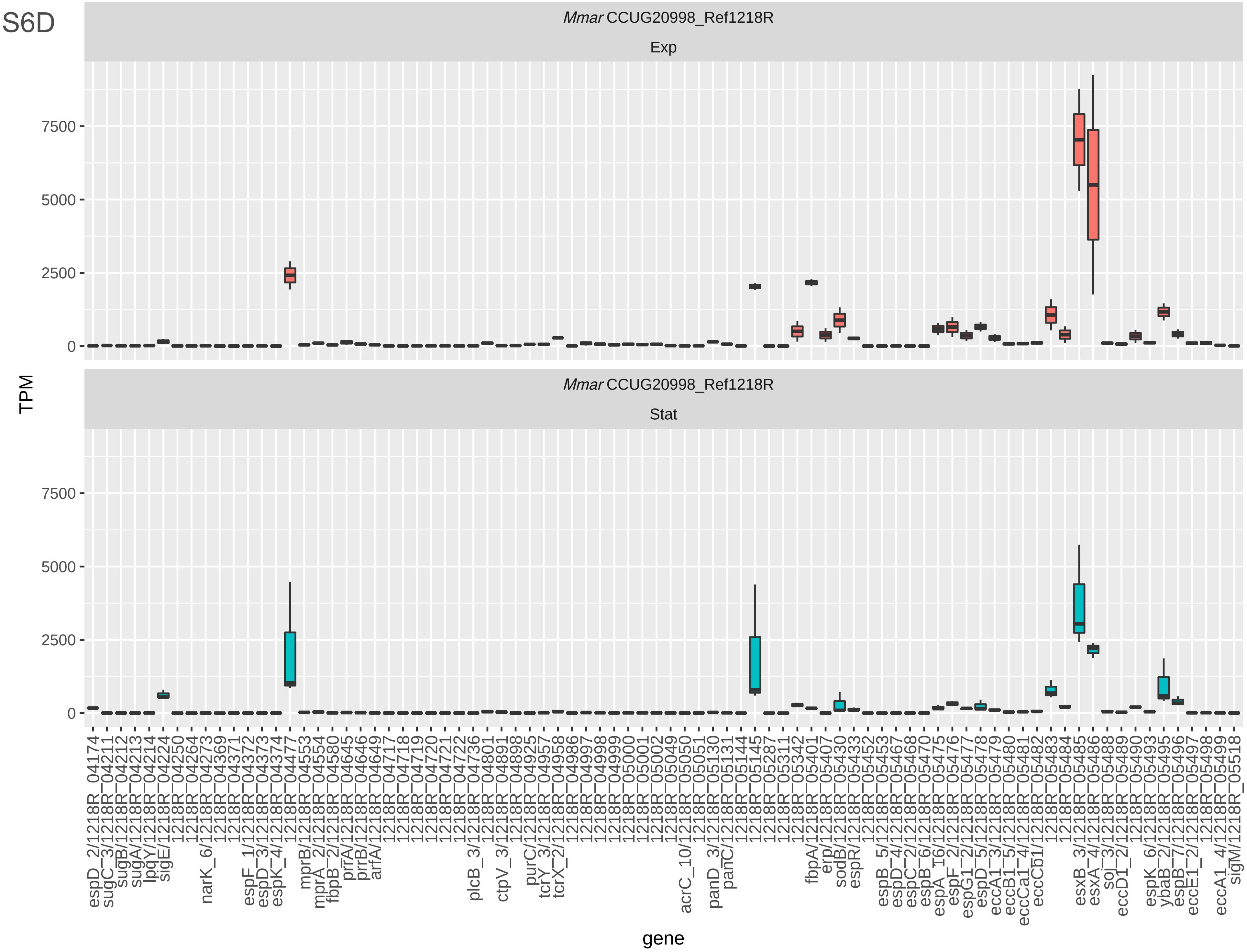

Mmar CCUG20998_Ref1218R
Exp
Mmar CCUG20998_Ref1218R
Stat
TPM
7500
5000
2500
0
gene

Fig S6E

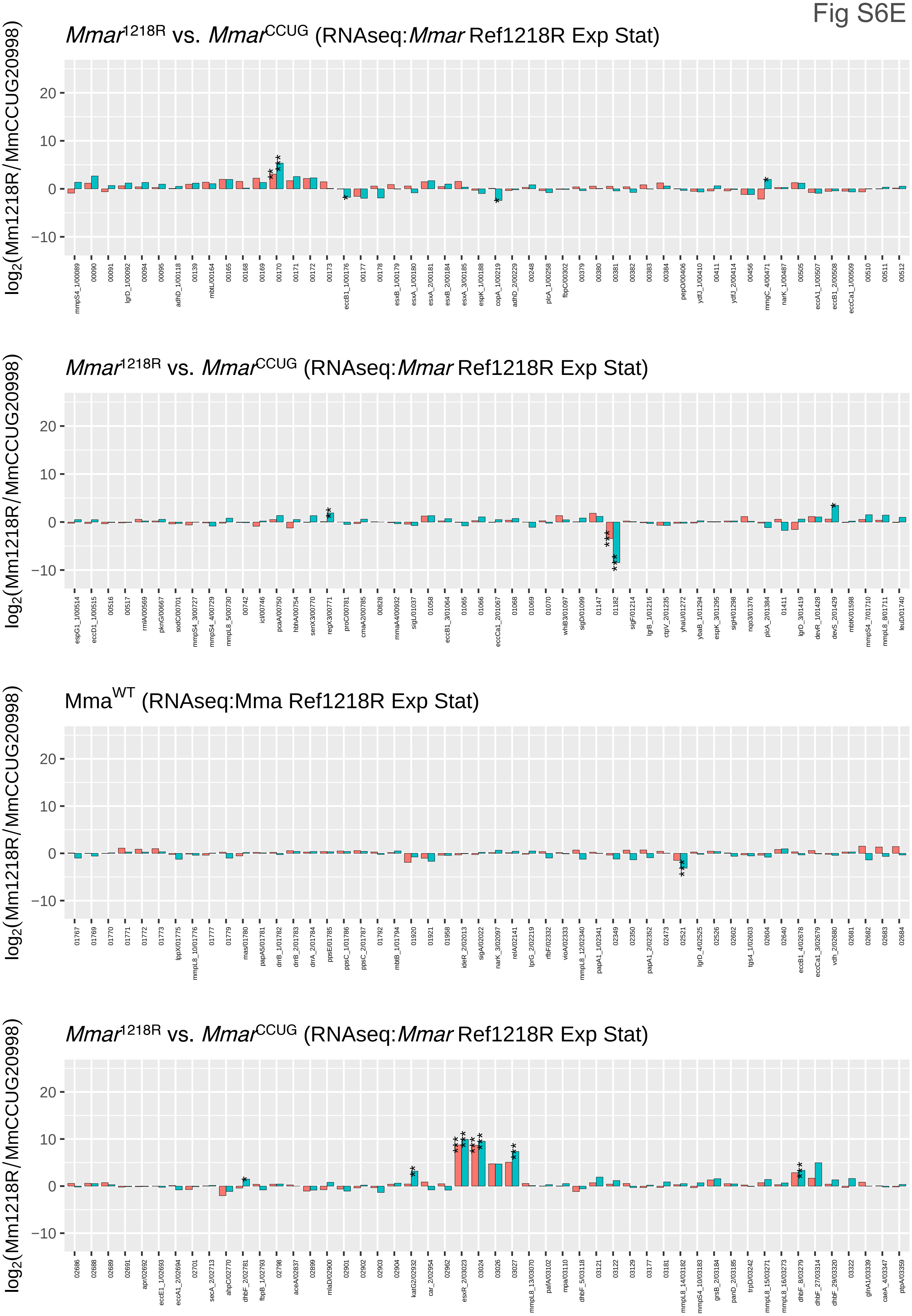

Mmar1218R vs. MmarCCUG (RNAseq:Mmar Ref1218R Exp Stat)
Mmar1218R vs. MmarCCUG (RNAseq:Mmar Ref1218R Exp Stat)
MmaWT (RNAseq:Mma Ref1218R Exp Stat)
Mmar1218R vs. MmarCCUG (RNAseq:Mmar Ref1218R Exp Stat)
log2(Mm1218R/MmCCUG20998)
20
10
0
−10

Fig S6E

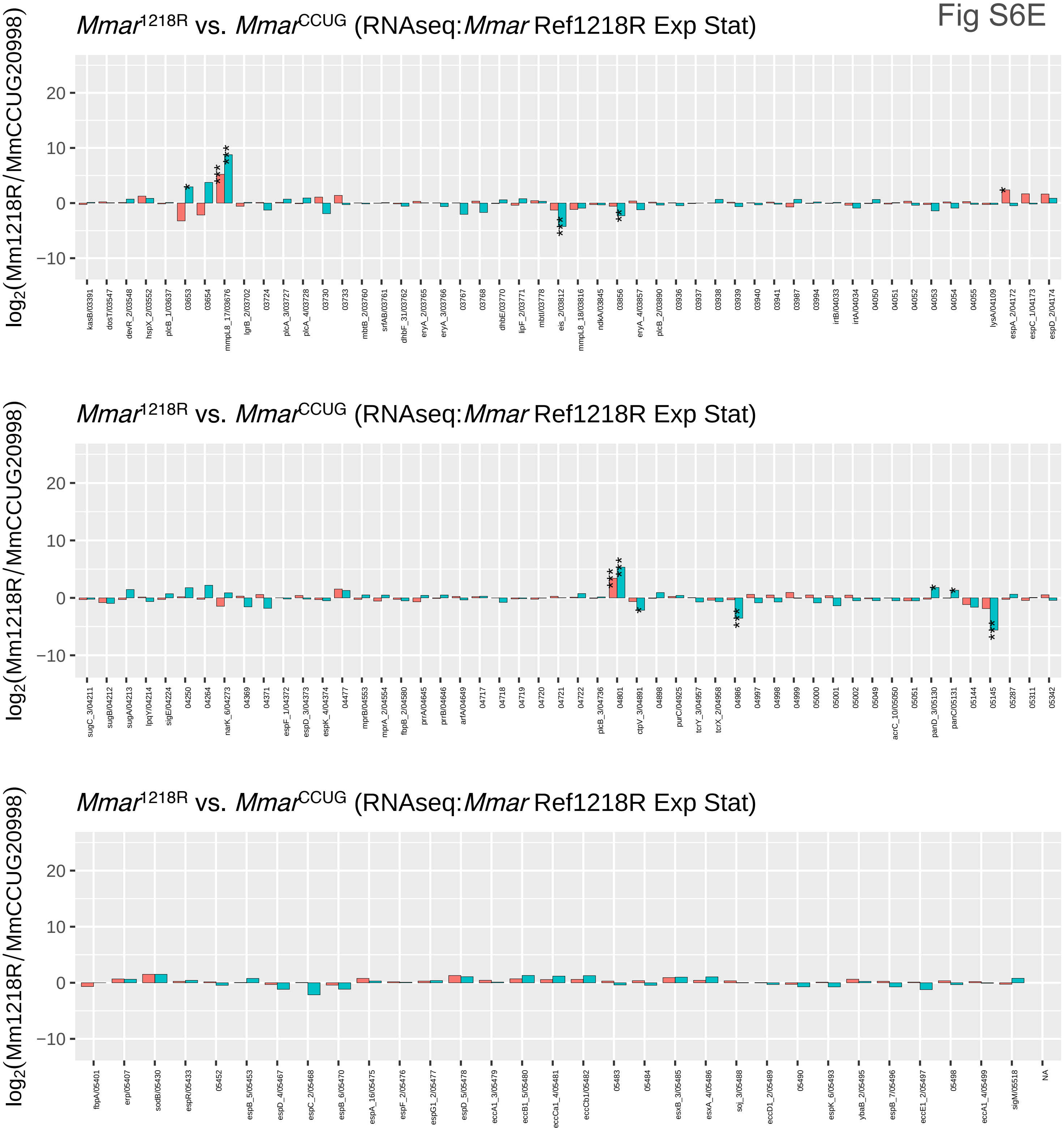
Mmar1218R vs. MmarCCUG (RNAseq:Mmar Ref1218R Exp Stat)
log2(Mm1218R/MmCCUG20998)
20
10
0
−10
kasB/03391
dosT/03547
devR_2/03548
hspX_2/03552
plcB_1/03637
03653
03654
mmpL8_17/03676
lgrB_2/03702
03724
plcA_3/03727
plcA_4/03728
03730
03733
mbtB_2/03760
srfAB/03761
dhbF_31/03762
eryA_2/03765
eryA_3/03766
03767
03768
dhbE/03770
lipF_2/03771
mbtI/03778
eis_2/03812
mmpL8_18/03816
ndkA/03845
03856
eryA_4/03857
plcB_2/03890
03936
03937
03938
03939
03940
03941
03987
03994
irtB/04033
irtA/04034
04050
04051
04052
04053
04054
04055
lysA/04109
espA_2/04172
espC_1/04173
espD_2/04174
Mmar1218R vs. MmarCCUG (RNAseq:Mmar Ref1218R Exp Stat)
log2(Mm1218R/MmCCUG20998)
20
10
0
−10
sugC_3/04211
sugB/04212
sugA/04213
lpqY/04214
sigE/04224
04250
04264
narK_6/04273
04369
04371
espF_1/04372
espD_3/04373
espK_4/04374
04477
mprB/04553
mprA_2/04554
fbpB_2/04580
prrA/04645
prrB/04646
arfA/04649
04717
04718
04719
04720
04721
04722
plcB_3/04736
04801
ctpV_3/04891
04898
purC/04925
tcrY_3/04957
tcrX_2/04958
04986
04997
04998
04999
05000
05001
05002
05049
acrC_10/05050
05051
panD_3/05130
panC/05131
05144
05145
05287
05311
05342
Mmar1218R vs. MmarCCUG (RNAseq:Mmar Ref1218R Exp Stat)
log2(Mm1218R/MmCCUG20998)
20
10
0
−10
fbpA/05401
erp/05407
sodB/05430
espR/05433
05452
espB_5/05453
espD_4/05467
espC_2/05468
espB_6/05470
espA_16/05475
espF_2/05476
espG1_2/05477
espD_5/05478
eccA1_3/05479
eccB1_5/05480
eccCa1_4/05481
eccCb1/05482
05483
05484
esxB_3/05485
esxA_4/05486
soj_3/05488
eccD1_2/05489
05490
espK_6/05493
ybaB_2/05495
espB_7/05496
eccE1_2/05497
05498
eccA1_4/05499
sigM/05518
NA

Fig S6F

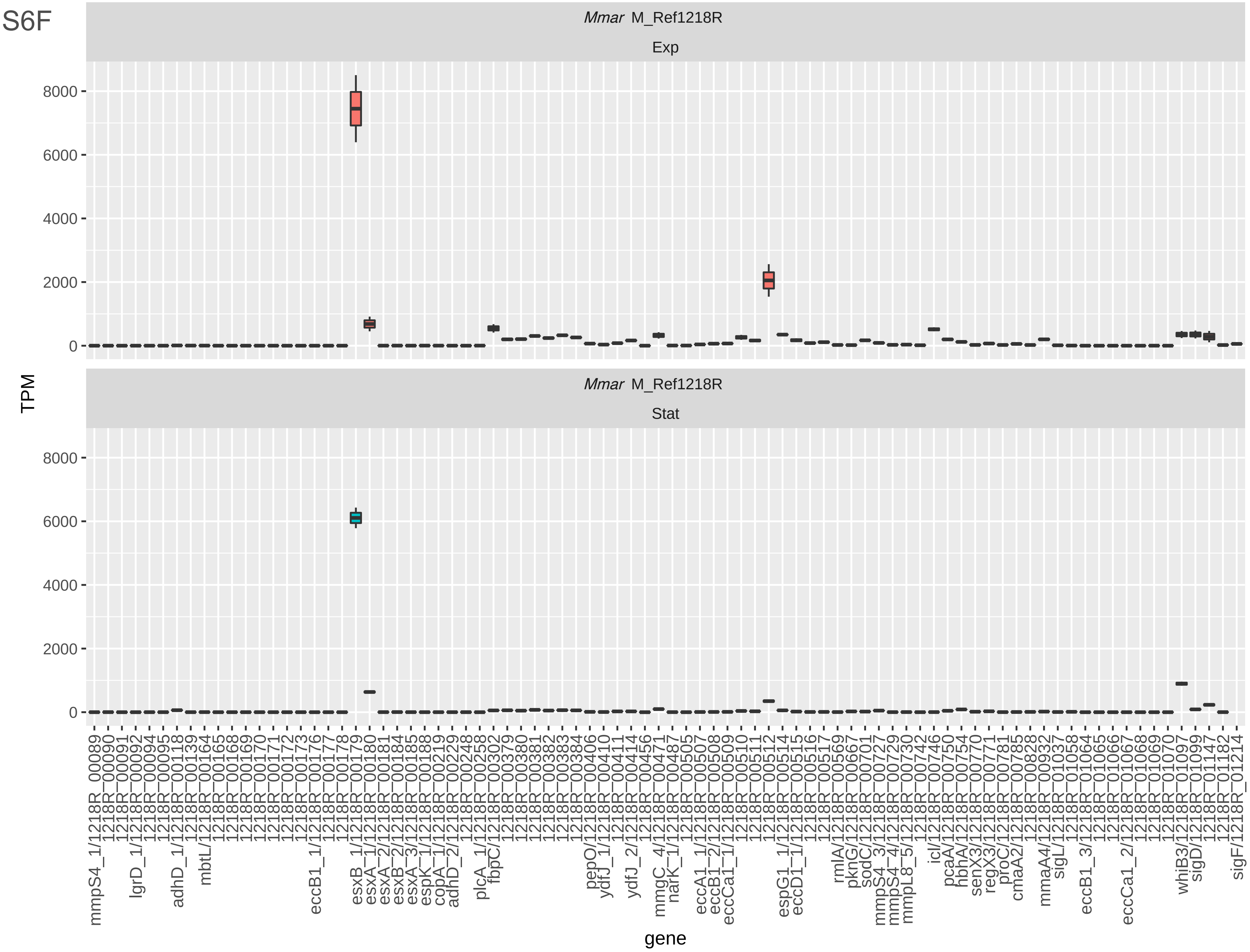
Mmar M_Ref1218R
Exp
Mmar M_Ref1218R
Stat
TPM
8000
6000
4000
2000
0
gene

Fig S6F

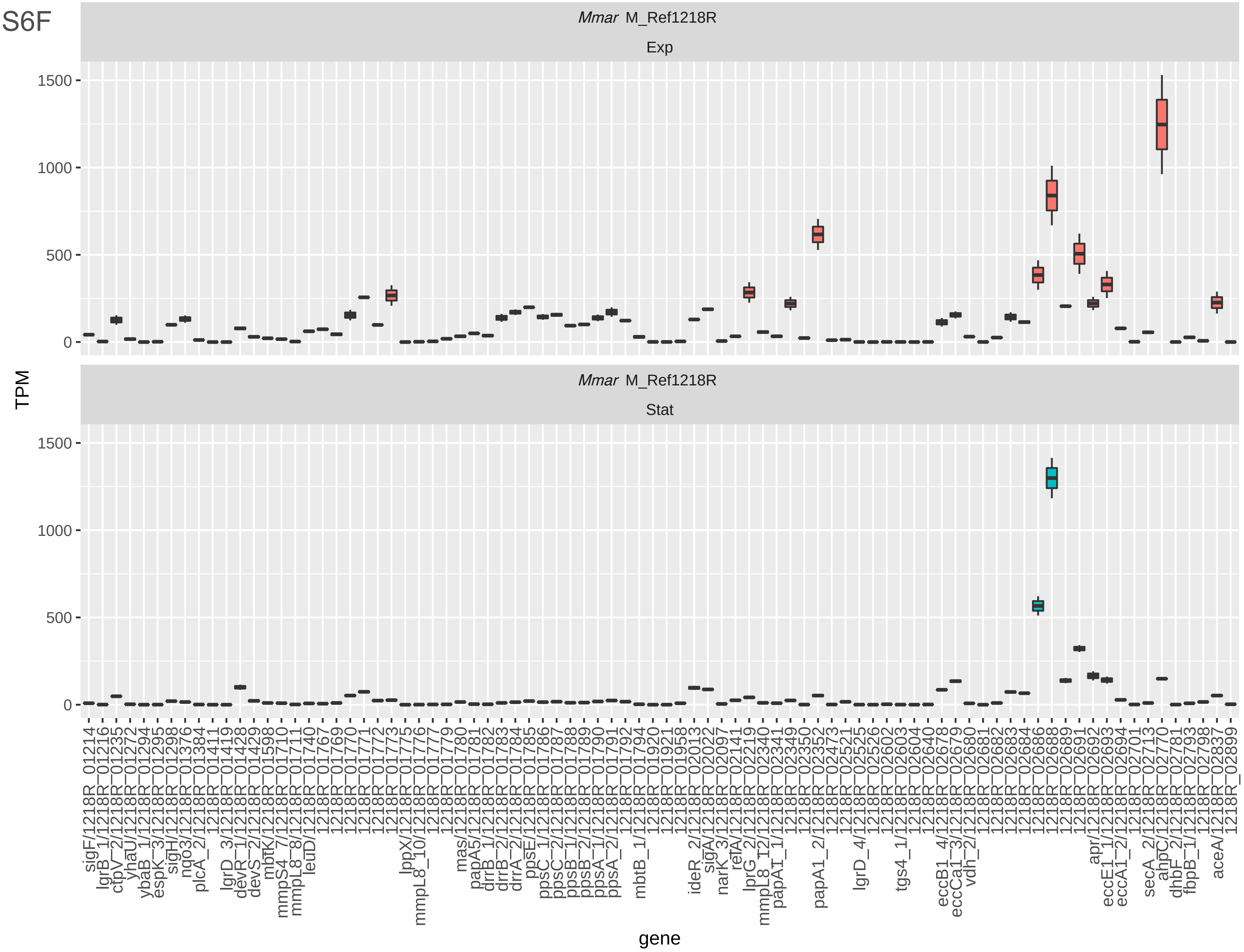

Mmar M_Ref1218R
Exp
Mmar M_Ref1218R
Stat
TPM
gene
1500
1000
500
0

Fig S6F

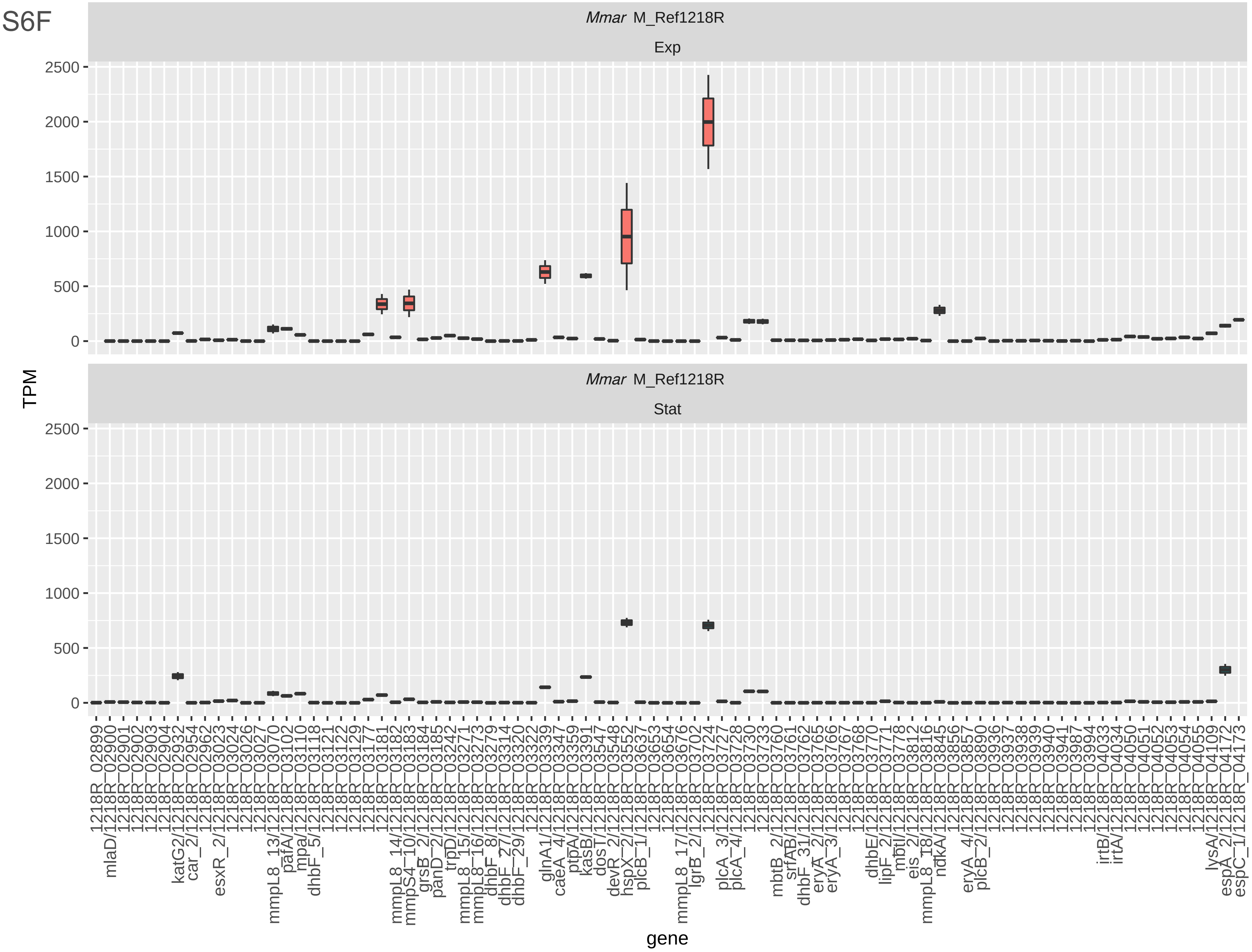
Mmar M_Ref1218R
Exp
Mmar M_Ref1218R
Stat
TPM
2500
2000
1500
1000
500
0
gene

Fig S6F

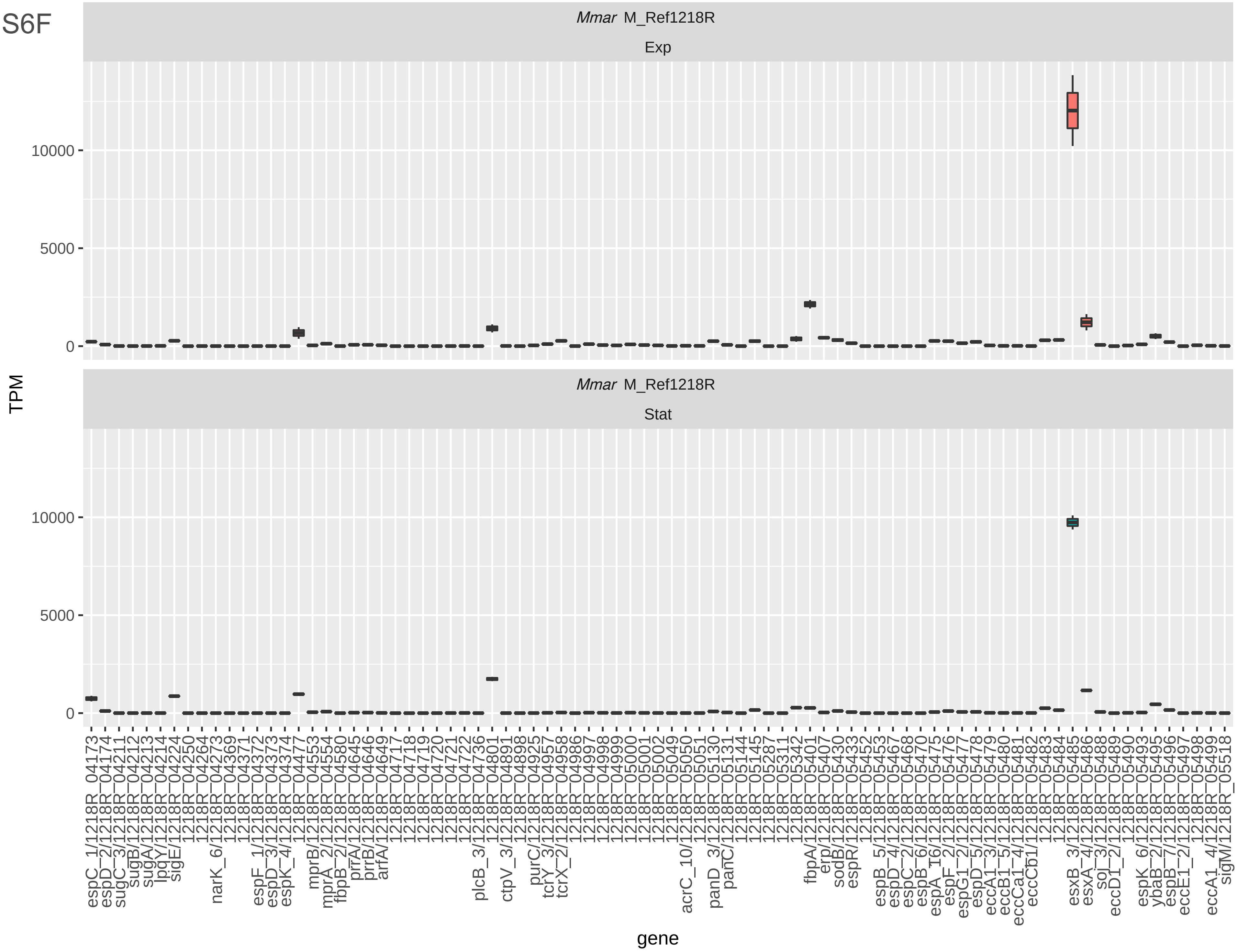

Mmar M_Ref1218R
Exp
Mmar M_Ref1218R
Stat
10000
5000
0
TPM
gene

Fig S6G

Mmar[1218R] vs. *Mmar*[M] (RNAseq:*Mmar* Ref1218R Exp Stat)

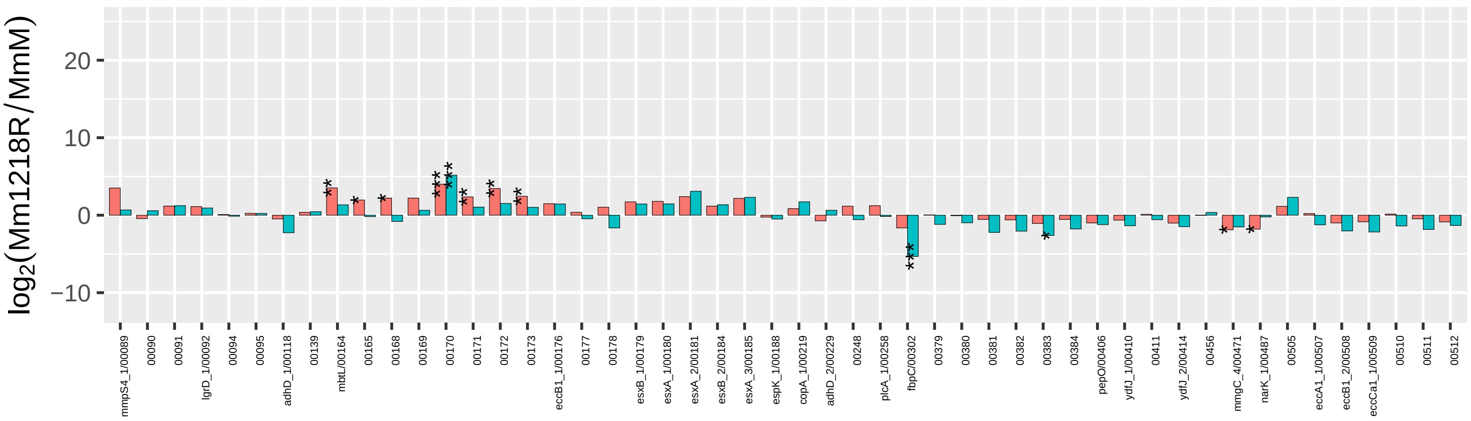

Mmar[1218R] vs. *Mmar*[M] (RNAseq:*Mmar* Ref1218R Exp Stat)

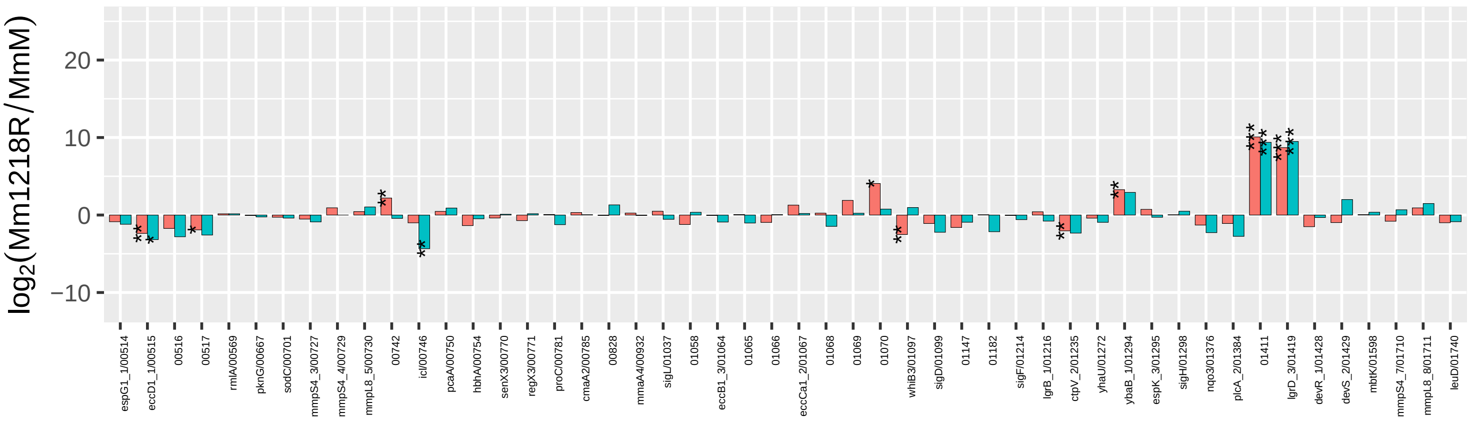

Mmar[1218R] vs. *Mmar*[M] (RNAseq:*Mmar* Ref1218R Exp Stat)

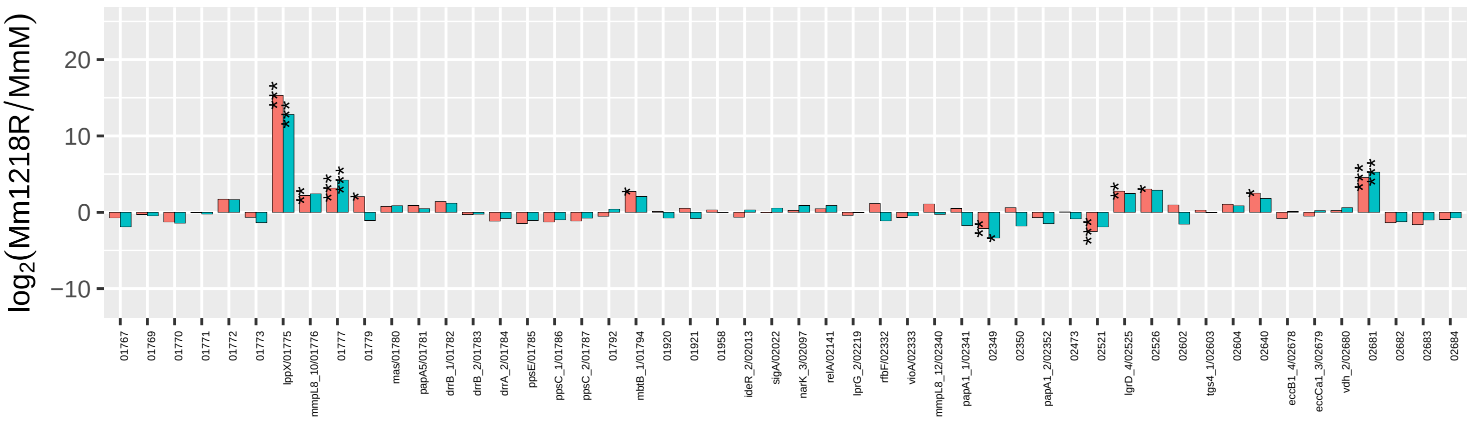

Mmar[1218R] vs. *Mmar*[M] (RNAseq:*Mmar* Ref1218R Exp Stat)

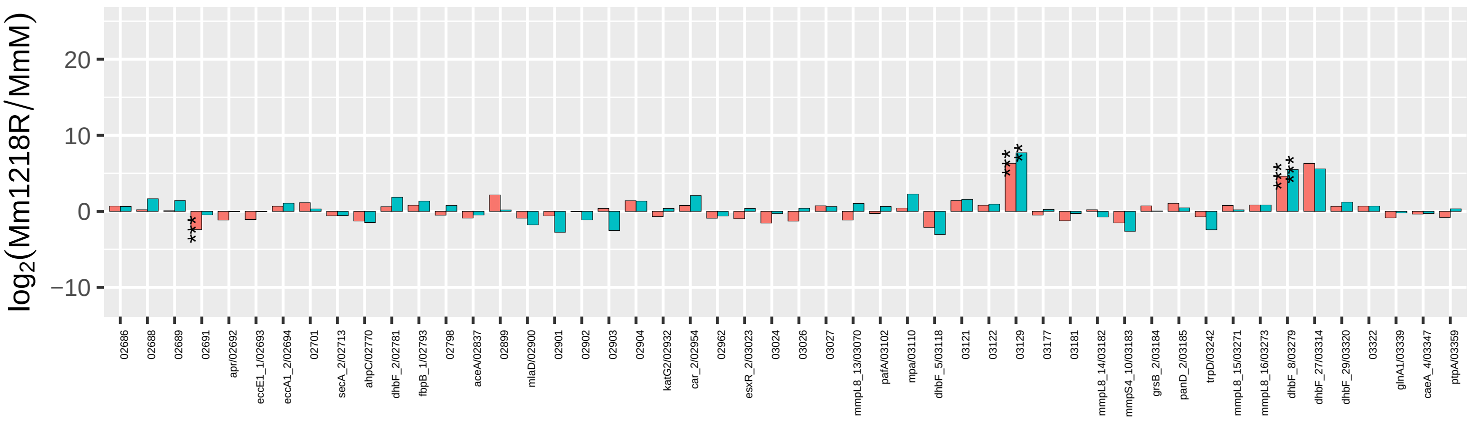

Fig S6G

*Mmar*1218R vs. *Mmar*M (RNAseq:*Mmar* Ref1218R Exp Stat)

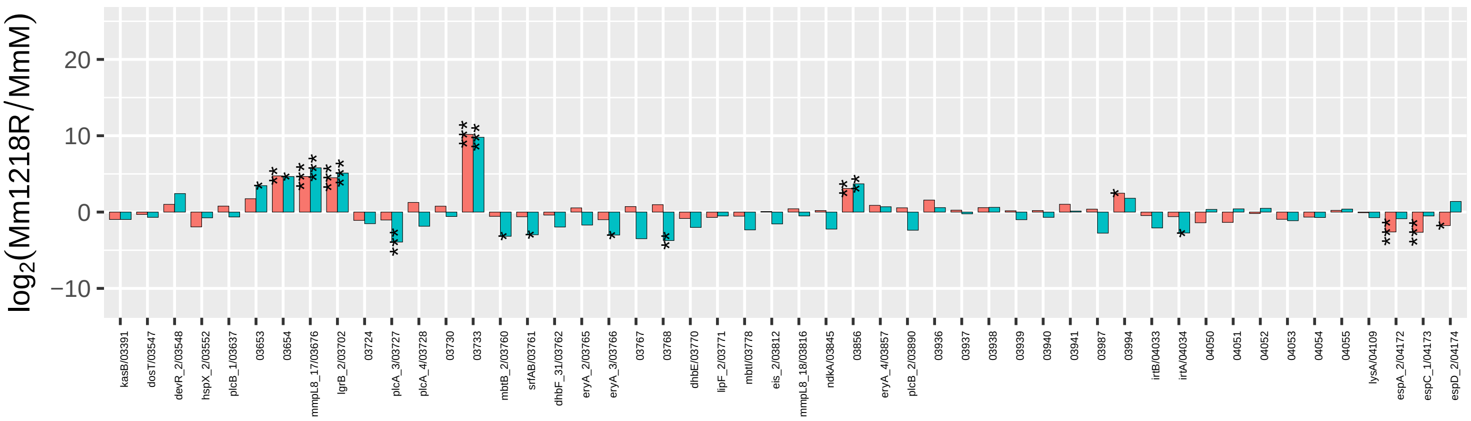

*Mmar*1218R vs. *Mmar*M (RNAseq:*Mmar* Ref1218R Exp Stat)

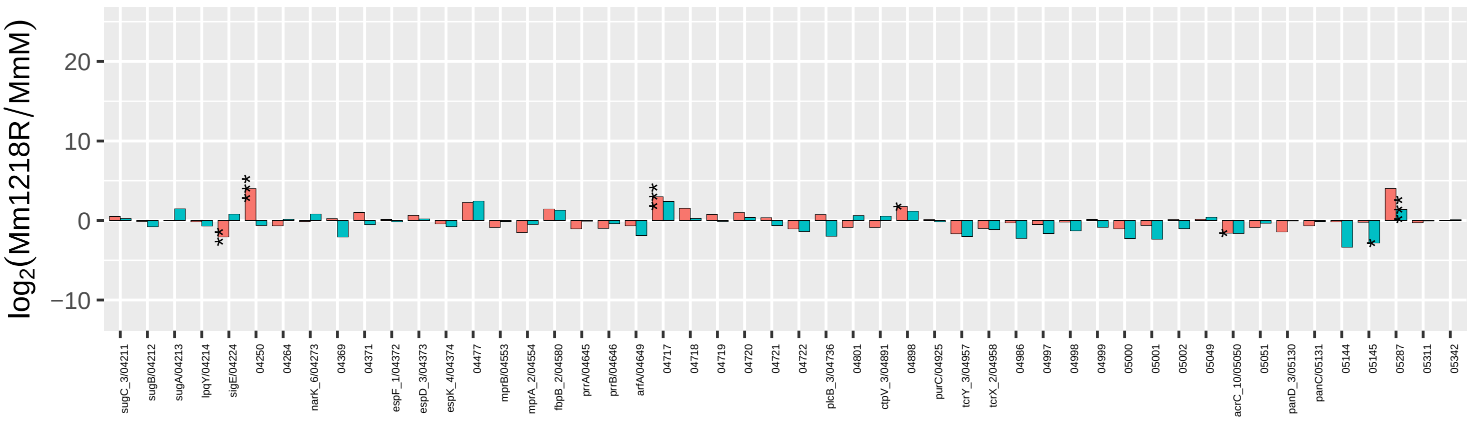

*Mmar*1218R vs. *Mmar*M (RNAseq:*Mmar* Ref1218R Exp Stat)

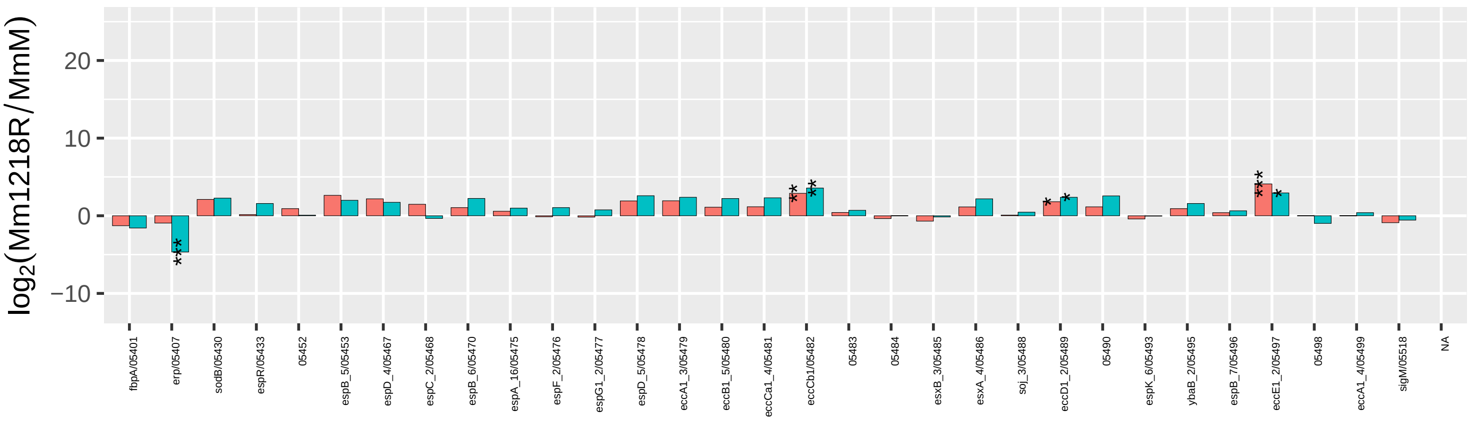

Figure S7 Bar plots showing transcription of ESX-1, ESX-3, ESX-4, ESX-5 and ESX-6 genes in 1218R, 1218S, *Mmar*^{CCUG} and *Mmar*^{M}.

(a) Transcript levels (distribution) in exponentially growing 1218R cells.

(b) Transcript levels (distribution) in stationary 1218R cells.

(c) Change in transcript levels, expressed as log_2-fold change, comparing levels in exponentially growing and stationary 1218R cells. A negative log_2-value suggests that the corresponding mRNA is more abundant in exponentially growing cells while a positive value suggests higher levels in stationary cells. Statistical significance, see Materials and Methods; *p-value < 0.05; **p-value < 0.01; ***p-value < 0.001.

(d) Transcript levels (distribution) in exponentially growing 1218S cells.

(e) Transcript levels (distribution) in stationary 1218S cells.

(f) Change in transcript levels, expressed as log_2-fold change, comparing levels in exponentially growing and stationary 1218S cells. A negative log_2-value suggests that the corresponding mRNA is more abundant in exponentially growing cells while a positive value suggests higher levels in stationary cells. Statistical significance, see Materials and Methods; *p-value < 0.05; **p-value < 0.01; ***p-value < 0.001.

(g) Transcript levels (distribution) in exponentially growing *Mmar*^{CCUG} cells.

(h) Transcript levels (distribution) in stationary *Mmar*^{CCUG} cells.

(i) Change in transcript levels, expressed as log_2-fold change, comparing levels in exponentially growing and stationary *Mmar*^{CCUG} cells. A negative log_2-value suggests that the corresponding mRNA is more abundant in exponentially growing cells while a positive value suggests higher levels in stationary cells. Statistical significance, see Materials and Methods; *p-value < 0.05; **p-value < 0.01; ***p-value < 0.001.

(j) Transcript levels (distribution) in exponentially growing *Mmar*^{M} cells.

(k) Transcript levels (distribution) in exponentially growing *Mmar*^{M} cells.

(l) Change in transcript levels, expressed as log_2-fold change, comparing levels in exponentially growing and stationary *Mmar*^{M} cells. A negative log_2-value suggests that the corresponding mRNA is more abundant in exponentially growing cells while a positive value suggests higher levels in stationary cells.

Statistical significance, see Materials and Methods; *p-value < 0.05; **p-value < 0.01; ***p-value < 0.001.

Figure S7A-C

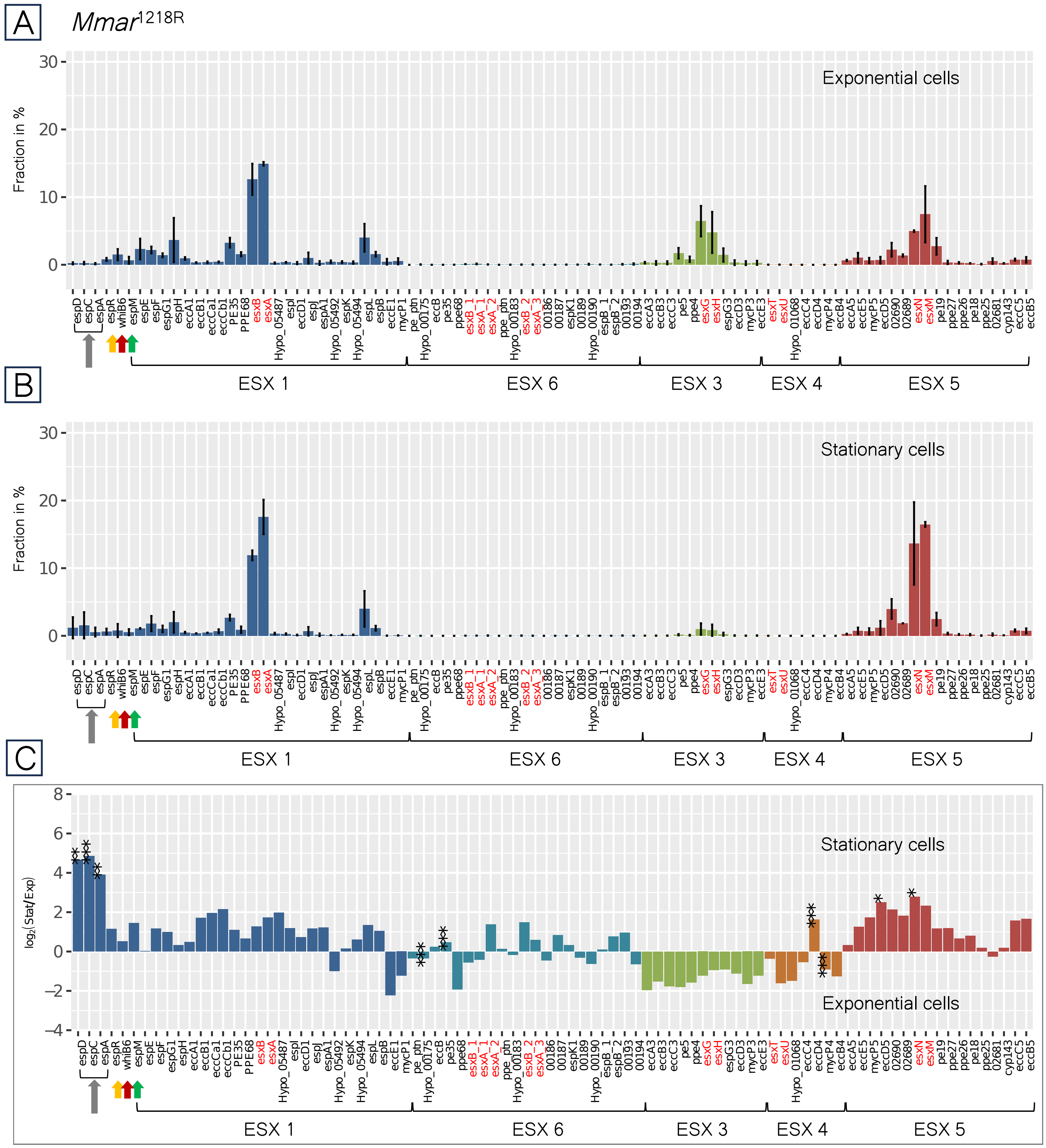
A
*Mmar*1218R
Exponential cells
Fraction in %
B
Stationary cells
Fraction in %
C
Stationary cells
Exponential cells
log2(Stat/Exp)
ESX 1
ESX 6
ESX 3
ESX 4
ESX 5

Figure S7D-F

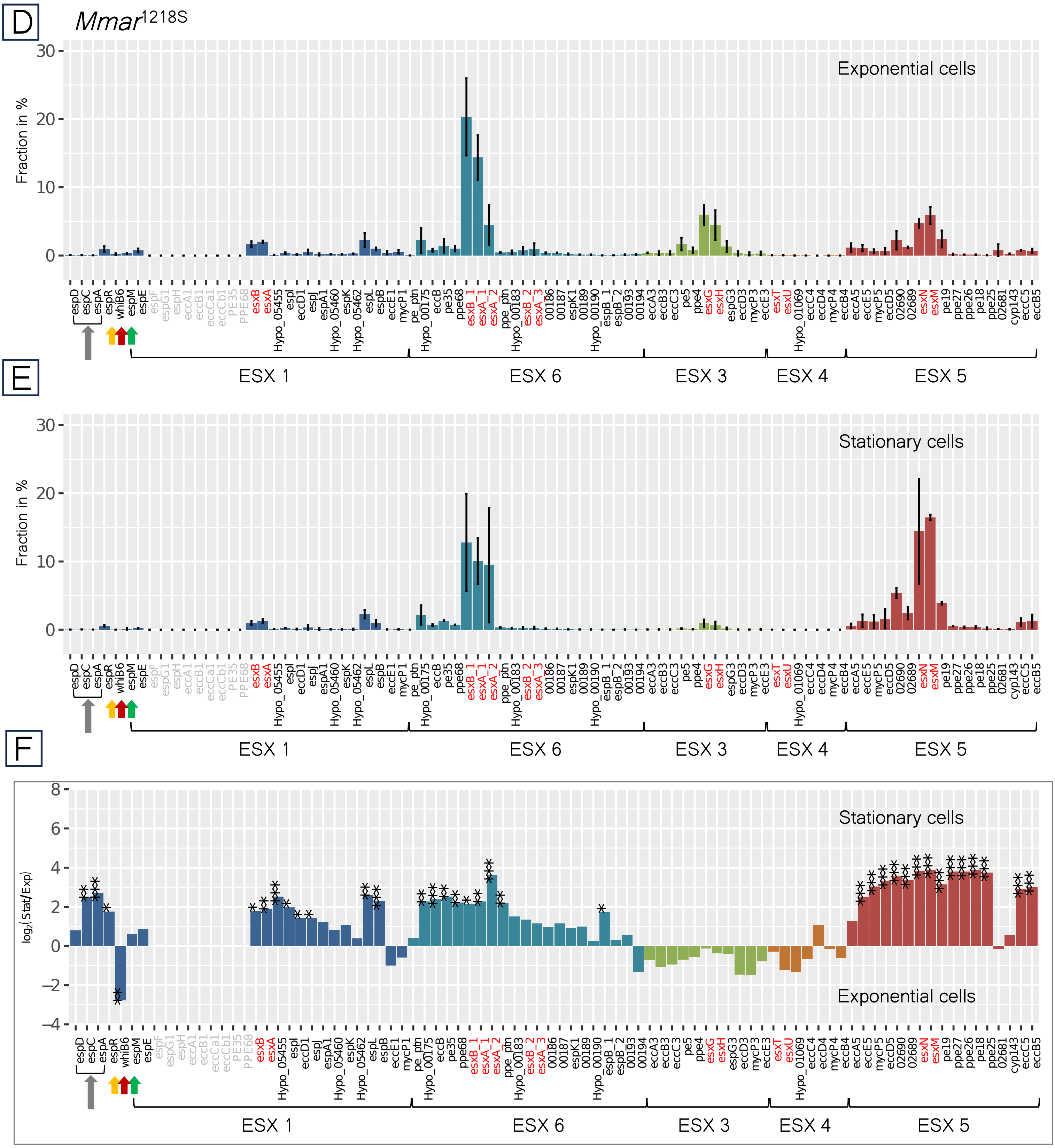
D
Mmar1218S
Exponential cells
Fraction in %
30
20
10
0
espD espC espA espR whiB6 espM espE espF espG1 espH eccA1 eccB1 eccCa1 eccCb1 PE35 PPE68 esxB esxA Hypo_05455 espI eccD1 espJ espA1 Hypo_05460 espK Hypo_05462 espL espB eccE1 mycP1 pe_pth Hypo_00175 eccB pe35 ppe68 esxB_1 esxA_1 esxA_2 ppe_pth Hypo_00183 esxB_2 esxA_3 00186 00187 espK1 00189 Hypo_00190 espB_1 espB_2 00193 00194 eccA3 eccB3 eccC3 pe5 ppe4 esxG esxH espG3 eccD3 mycP3 eccE3 esxT esxU Hypo_01069 eccC4 eccD4 mycP4 eccB4 eccA5 eccE5 mycP5 eccD5 02690 02689 esxN esxM pe19 ppe27 ppe26 pe18 ppe25 02681 cyp143 eccC5 eccB5
ESX 1
ESX 6
ESX 3
ESX 4
ESX 5
E
Stationary cells
Fraction in %
F
Stationary cells
Exponential cells
log2(Stat/Exp)
8
6
4
2
0
−2
−4

Figure S7G -I

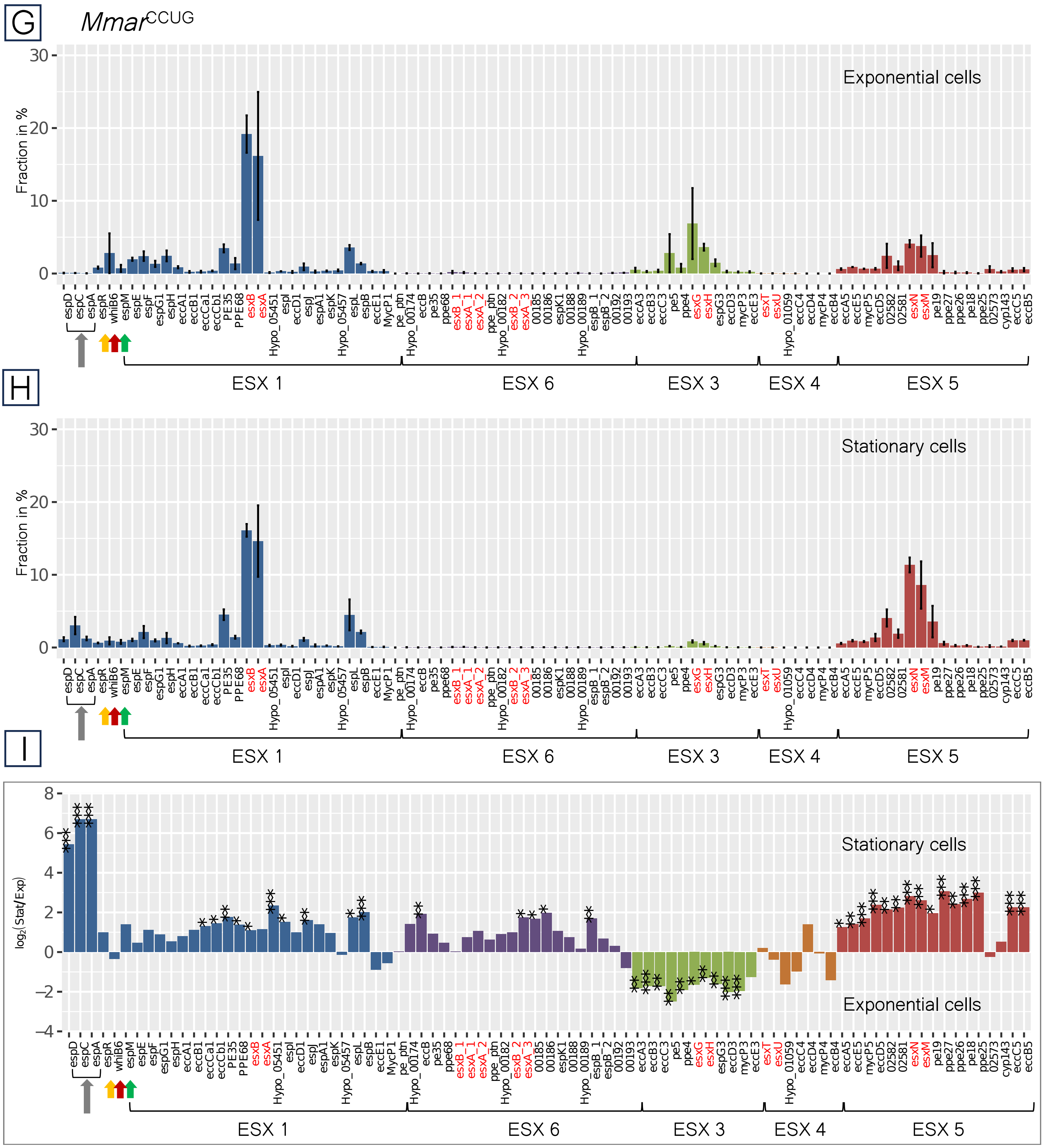
G
Mmar^{CCUG}
Exponential cells
Fraction in %
H
Stationary cells
Fraction in %
I
Stationary cells
Exponential cells
$log_2(Stat/Exp)$
ESX 1
ESX 6
ESX 3
ESX 4
ESX 5
espD
espC
espA
espR
whiB6
espM
espE
espF
espG1
espH
eccA1
eccB1
eccCa1
eccCb1
PE35
PPE68
esxB
esxA
Hypo_05451
espI
eccD1
espJ
espA1
espK
Hypo_05457
espL
espB
eccE1
MycP1
pe_ptn
Hypo_00174
eccB
pe35
ppe68
esxB_1
esxA_1
esxA_2
ppe_ptn
Hypo_00182
esxB_2
esxA_3
00185
00186
espK1
00188
Hypo_00189
espB_1
espB_2
00192
00193
eccA3
eccB3
eccC3
pe5
ppe4
esxG
esxH
espG3
eccD3
mycP3
eccE3
esxT
esxU
Hypo_01059
eccC4
mycP4
eccB4
eccA5
eccE5
mycP5
eccD5
02582
02581
esxN
esxM
pe19
ppe27
ppe26
pe18
ppe25
02573
cyp143
eccC5
eccB5

Figure S7J -L

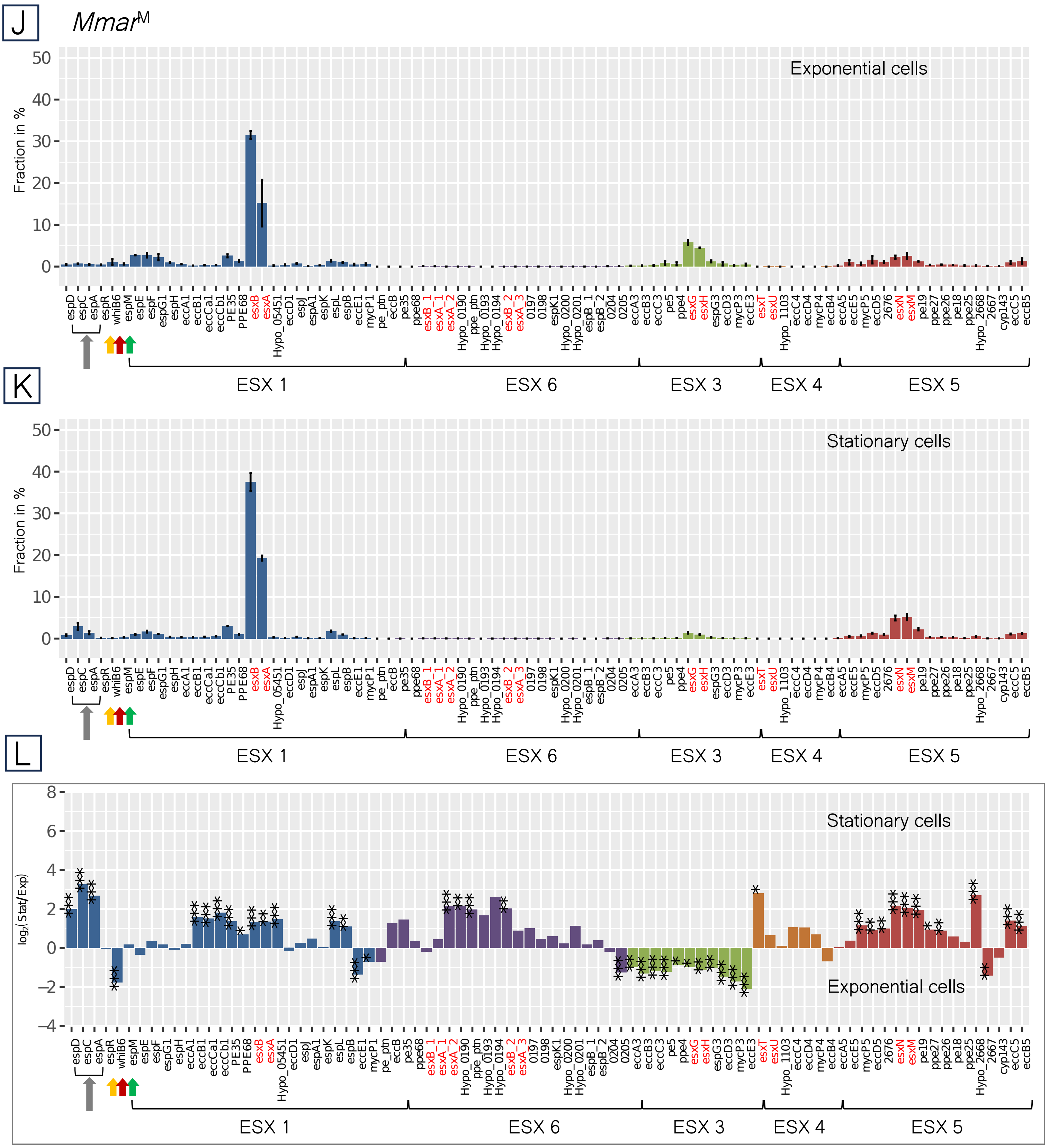
J
MmarM
Exponential cells
Fraction in %
K
Stationary cells
Fraction in %
L
Stationary cells
Exponential cells
log2(Stat/Exp)
ESX 1
ESX 6
ESX 3
ESX 4
ESX 5

Figure S8 RNA-Seq reads showing transcription of *espF-esxB* in 1218S. The 1218R gene annotations/identity used as reference.

Fig S8 RNA-Seq – 1218S *espF-esxB*

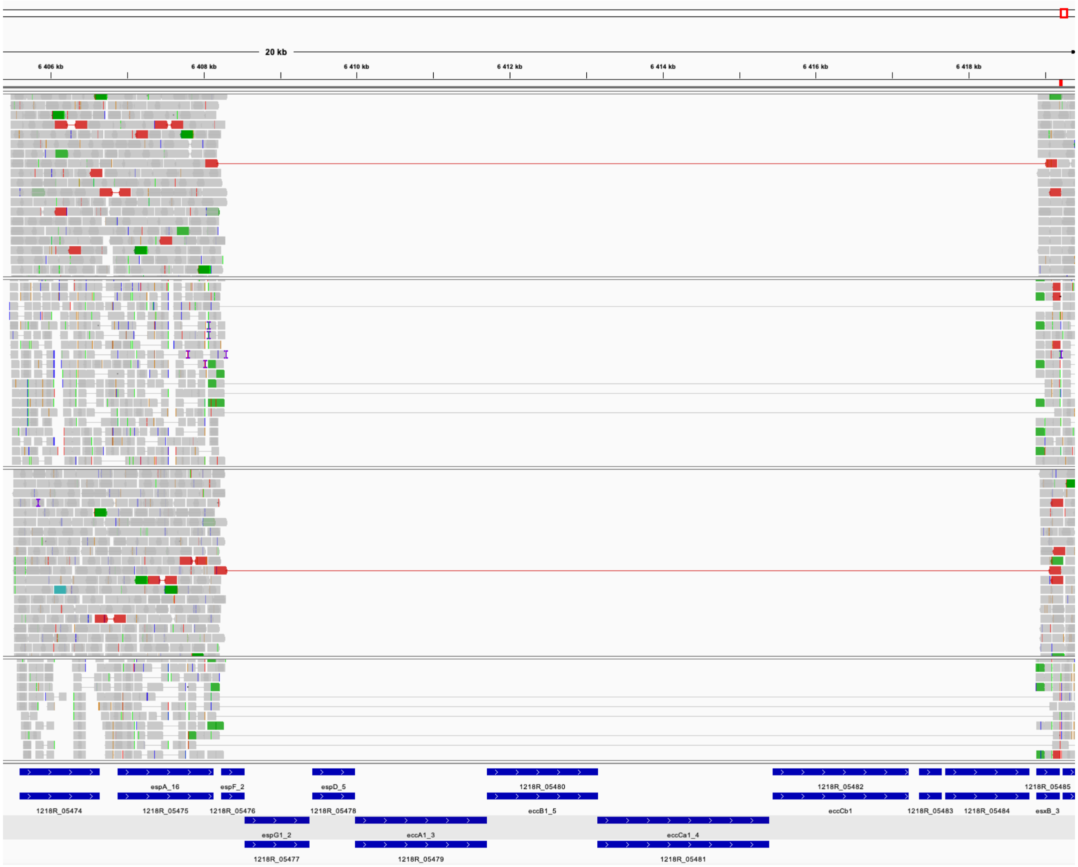

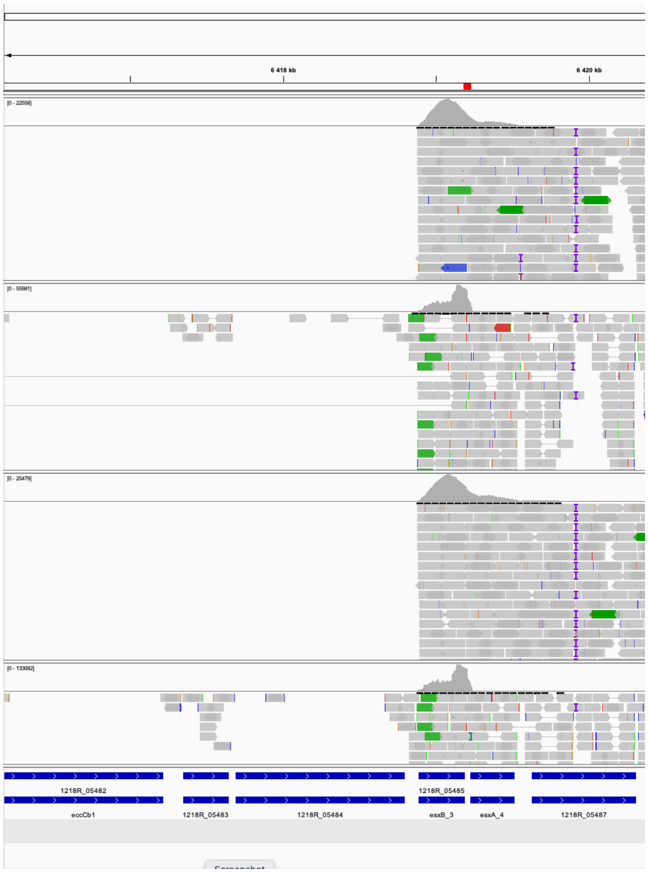

Figure S9 Transcript levels of LOS genes in 1218R and 1218S.

(a) Transcript levels (distribution, expressed in TPM values) in exponentially growing and stationary 1218R cells.

(b) Comparing transcript levels in exponentially growing and stationary 1218R cells. Negative $\log_2$-values suggest that the corresponding mRNA is more abundant in exponential cells while a positive value suggests higher levels in stationary 1218R cells.

(c) Transcript levels (distribution, expressed in TPM values) in exponentially growing and stationary 1218S cells.

(d) Comparing transcript levels in exponentially growing and stationary 1218S cells. Negative $\log_2$-values suggest that the corresponding mRNA is more abundant in exponential cells while a positive value suggests higher levels in stationary 1218S cells. The 1218R gene annotations/identity used as reference.

Statistical significance, see Materials and Methods; *p-value < 0.05; **p-value < 0.01; ***p-value < 0.001.

Fig S9A-B

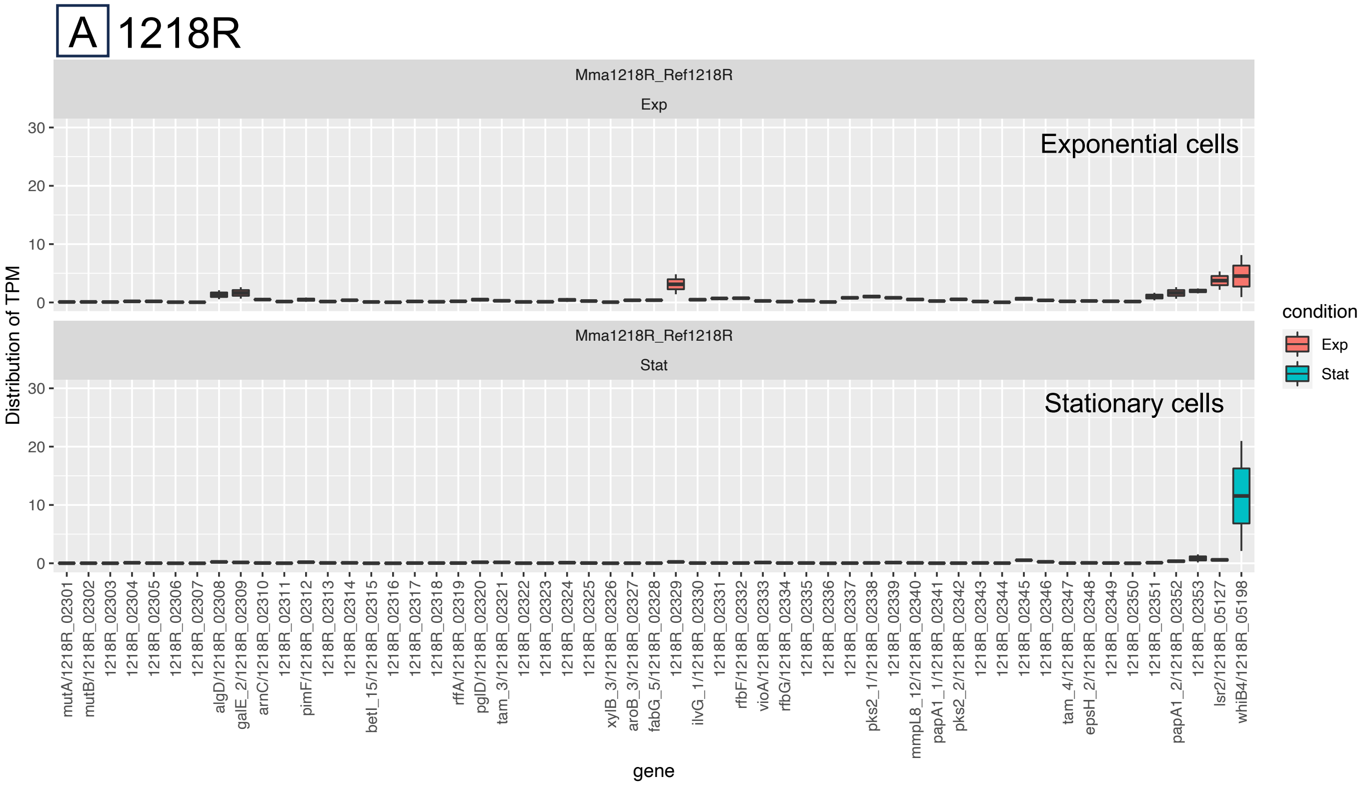

A 1218R
Mma1218R_Ref1218R
Exp
Exponential cells
Mma1218R_Ref1218R
Stat
Stationary cells
Distribution of TPM
condition
Exp
Stat
gene

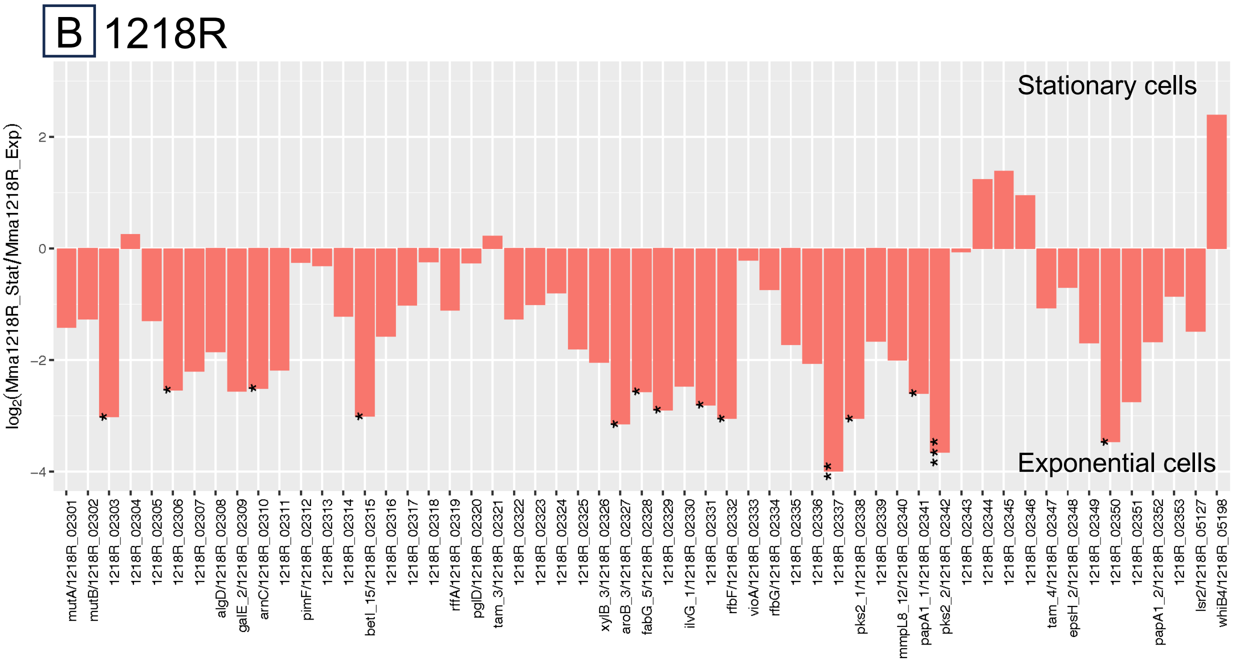

B 1218R
Stationary cells
Exponential cells
log2(Mma1218R_Stat/Mma1218R_Exp)

Fig S9C-D

C 1218S

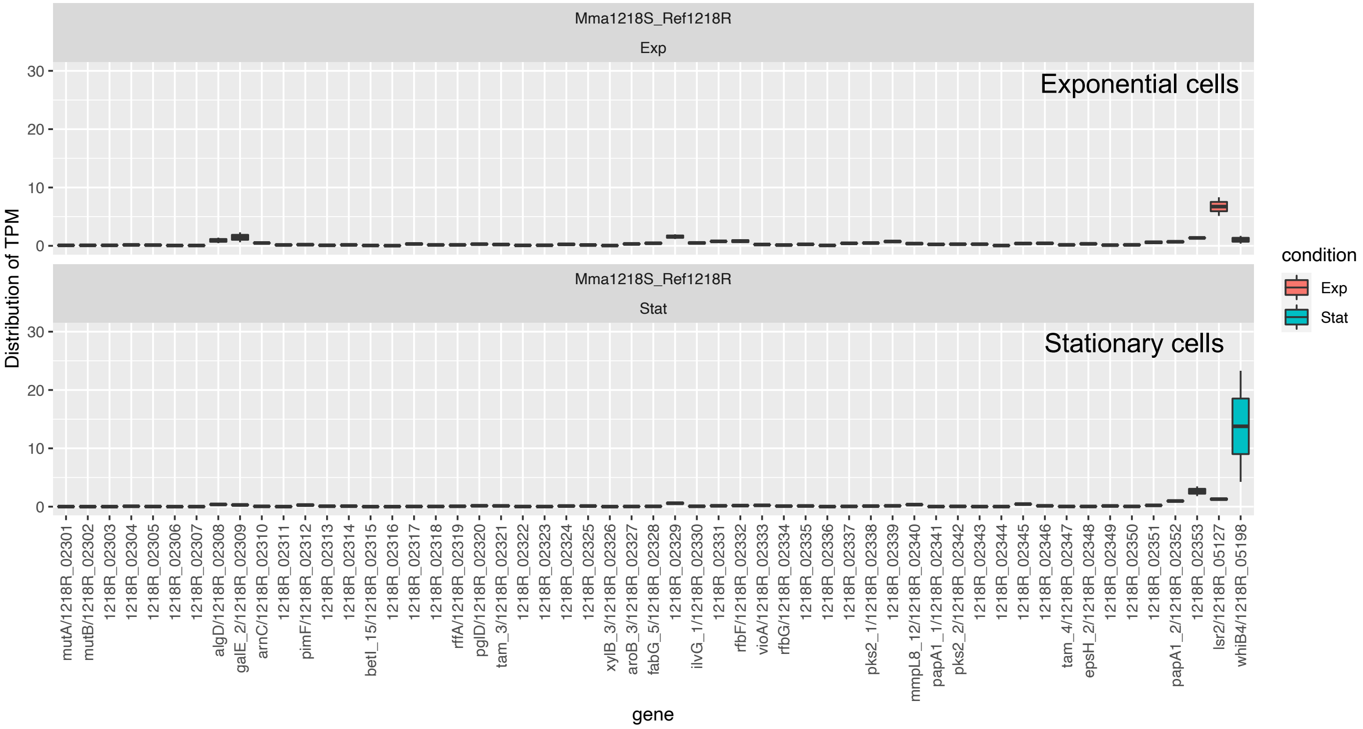

D 1218S

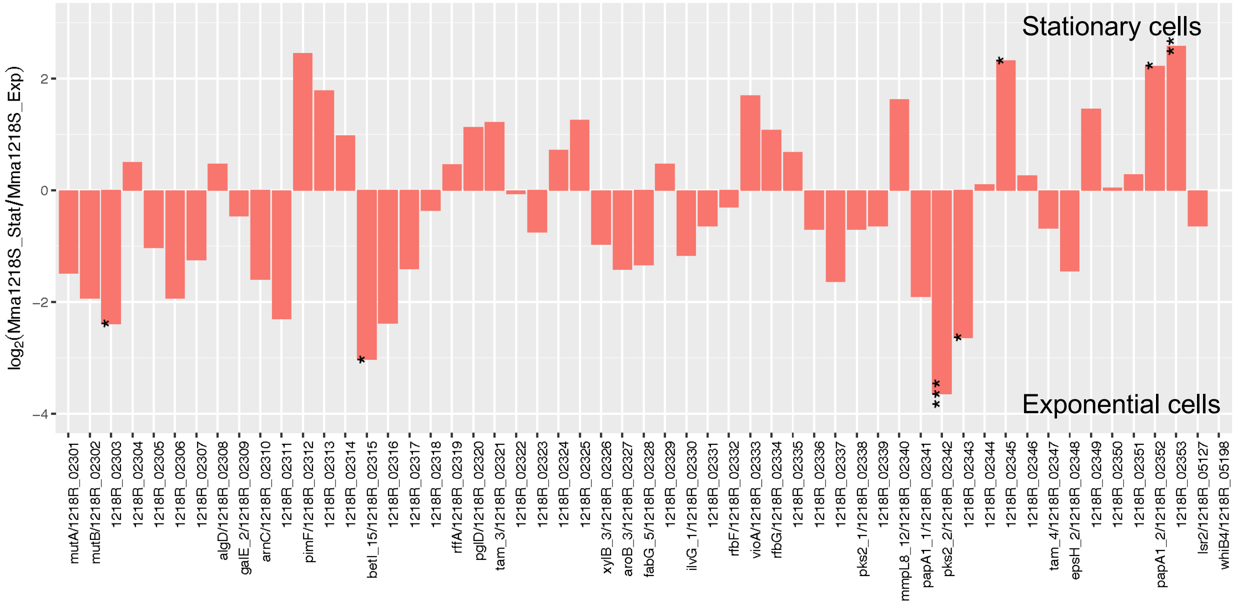

Figure S10 Ms1 RNA gene transcript levels.

Ms1 RNA levels in exponentially growing and stationary 1218R and 1218S cells (left panel); *Mmar*^{CCUG} and *Mmar*^{M} (see also Figure 7a). Levels with postive values correspond to higher levels in stationary cells.

Statistical significance, see Materials and Methods; *p-value < 0.05; **p-value < 0.01; ***p-value < 0.001.

Fig S10

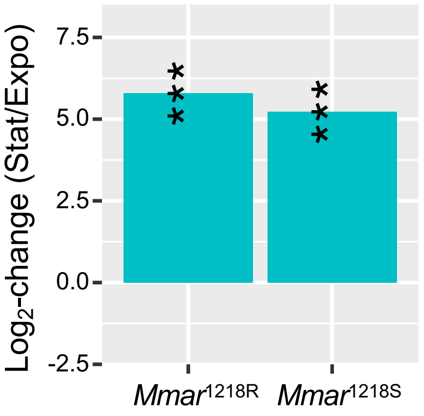

Log2-change (Stat/Expo)
7.5
5.0
2.5
0.0
-2.5
***
***
Mmar1218R
Mmar1218S

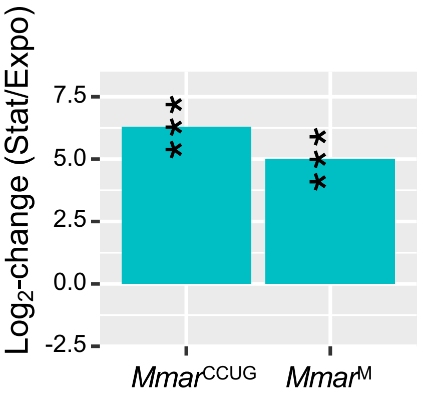

Log2-change (Stat/Expo)
7.5
5.0
2.5
0.0
-2.5
***
***
MmarCCUG
MmarM

Figure S11 Growth on 7H10 media after transformation of 1218R and 1218S with the empty control plasmid (pBS401) or with pBS401$^{espF\text{-}H}$ as indicated (uninduced, top panels; induced with tetracycline, lower panels). For details see main text, Figure 8 and Materials and Methods.

Fig S11

Uninduced

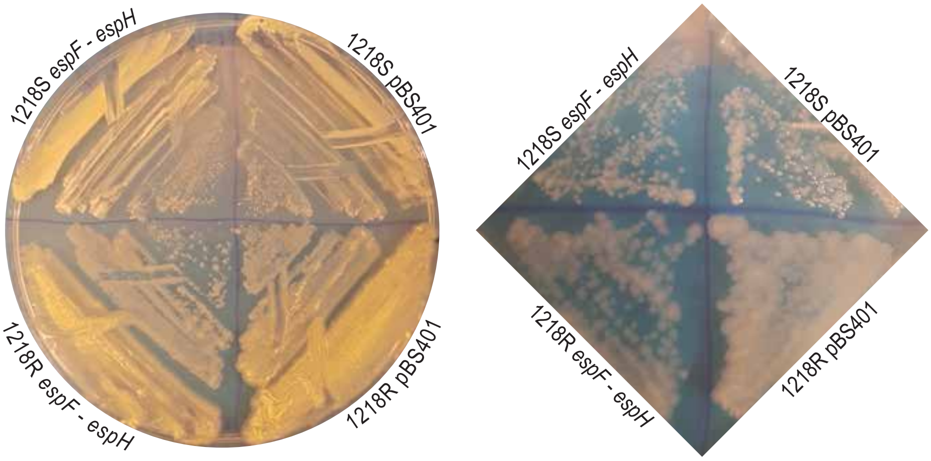

Induced

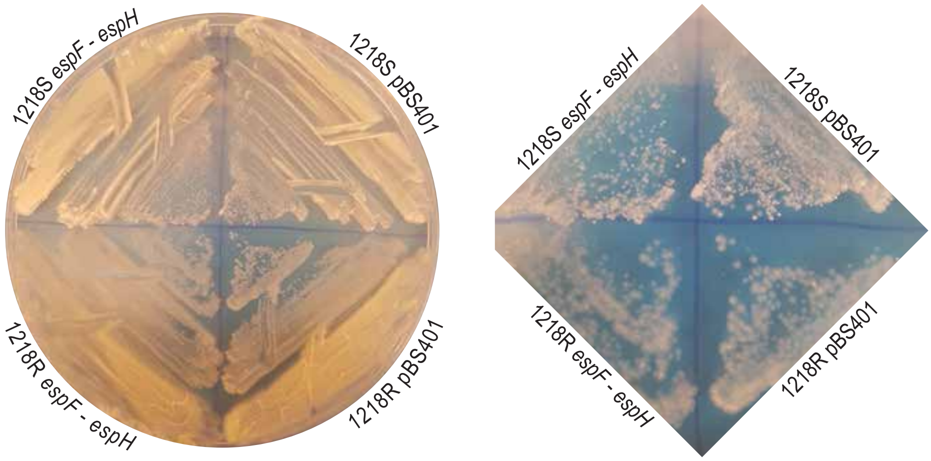

Supplement: Supplementary file 1 — Supplementary Information 1. [file 41598_2026_61405_MOESM1_ESM.pdf]
